# Application of LLM combined with knowledge base in improving C-TIRADS grading of thyroid nodules

**DOI:** 10.3389/fendo.2026.1888772

**Published:** 2026-10-15

**Authors:** Qian Qu, Hanwen Bi, Xiaofei Yu, Rui Zhou, Zhen Liu, Tengteng Hao

**Affiliations:** 1 School of Traditional Chinese Medicine, Shandong Medical And Pharmaceutical University, Yantai, China; 2 Department of Ultrasound, Yantai Fushan District People's Hospital, Yantai, China; 3 Department of Oncology, Xiyuan Hospital of China Academy of Chinese Medical Sciences, Beijing, China; 4 Department of Ultrasound Medicine, The Affiliated Yantai Yuhuangding Hospital of Qingdao University, Yantai, Shandong, China

**Keywords:** artificial intelligence, C-TIRADS classification, FastGPT knowledge base system, large language model, papillary thyroid carcinoma, thyroid nodule

## Abstract

**Objective:**

This study used the unified text-based grading results of senior sonographers as the primary reference (this text benchmark is not pathological diagnostic gold standard) to evaluate the concordance of text-derived C-TIRADS grades assigned by six large language models (LLMs), with and without FastGPT Retrieval-Augmented Generation (RAG) knowledge base assistance. Our primary endpoint was the concordance between grading and physician consensus—a measure fundamentally distinct from pathological diagnostic accuracy. Only a small subgroup with pathological confirmation was used for a supplementary exploratory analysis of the correlation between model risk stratification and pathological benign or malignant findings.

**Methods:**

Six LLMs were configured using the C-TIRADS criteria. A FastGPT knowledge base was built from 11,489 thyroid ultrasound records collected at Yantai Yuhuangding Hospital between 2022 and 2023. Cases were assigned to manual, LLM, and knowledge-base-augmented LLM groups. C-TIRADS grading concordance with expert ultrasound consensus, and grading speed were assessed. A subgroup of 62 pathologically confirmed nodules was used for supplementary exploratory analysis. AUC, sensitivity, specificity, PPV, NPV and Mantel-Haenszel linear trend χ2 were computed to examine the alignment of model C-TIRADS risk tiers with pathological benign and malignant findings.

**Results:**

Among the standalone LLMs, DeepSeek-V3 achieved the highest numerical grading concordance (81.14%). Following the integration of the RAG knowledge base, overall grading consistency improved significantly (*P* < 0.001), with Doubao-pro-128k demonstrating the highest overall concordance at 89.04%. Concordance increased significantly for C-TIRADS Categories 1 and 3 (*P* = 0.027 and *P* < 0.001, respectively), increased numerically for Category 2 (*P* = 0.596), and decreased numerically for borderline Category 4A (*P* = 0.106). The average concordance across all categories rose significantly from 70.39% to 79.75% (*P* < 0.001). The knowledge-augmented Doubao-pro-128k model exhibited excellent inter-rater agreement (*linear-weighted Kappa>0.8, Bonferroni-corrected P<0.006*). The average single-case grading duration of all 12 AI risk tiering pipelines (6 LLMs with/without knowledge base) was 1.30 s, which was 15 times faster than manual grading (19.25 s). Stratified trend and ROC analyses were performed on 62 thyroid nodules with pathological outcomes. Within this small-sample exploratory pathological subgroup, the Doubao-pro-128k integrated with knowledge base established a significantly ascending malignant risk gradient, achieving an AUC of 0.783 (95%*CI*: 0.635, 0.932) for distinguishing benign from malignant lesions. In comparison, knowledge-enhanced Doubao-pro-128k exhibited a trend toward better matching trend with pathological malignancy distribution and numerically the highest positive Youden index of 0.277 (95%*CI*: 0.071, 0.482).

**Conclusion:**

Doubao-pro-128k integrated with knowledge base accelerated text grading and improved overall sonographer concordance; category-specific concordance increased significantly for C-TIRADS 1 and 3, increased numerically for Category 2, and declined numerically without statistical significance for borderline C-TIRADS 4A, indicating that these ambiguous nodules require further optimization. Exploratory pathological analysis revealed variable model-pathology alignment at the C-TIRADS ≥ 4A cutoff: DeepSeek-V3 integrated with knowledge base produced a negative Youden index, while Doubao-pro-128k integrated with knowledge base showed a tentative trend toward better reflected pathological malignancy gradients.

## 1 Introduction

The incidence of papillary thyroid carcinoma (PTC) is rising globally (1). With the widespread adoption of imaging techniques like ultrasound and rising health awareness, more early-stage PTC and suspicious thyroid nodules are being identified. Ultrasound is currently the primary imaging method for thyroid nodules, offering high sensitivity. Clinical data indicate that the prevalence of thyroid nodules detected via ultrasound approaches 25% in the general population (2). Over 50% of thyroid nodules are benign, yet a 7%-15% risk of malignancy persists (3). Malignant lesions, most commonly PTC, can invade surrounding tissues and organs or produce distant metastases, becoming life-threatening if untreated (4).

Currently, ultrasound is the primary imaging modality for evaluating thyroid nodules. During dynamic scanning, physicians must capture representative static images of the nodules, then record textual descriptions and assign C-TIRADS grades. However, C-TIRADS grading consistency varies substantially across hospitals of different tiers and among physicians with disparate years of clinical experience, frequently resulting in inconsistent nodule risk stratification outcomes. To improve grading consistency for thyroid nodules, and to support clinical decision-making and communication among clinicians, several countries have developed standardized thyroid imaging reporting and data systems.

For instance, the American College of Radiology introduced the Thyroid Imaging Reporting and Data System (TI-RADS) in 2017 (5). In 2020, China introduced the latest version of the Chinese Thyroid Imaging Reporting and Data System (C-TIRADS), which classifies thyroid nodules on ultrasound by evaluating malignancy-associated features such as margins, echogenicity, shape, and microcalcifications (6). For C-TIRADS grading, nodules classified as categories 4A, 4B, 4C, and 5 carry malignant risks of 2-10%, 10-50%, 50-90%, and over 90%, respectively, with PTC being the predominant pathological type in these categories. Compared to other international thyroid ultrasound classification systems, C-TIRADS achieves higher consistency between risk tiers and pathological outcomes, with a larger area under the receiver operating characteristic curve (ROC), which reduces inconsistent grading results among different clinicians and subsequent medical disputes or accidents (7). Large language models (LLMs) are deep learning-based natural language processing (NLP) systems known for their efficient and accurate linguistic capabilities, and they are now widely used in fields such as customer service, media content creation, intelligent tutoring, and financial risk assessment (8–11).Studies have demonstrated that LLMs exhibit exceptional information extraction capabilities, demonstrating significant potential and value in medical domains such as healthcare record analysis and medical literature research (12–14). FastGPT is a knowledge base question-and-answer (Q&A) system based on Retrieval Augmented Generation (RAG) technology, enabling efficient integration and precise retrieval of specialized data.

However, research on the application of LLMs combined with the FastGPT knowledge base to improve C-TIRADS grading consistency and report standardization remains limited. Most existing thyroid AI work centers on image-based nodule risk grouping, while few studies systematically compare the performance of mainstream domestic and foreign LLMs in text-based C-TIRADS grading, and lack verification of RAG knowledge base calibration effect on borderline C-TIRADS 4A nodules (15–17). This study employs multiple LLMs to enhance background knowledge accumulation through integrated with knowledge base learning (18), aiming to identify an accurate and efficient model that improves ultrasonic classification grading consistency, increases report objectivity and standardization. Clinically, C-TIRADS 4A nodules with ambiguous ultrasonic features are the main source of inter-observer inconsistency among sonographers, and whether RAG technology can resolve or partially retain this clinical ambiguity remains unclear (19).

Before stating the research hypothesis, it is essential to clearly distinguish between two evaluation benchmarks to avoid conceptual ambiguity. The primary benchmark for comparison in this study is the C-TIRADS consensus grading, which was independently assigned by senior sonographers based on the same set of de-identified ultrasound text reports used as input for the large language model. This text-based consensus should not be regarded as a clinical gold standard, as it does not satisfy the fundamental methodological requirement that a text-based sonographer consensus benchmark be independent of the test being evaluated—both the human readers and the model relied on identical textual descriptions without access to additional clinical data. In contrast, pathological findings from aspiration or surgical specimens represent the true clinical gold standard. These were used only in a small exploratory subgroup to assess how well the model’s risk categories aligned with actual malignancy rates, and were not employed in calculating the primary concordance outcome for grading.

Research Hypothesis: LLM combined with C-TIRADS guideline-based RAG knowledge base can significantly improve the grading consistency of low-and medium-risk thyroid nodules (C-TIRADS 1-3) by standardizing ultrasonic feature interpretation; however, for borderline C-TIRADS 4A nodules with inherent clinical ambiguity, RAG retrieval of overlapping benign and malignant cases will not improve grading consistency, and may slightly reduce manual matching consistency, while optimizing the monotonic malignant risk gradient of model grading.

We evaluated six mainstream large language models (LLMs) in both standalone and RAG integrated with knowledge base modes. The primary endpoint was the consistency of text-based grading for low- and intermediate-risk thyroid nodule reports (C-TIRADS 1-4A), while a secondary exploratory analysis assessed the alignment between model tier outputs and pathological findings. This pathological alignment metric is distinct from grading consistency and does not measure inter-physician concordance or pathological diagnostic accuracy.

## 2 Materials and methods

### 2.1 Data collection

Case records from 11,489 thyroid ultrasound examinations performed at Yantai Yuhuangding Hospital between January 1, 2022, and December 31, 2023, were used to construct the FastGPT integrated with knowledge base, which supplied background reference materials for six LLMs to carry out text-based C-TIRADS risk tiering of thyroid nodules. A test set comprising 228 thyroid ultrasound reports from examinations conducted at the same hospital in December 2024 was selected to evaluate the grading concordance of these six LLMs on thyroid nodules.

Inclusion criteria for test-set cases: (1) complete ultrasound text reports containing all core morphological features required for C-TIRADS scoring; (2) adult patients aged ≥18 years with a clearly defined primary index nodule; (3) fully de-labeled text without embedded physician grading conclusions.

Exclusion criteria: (1) missing key ultrasonic descriptors for C-TIRADS stratification; (2) duplicate or repeat ultrasound visits; (3) residual embedded C-TIRADS risk labels before text cleaning. The test set included 228 patients (124 females, 104 males; mean age 52.5 ± 16.2 years, range 18–86 years).

Within the full 228-case test set, 62 patients obtained definitive pathological findings via fine-needle aspiration cytology or thyroidectomy specimens, including 12 pathologically confirmed PTC cases and 50 benign nodules. This pathological subgroup served as **Supplementary Material** to evaluate the ordered malignant risk gradient and alignment indicators: AUC, sensitivity, specificity, positive predictive value (PPV), negative predictive value (NPV), trend chi-square, while the primary text-based sonographer consensus benchmark for all concordance analyses remained text-only expert C-TIRADS consensus grading, and pathological findings were not used to define the primary study endpoint of grading consistency.

To prevent label leakage, before structured conversion and LLM input, all original ultrasound free-text reports underwent standardized cleaning: all preset C-TIRADS classification labels, physician-assigned risk tier notes, malignant tendency assessments and tiering conclusions written by sonographers were completely removed from the text. Only objective ultrasonic feature descriptions (nodule size, boundary, echogenicity, calcification, aspect ratio, blood flow signal, location) were retained as input materials to avoid the model directly extracting existing grading labels rather than conducting independent feature reasoning. For both the LLM and knowledge-base-augmented LLM groups, only textual reports were analyzed. Each report was processed with one index nodule per input, defined as the largest or most suspicious nodule for potential malignancy. Nodule location was coded as left, right, or isthmus and as upper, middle, or lower pole. For patients with multiple nodules, only the index nodule was included in the analysis.

### 2.2 Methodology

Six LLMs were evaluated. DeepSeek-V3, developed by the Chinese artificial intelligence (AI) company DeepSeek, is a Mixture of Experts model that achieves top-tier concordance in mathematical reasoning, code generation, and multilingual processing (20). ChatGPT-4o-Latest, developed by OpenAI, exhibits robust contextual comprehension and continuously improves through feedback and usage, allowing it to provide responses that better align with user needs while demonstrating strong adaptability and flexibility. GPT-3.5-Turbo is an enhanced extension of the GPT-3.5 language model, trained on extensive datasets using advanced deep learning techniques; it possesses strong language comprehension and generation capabilities, enabling high-quality responses based on input text and handling substantial data processing tasks (21). Doubao-pro-128k (22), launched by ByteDance, demonstrates robust Chinese language processing capabilities and holds significant advantages in handling large volumes of text. GLM-4-plus (23), developed by Zhipu AI, can more accurately understand user input to provide more relevant and precise responses, while achieving excellent results in long-text processing. Qwen-plus (24), a leading LLM on the Alibaba Cloud platform, effectively handles complex tasks and demonstrates excellent concordance in logical reasoning, problem analysis, and complex scenario comprehension, with reasoning abilities comparable to GPT-4 and Claude-3.5-Sonnet.

#### 2.2.1 Structured text conversion

Transforming free-text thyroid ultrasound reports into structured data using the General Language Model-4 (GLM-4) allows extraction of report serial numbers, nodule location, size, echogenicity features, morphology, aspect ratio, boundary, internal echo uniformity, calcification status, and blood flow signal—key features for PTC risk stratification. Further full-set validation based on the corrected test-set confusion matrix confirmed an overall structured grading concordance of 96.49% (220/228) between GLM-4-plus features and expert consensus grading. Further full-set validation of the structured grading performance based on the complete test-set confusion matrix is presented in the **Supplementary Materials S2**.

#### 2.2.2 Knowledge base construction

A total of 11,489 thyroid ultrasound examination records were imported into the knowledge base. During data preprocessing, duplicate entries, incomplete reports, and records without definitive C-TIRADS labels were discarded. Raw free-text ultrasound narratives were standardized by stripping special symbols, redundant whitespace, and extraneous non-clinical text. Semantic vectorization was performed using the Shaw Chinese Vectorization Model, and GLM-4 was utilized for text parsing and structured output generation, yielding standardized reference materials dedicated to text-based C-TIRADS risk tiering. The entire system was built in strict accordance with the 2020 Chinese C-TIRADS guidelines.

All model-related orchestration components (including FastGPT knowledge-base service and the self-hosted One-API scheduling gateway) ran inside independent Docker containers managed by Docker Compose within the hospital internal intranet in December 2024. Two distinct model operation modes were implemented for the evaluated large-language models.

Open-source models (DeepSeek-V3, GLM-4-plus, Qwen-plus) were fully locally deployed with complete model weight files on dedicated host servers. FastGPT accessed these locally-resident models via the intranet-only One-API gateway endpoint `http://localhost:3001/`.

Closed-source commercial models (Doubao-pro-128k, GPT-3.5-Turbo, ChatGPT-4o-Latest) were not locally deployed. Instead, they were remotely invoked via their respective vendor cloud Application Programming Interfaces (APIs). The self-hosted One-API gateway acted as a local-intranet-resident proxy scheduler: FastGPT submits requests to the local One-API gateway (`http://localhost:3001/`); the gateway receives fully de-identified ultrasound text, then forwards these anonymized payloads through a controlled, managed outbound network pathway to the corresponding vendor public cloud API endpoints. Vendor API access credentials are securely stored only within the One-API gateway service and are not accessible to other intranet services. Only de-identified clinical text without any personal health identifiers traversed outside the hospital intranet toward third-party vendor servers; raw identifiable patient records never exited the hospital network.

Fixed decoding hyperparameters were applied across all models: temperature=0.1, nucleus sampling threshold=0.9, maximum output tokens=512, and a static random seed of 42. Text segmentation was configured with a chunk size of 512 tokens and a 50-token overlap. Complete prompt templates, data preprocessing workflows, structured report templates, input-output samples, retrieval parameters, vectorization model specifications, model version information (all deployed December 2024), and error handling protocols are archived in **Supplementary Material S1** to support full study reproducibility.

Uniform grading prompts were developed for the six LLMs (DeepSeek-V3, ChatGPT-4o-Latest, GPT-3.5-Turbo, Doubao-pro-128k, GLM-4-plus, and Qwen-plus) following official C-TIRADS criteria. Standardized definitions of key suspicious sonographic markers (marked hypoechogenicity, microcalcification, irregular margins, aspect ratio > 1, and tissue invasion) were embedded into each model’s system prompt.

A hybrid retrieval scheme combining vector semantic search and full-text matching was adopted to boost retrieval stability. We set a relevance score decision threshold of 0.9 and leveraged the Reciprocal Rank Fusion (RRF) algorithm to reorder retrieved references, with a maximum citation limit of 1,500 entries. This configuration guaranteed that retrieved clinical context matched standardized C-TIRADS grading logic, further improving consistency and reliability of model risk tiering.

### 2.3 Group grading setup

According to different analysis modalities, enrolled cases were assigned into three groups: the manual analysis group, the LLM group, and the knowledge-base-augmented LLM group. To ensure a fair comparison, the text-based sonographer consensus benchmark (expert consensus C-TIRADS grade) was derived from the same cleaned text feature reports used as LLM input. Unified information source setting for fair comparison: All three senior sonographers in the manual analysis group only viewed the same cleaned text feature reports as the LLMs; ultrasound images, thyroid function test results, fine-needle aspiration cytology (FNAC) cytology and surgical pathological data were completely masked during the consensus grading session, to ensure human experts and LLMs obtain identical input information. The final consensus grading was only used as the text-based C-TIRADS text-based sonographer consensus benchmark.

In the manual analysis group, three sonographers with over 15 years of clinical experience independently graded thyroid nodules in the test set following the C-TIRADS criteria using only the cleaned text feature descriptions. Meanwhile, the average single-case grading duration was recorded. In the LLM group, the test dataset was imported in batches into six mainstream large language models, including DeepSeek-V3, ChatGPT-4o-Latest, GPT-3.5-Turbo, Doubao-pro-128k, GLM-4-plus, and Qwen-plus. Standardized grading outputs were generated by executing standardized thyroid ultrasound classification prompts. All results were unified into a fixed format: report id, nodule location, and thyroid nodule classification (**Figure 1**).

**Figure 1:**
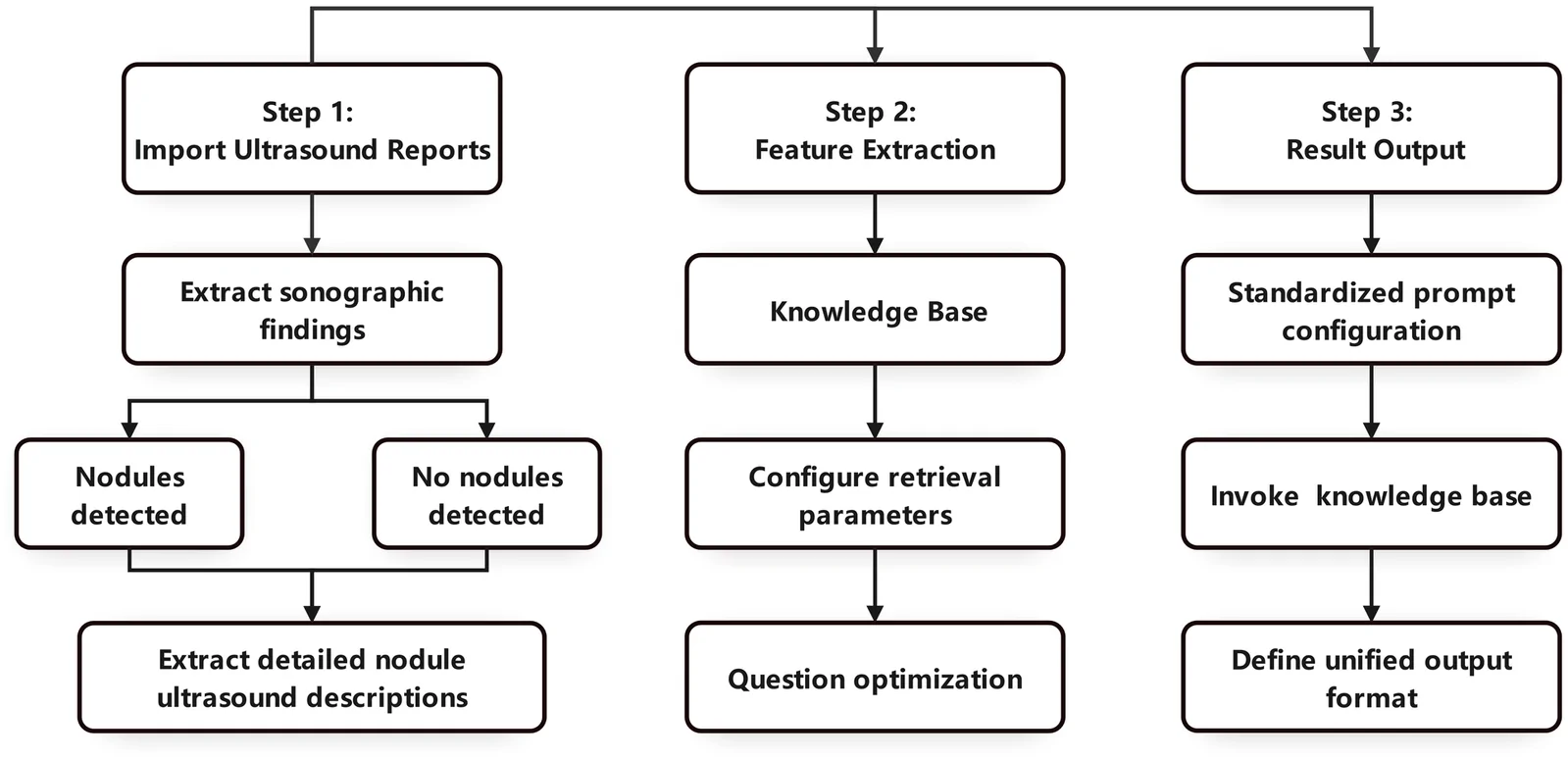
LLM-based C-TIRADS grading for thyroid nodule ultrasound descriptions.

In the knowledge-base-augmented LLM Group, the test datasets were imported in batches into six LLMs integrated with knowledge base. Using RAG technology, the most relevant reference case fragments supporting thyroid nodule C-TIRADS grading were selected as background context. Following standardized C-TIRADS grading guidelines, the models completed standardized risk tiering and output thyroid nodule grade results (**Figure 2**).

**Figure 2:**
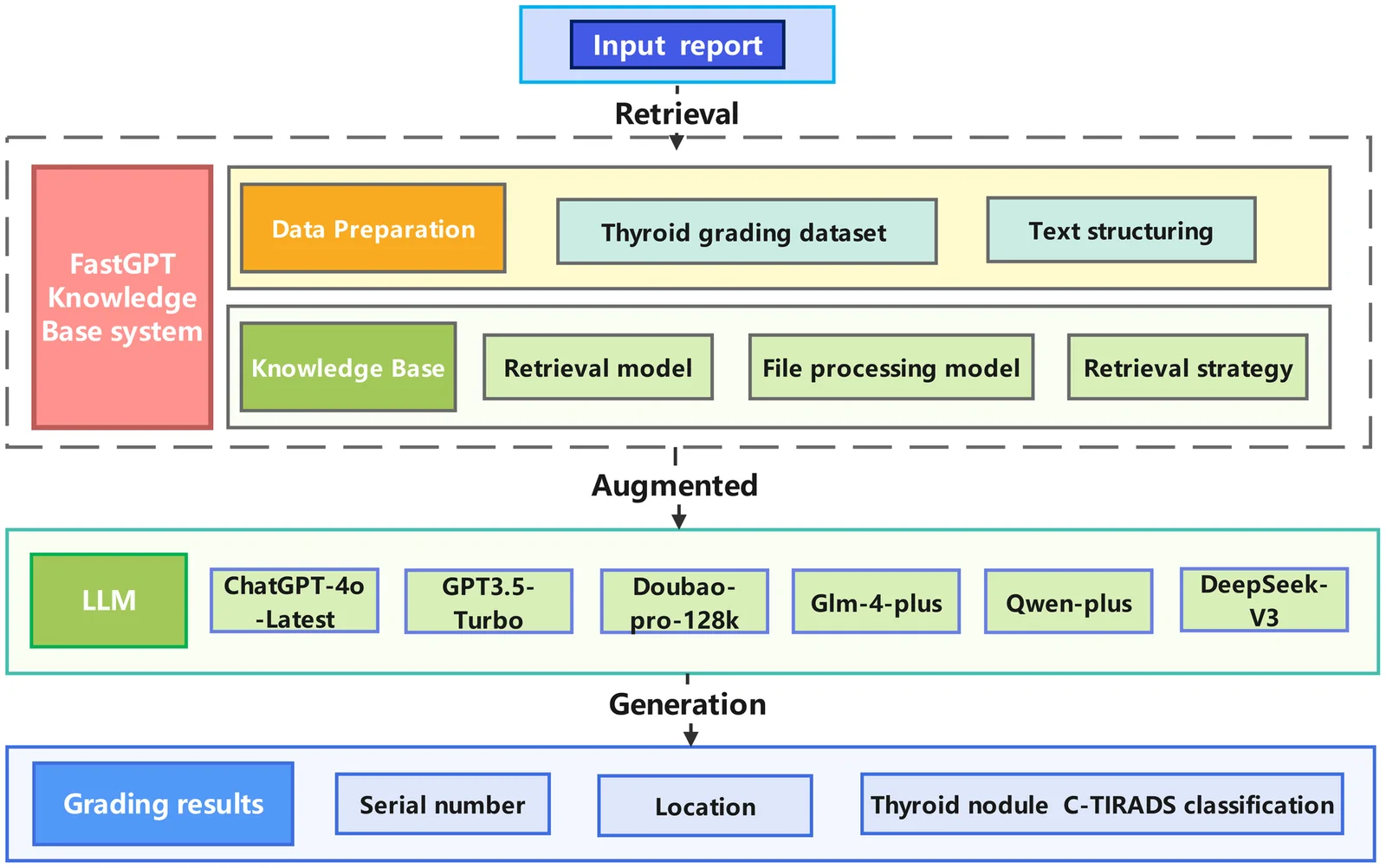
Workflow of thyroid nodule classification grading using knowledge-base-augmented LLM.

### 2.4 Result compilation

Following analysis by the LLM, the output results were formatted as “Report Number, Location, Thyroid Nodule Classification”. The grading concordance of thyroid nodule classification and the average single-case grading duration of each group were statistically analyzed to evaluate C-TIRADS grading concordance and their consistency with manual analysis results (**Figure 3**). The figures presented in this report are randomly generated sequences of numbers, produced independently and bearing no correlation to any actual patient medical records.

**Figure 3:**
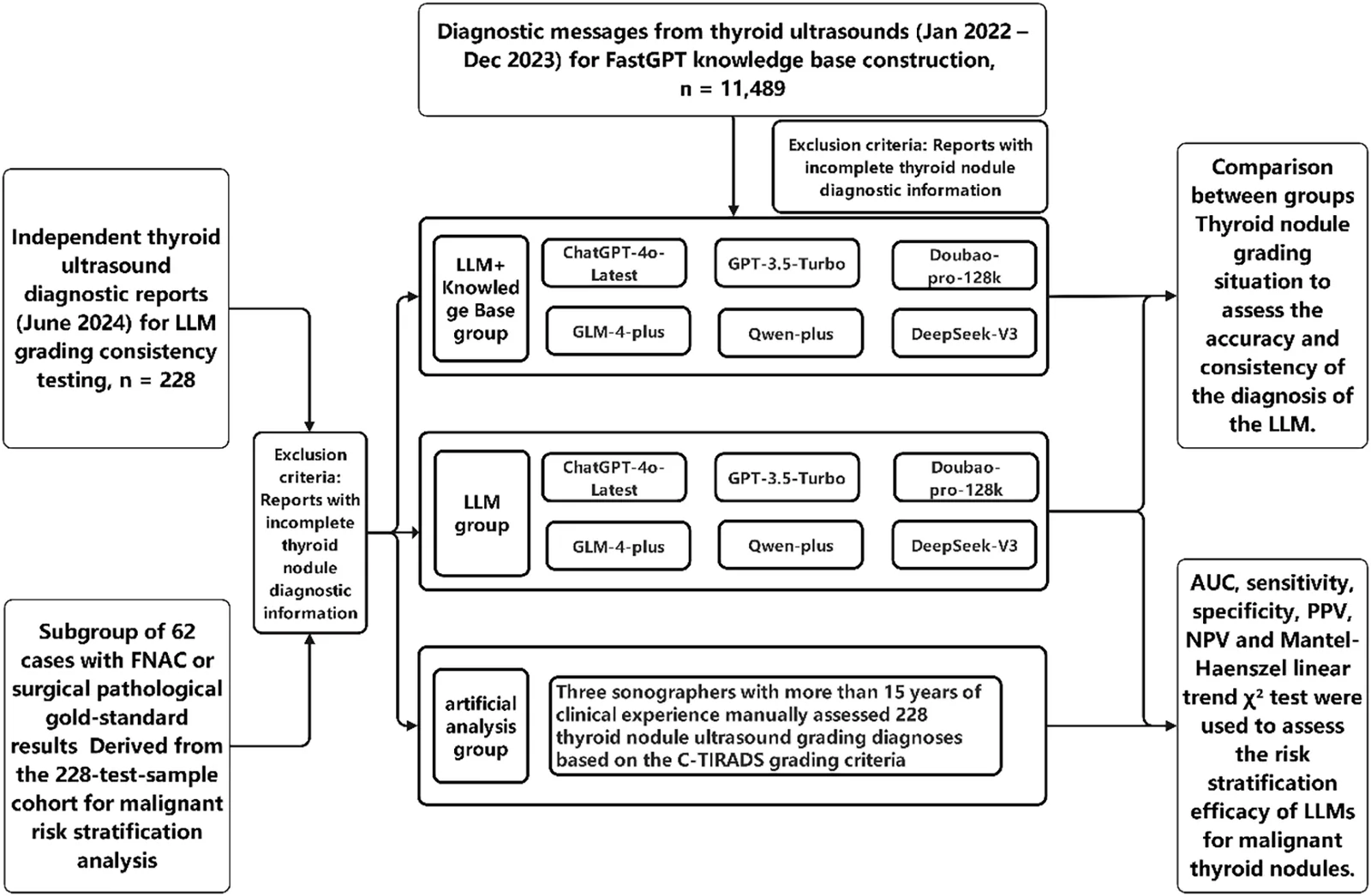
Flowchart of the study on LLM-RAG framework for improving C-TIRADS grading consistency of thyroid nodules.

### 2.5 Ethics

Formal ethical approval was obtained from the Institutional Review Board of Affiliated Yantai Yuhuangding Hospital of Qingdao University, approval number: 2024-608. The ethics approval document is provided in **Supplementary Material S3**. The IRB issued a formal waiver of written informed consent, as this study is a retrospective secondary analysis of fully de-identified clinical imaging text data without any clinical intervention to patients. All ultrasound reports underwent strict desensitization: all patient names, ID numbers, contact information, admission dates and other identifiable personal information were permanently deleted, only anonymous case serial numbers were retained for analysis. The FastGPT knowledge-base service and locally-deployed open-source LLMs resided entirely on hospital local servers without external network access. For closed-source commercial models invoked via vendor APIs: only fully de-identified, patient-identifier-free text snippets were transmitted to external vendor cloud endpoints, whereas raw identifiable patient medical records never left the hospital intranet and were never sent to external commercial cloud APIs. The whole research fully complies with China’s Personal Information Protection Law and medical health data cross-border transfer management regulations, and no cross-border transmission of patient data occurred.

### 2.6 Statistical analysis

Data entry and organization were performed using Microsoft Office Excel Professional Plus 2021. Statistical analyses were conducted using SPSS 26.0 and GraphPad Prism 9.5.0 (730) software.

The 228-case test dataset was markedly imbalanced in the high-risk C-TIRADS groups, with 11 C-TIRADS 4B nodules, no C-TIRADS 4C nodules, and one C-TIRADS 5 nodule. Two separate analytical schemes were used. For the primary linear-weighted Kappa consistency analysis (**Tables 1**, **2**), all C-TIRADS tiers 1–5 were included because ordinal weighted Kappa tolerates uneven sample distribution across categories. For the supplementary stratified McNemar tests comparing standalone LLMs and knowledge base-enhanced LLMs (**Table 3**), C-TIRADS 4B-5 were excluded from stratified significance testing because the extremely small sample sizes led to unstable chi-square results. This exclusion affects only the tier-specific pairwise comparisons in **Table 3**, whose stratified interpretations are limited to C-TIRADS 1-4A, whereas all core Kappa-based consistency outcomes cover the complete C-TIRADS.

**Table 1:** Comparison of consistency in thyroid classification results for standalone LLM group.

Group	Kappa Value	95%CI	Original P-value	Corrected P-value (Bonferroni)
ChatGPT-4o-Latest	0.433	(0.214, 0.652)	0.003	0.018
GPT-3.5-Turbo	0.654	(0.583, 0.726)	*P*<0.001	*P<0.006*
DeepSeek-V3	0.834	(0.781, 0.886)	*P*<0.001	*P<0.006*
Doubao-pro-128k	0.815	(0.765, 0.865)	*P*<0.001	*P<0.006*
GLM-4-plus	0.645	(0.575, 0.715)	*P*<0.001	*P<0.006*
Qwen-plus	0.739	(0.683, 0.795)	*P*<0.001	*P<0.006*

Linear weighted Kappa with 95% confidence intervals was used to evaluate text-based C-TIRADS grading concordance between standalone LLMs and senior sonographer consensus. Six independent comparisons were conducted within this table, and Bonferroni correction was applied to control familywise Type I error. The adjusted significance threshold was α’ = 0.05/6 ≈ 0.00833. Kappa interpretation criteria: <0.4 poor agreement; 0.4–0.6 moderate agreement; 0.6–0.8 substantial agreement; ≥0.8 excellent agreement.

**Table 2:** Comparison of consistency in thyroid classification results for LLM Integrated knowledge base group.

Group	Kappa Value	95%CI	Original P-value	Corrected P-value (Bonferroni)
ChatGPT-4o-Latest + KB	0.816	(0.762, 0.869)	*P*<0.001	*P<0.006*
GPT-3.5-Turbo + KB	0.719	(0.655, 0.783)	*P*<0.001	*P<0.006*
DeepSeek-V3 + KB	0.845	(0.790, 0.900)	*P*<0.001	*P<0.006*
Doubao-pro-128k + KB	0.893	(0.849, 0.937)	*P*<0.001	*P<0.006*
GLM-4-plus + KB	0.829	(0.778, 0.879)	*P*<0.001	*P<0.006*
Qwen-plus + KB	0.786	(0.733, 0.839)	*P*<0.001	*P<0.006*

Linear weighted Kappa with 95% confidence intervals was used to evaluate text-based C-TIRADS grading concordance between LLM+KB schemes and senior sonographer consensus. Six independent comparisons were conducted within this table, and Bonferroni correction was applied to control familywise Type I error. The adjusted significance threshold was α’ = 0.05/6 ≈ 0.00833. Kappa interpretation criteria: <0.4 poor agreement; 0.4–0.6 moderate agreement; 0.6–0.8 substantial agreement; ≥0.8 excellent agreement.

**Table 3:** Comparison of grading concordance between standalone and knowledge-base-augmented LLMs, stratified by C-TIRADS category.

C-TI-RADS	Status	LLM Group (%)	LLM with Integrated Knowledge Base Group (%)	(McNemar test)	(McNemar test)
				χ^2^	P-value
Category 1	Consistent	266 (96.38)	274 (99.28)	4.900	*0.027*
	Inconsistent	10 (3.62)	2 (0.72)		
Category 2	Consistent	93 (73.81)	97 (76.98)	0.281	*0.596*
	Inconsistent	33 (26.19)	29 (23.02)		
Category 3	Consistent	396 (66.67)	496 (83.50)	48.044	*P<0.001*
	Inconsistent	198 (33.33)	98 (16.50)		
Category 4A	Consistent	189 (63.00)	169 (56.33)	2.616	*0.106*
	Inconsistent	111 (37.00)	131 (43.67)		
Total (all 228 cases)	Consistent	963 (70.39)	1091 (79.75)	37.862	*P*<0.001
	Inconsistent	405 (29.61)	277 (20.25)		

Each row represents paired concordance outcomes for the same thyroid nodules evaluated by six standalone LLMs and their corresponding KB-integrated versions. Continuity-corrected McNemar’s tests were calculated using discordant paired observations. C-TIRADS 4B, 4C, and 5 were excluded from category-specific significance testing because of severe sample sparsity. The Total row includes all 228 nodules across the six paired model comparisons.

Distinct sample inclusion criteria were adopted for the secondary exploratory subgroup analysis based on pathological findings. We enrolled 62 nodules with definitive fine-needle aspiration cytology or surgical pathological findings. All C-TIRADS 4B and 5 samples were retained when calculating Mantel-Haenszel linear trend χ2 statistics and ROC alignment indices.

Linear-weighted Kappa coefficients with 95% confidence intervals were used to quantify inter-group agreement of C-TIRADS risk tiering. The linear-weighted Kappa coefficient was calculated as: $\kappa_{w}=\frac{p_{o}\left(w\right)-p_{e}\left(w\right)}{1-p_{e}\left(w\right)}$, where $p_{o}\left(w\right)=\sum_{i}\sum_{j}w_{ij}p_{ij}$ is the weighted observed agreement, $p_{e}\left(w\right)=\sum_{i}\sum_{j}w_{ij}p_{i+}p_{+j}$ is the weighted expected agreement under independence, and the linear weights are defined as $w_{ij}=1-\frac{\left|i-j\right|}{k-1}$, with *i*, *j* indexing the C-TIRADS categories and *k* denoting the total number of categories. Two-sided statistical significance was set at P<0.05 (25). For the paired base-versus-knowledge-base comparisons in **Table 4**, continuity-corrected McNemar tests were used and raw, unadjusted P values are reported; these model-specific comparisons were exploratory, and no multiplicity correction was applied. The stratified comparisons in **Table 3** likewise report unadjusted exploratory P values. For the Kappa analyses, Bonferroni correction was applied separately to the six comparisons in **Table 1** and the six comparisons in **Table 2**, yielding α’=0.05/6≈0.00833 within each table.

**Table 4:** Comparison of thyroid nodule classification between large language model group and AI large language model integrated knowledge base group.

Model	Consistent VS Consistent	Consistent VS Inconsistent	Inconsistent VS Consistent	Inconsistent VS Inconsistent	(McNemar test) χ2	(McNemar test) P-value
ChatGPT-4o-Latest VS ChatGPT-4o-Latest+ KB	137	18	44	29	10.081	0.001
GPT-3.5-Turbo VS GPT-3.5-Turbo + KB	101	48	55	24	0.350	0.554
Doubao-pro-128k VS Doubao-pro-128k + KB	159	18	44	7	10.081	0.001
GLM-4-plus VS GLM-4-plus + KB	127	16	59	26	23.520	*P*<0.001
Qwen-plus VS Qwen-plus + KB	124	30	53	21	5.831	0.016
DeepSeek-V3 VS DeepSeek-V3 + KB	166	19	22	21	0.098	0.755

Each row represents paired 2×2 contingency results for identical thyroid nodules evaluated by the base LLM and its KB-integrated version. Consistent vs Consistent = nodules with consistent classification in both models; Consistent vs Inconsistent = nodules consistent only in the base LLM; Inconsistent vs Consistent = nodules consistent only in the KB-integrated LLM; Inconsistent vs Inconsistent = nodules inconsistent in both models. Continuity-corrected McNemar’s test was performed based on discordant paired cases to compare differences in classification consistency between the paired models.

For the supplementary exploratory subgroup of 62 pathologically verified nodules (12 PTC, 50 benign lesions), we carried out auxiliary analyses to evaluate the correspondence between model-generated risk tiers and pathological outcomes. ROC curves were generated and corresponding AUC values computed to measure the matching degree between each model’s C-TIRADS stratification and pathological benign/malignant labels. AUCs, standard errors, and asymptotic 95% CIs were estimated using a nonparametric empirical ROC approach. Pairwise comparisons of ROC AUC between grading pipelines were performed using DeLong’s test for correlated ROC curves, with Bonferroni correction applied across the six comparisons reported in **Table 5**. Alignment metrics including sensitivity, specificity, PPV, NPV and Youden index were calculated under the cutoff C-TIRADS ≥ 4A, which served as a decision threshold to flag samples with elevated malignant risk. Two-sided 95% confidence intervals for binomial proportions, including sensitivity, specificity, positive predictive value (PPV), and negative predictive value (NPV), were calculated using the Clopper-Pearson exact method. The 95% confidence interval for the Youden index (J=sensitivity+specificity−1) was calculated using the delta method. NPV was considered mathematically undefined and reported as “—” exclusively when TN + FN = 0 (i.e., no negative predictions); when TN = 0 and FN > 0, NPV = 0.

**Table 5:** Pairwise DeLong Test Results (Reference: Doubao-pro-128k + KB, AUC = 0.783).

Comparison	AUC diff.	*95% CI*	*Z*	*P (raw)*	*P (Bonf.)*
vs Manual expert assessment	0.038	(-0.041, 0.116)	0.937	0.349	1
vs DeepSeek-V3 + KB	0.186	(0.084, 0.287)	3.590	<0.001	0.002 *
vs DeepSeek-V3	0.089	(-0.057, 0.235)	1.196	0.232	1
vs Doubao-pro-128k	0.300	(0.183, 0.417)	5.035	<0.001	<0.001 *
vs ChatGPT-4o-Latest + KB	0.214	(0.086, 0.343)	3.271	0.001	0.006 *
vs GLM-4-plus + KB	0.148	(0.034, 0.263)	2.540	0.011	0.066

*Raw *P* < 0.0083, statistically significant after Bonferroni correction for six comparisons.

The Mantel-Haenszel linear trend *χ2* test was utilized to verify whether the proportion of pathologically confirmed malignant lesions increased sequentially as model-assigned C-TIRADS risk tiers rose.

## 3 Results

### 3.1 Manual analysis group

Ultrasound-based classification of 228 thyroid nodules by human evaluators identified 46 nodules as C-TIRADS Category 1, 21 as Category 2, 99 as Category 3, 50 as Category 4A, 11 as Category 4B, none as Category 4C, and one as Category 5. Inter-observer agreement among the three sonographers was assessed with linear-weighted kappa, yielding *Kappa* = 0.87 (*95%CI:*0.820-0.910, *P* < 0.001), which indicates excellent consistency.

### 3.2 Comparison between the LLM group and manual analysis group

Among the standalone LLMs, DeepSeek-V3 achieved the highest overall concordance rate (81.14%, **Table 6**). When stratified by risk category, notable variability emerged across models for C-TIRADS 3 lesions, with DeepSeek-V3 showing the highest numerical concordance and Qwen-plus the lowest. For borderline C-TIRADS 4A nodules, Doubao-pro-128k and Qwen-plus demonstrated relatively higher agreement rates, while GLM-4-plus performed the weakest numerically. Given the extremely small sample sizes in the high-risk categories (n=11 for 4B and n=1 for 5), the concordance values for these subgroups should be interpreted as anecdotal only (**Table 6**). **Table 6** presents the baseline concordance percentages for each LLM in the absence of a knowledge base. These values serve as the reference for quantifying the changes in concordance following RAG integration, as discussed in Section 3.3.

**Table 6:** Comparison of thyroid nodule grading concordance among various models in the standalone LLM group [Cases (%)].

C-TIRADS	Manual expert assessment	ChatGPT-4o-Latest (%)	GPT-3.5-Turbo (%)	Doubao-pro-128k (%)	GLM-4-plus (%)	Qwen-plus (%)	DeepSeek-V3 (%)	Average concordance rate (%)
Category 1	46	44 (95.65)	40 (86.96)	46 (100.00)	44 (95.65)	46 (100.00)	46 (100.00)	96.38
Category 2	21	18 (85.71)	17 (80.95)	18 (85.71)	6 (28.57)	16 (76.19)	18 (85.71)	73.81
Category 3	99	65 (65.66)	65 (65.66)	65 (65.66)	67 (67.68)	47 (47.47)	87(87.88)	66.67
Category 4A	50	23 (46.00)	27 (54.00)	47 (94.00)	21 (42.00)	45 (90.00)	26(52.00)	63.00
Category 4B	11	5 (45.45)	0 (0.00)	0 (0.00)	5 (45.45)	0 (0.00)	7(63.64)	25.76
Category 5	1	0 (0.00)	0 (0.00)	1 (100.00)	0 (0.00)	0 (0.00)	1(100.00)	33.33
Total	228	155 (67.98)	149 (65.35)	177 (77.63)	143 (62.72)	154 (67.54)	185(81.14)	70.39

### 3.3 Comparison between the knowledge-base-augmented LLM group and the manual analysis group

Paired continuity-corrected McNemar tests showed nominally significant improvements in four of the six LLMs based on raw, unadjusted P values: Doubao-pro-128k rose from 77.63% (standalone) to 89.04% (with knowledge base), an absolute increase of 11.41 percentage points (*P* = 0.001); ChatGPT-4o-Latest improved from 67.98% to 79.39%, an 11.41 percentage-point improvement (*P* = 0.001); GLM-4-plus rose from 62.72% to 81.58%, an 18.86 percentage-point improvement (*P* < 0.001); and Qwen-plus improved from 67.54% to 77.63%, a 10.09 percentage-point improvement (*P* = 0.016). In contrast, GPT-3.5-Turbo (*P* = 0.554) and DeepSeek-V3 (*P* = 0.755) exhibited no statistically significant improvement in consistency following RAG enhancement. Their concordance shifts were minimal: GPT-3.5-Turbo improved by just 3.07 percentage points (from 65.35% to 68.42%), while DeepSeek-V3 saw a similarly modest gain of 1.32 percentage points (from 81.14% to 82.46%). Descriptively, Doubao-pro-128k integrated with the knowledge base had the highest overall concordance among all 12 model pipelines, at 89.04%.

Stratified analyses by C-TIRADS risk category further revealed distinct changes in consistency: concordance increased significantly for Categories 1 and 3 (*P* = 0.027 and *P* < 0.001, respectively), whereas the increase for Category 2 (*P* = 0.596) and the reduction for Category 4A (*P* = 0.106) were not statistically significant. Across all six LLMs, the average concordance for C-TIRADS 1, 2, and 3 nodules rose by 2.90, 3.17, and 16.83 percentage points, respectively. In contrast, concordance for borderline C-TIRADS 4A nodules declined from 63.00% to 56.33%, representing an absolute decrease of 6.67 percentage points. Categories 4B, 4C, and 5 were excluded from category-specific statistical testing because sparse cell counts precluded stable estimates; the total comparison retained all categories (**Tables 3**, **4**, **6**, **7**).

**Table 7:** Comparison of thyroid nodule grading concordance across different LLMs integrated with knowledge base.

C-TIRADS	Manual expert assessment	ChatGPT-4o-Latest + KB (%)	GPT-3.5-Turbo + KB (%)	Doubao-pro-128k + KB (%)	GLM-4-plus + KB (%)	Qwen-plus + KB (%)	DeepSeek-V3 + KB (%)	Average concordance rate (%)
Category 1	46	46 (100.00)	45 (97.83)	46 (100.00)	46 (100.00)	45 (97.83)	46(100.00)	99.28
Category 2	21	18 (85.71)	4 (19.05)	21 (100.00)	17 (80.95)	20 (95.24)	17(80.95)	76.98
Category 3	99	82 (82.83)	60 (60.61)	93 (93.94)	98 (98.99)	83 (83.84)	80(80.81)	83.50
Category 4A	50	27 (54.00)	41 (82.00)	31 (62.00)	15 (30.00)	22 (44.00)	33(66.00)	56.33
Category 4B	11	7 (63.64)	5 (45.45)	11 (100.00)	9 (81.82)	6 (54.55)	11(100.00)	74.24
Category 5	1	1 (100.00)	1 (100.00)	1 (100.00)	1 (100.00)	1 (100.00)	1(100.00)	100.00
Total	228	181 (79.39)	156 (68.42)	203 (89.04)	186 (81.58)	177 (77.63)	188(82.46)	79.75

### 3.4 Single-case grading duration for thyroid nodule classification

The single-case grading duration of three senior physicians was recorded individually, yielding an average of 19.25 seconds. Among LLMs, GPT-3.5-Turbo achieved the shortest average single-case grading duration at 0.28 seconds, which is approximately 69 times faster than manual grading. Doubao-pro-128k required 1.06 seconds, approximately 18 times faster than manual grading. Integrating a knowledge base increased the single-case grading duration of LLMs compared to using the models alone. The average single-case grading duration across 12 conditions was 1.30 seconds, which is 15 times faster than manual analysis (**Table 8**).

**Table 8:** Comparison of time usage for thyroid nodule C-TIRADS grading.

Group	Total time (s)	Average diagnostic time (s)
Manual expert assessment	4389.00	19.25
ChatGPT-4o-Latest	107.47	0.47
GPT-3.5-Turbo	63.48	0.28
DeepSeek-V3	243.13	1.07
Doubao-pro-128k	241.82	1.06
GLM-4-plus	201.60	0.88
Qwen-plus	238.22	1.04
ChatGPT-4o-Latest + KB	251.60	1.10
GPT-3.5-Turbo + KB	314.00	1.38
DeepSeek-V3+ KB	500.91	2.20
Doubao-pro-128k + KB	387.78	1.70
GLM-4-plus + KB	225.83	0.99
Qwen-plus + KB	792.46	3.48

### 3.5 Consistency tests between LLMs and manual analysis results

Using grading outcomes from the manual expert consensus group as the text-based sonographer consensus benchmark, linear-weighted Kappa consistency tests were performed to evaluate C-TIRADS grading agreement between each LLM configuration and manual assessment. The unadjusted P-value of every pairwise comparison was less than 0.05, preliminarily indicating statistically significant grading consistency between each model and sonographers. Among all groups, Doubao-pro-128k integrated with knowledge base, DeepSeek-V3 integrated with knowledge base, standalone DeepSeek-V3, GLM-4-plus integrated with knowledge base, ChatGPT-4o-Latest integrated with knowledge base and standalone Doubao-pro-128k achieved Kappa values greater than 0.8 with 95% confidence intervals entirely above zero, which represented excellent agreement with text-only manual grading. The Kappa value of standalone ChatGPT-4o-Latest ranged from 0.4 to 0.6, corresponding to moderate agreement.

To reduce excessive statistical punishment from 12 parallel comparisons, all consistency analyses were divided into two independent subsets, which were presented in **Table 1** (six standalone LLM groups) and **Table 2** (six knowledge-base-augmented LLMs combined groups) respectively. Bonferroni correction was performed separately for each table to control the familywise Type I error rate, and the adjusted significance decision threshold was set to α’=0.05/6 ≈ 0.00833 for both **Table 1** and **Table 2**. After Bonferroni correction within each table, all six standalone LLMs in **Table 1** and all six knowledge-base-augmented LLMs in **Table 2** showed statistically significant agreement with expert consensus (adjusted P < 0.05) Detailed Kappa values, 95% confidence intervals, original P-values and corrected P-values are listed in **Table 1** and **Table 2**.

### 3.6 Exploratory analysis of malignant risk gradient trend based on pathological subgroup

According to the research findings on consistency testing between outputs from large language models and manual analysis, the groups with a kappa value less than 0.8 are: Qwen-plus, GLM-4-plus, GPT-3.5-Turbo, ChatGPT-4o-Latest, GPT-3.5-Turbo integrated with knowledge base, Qwen-plus integrated with knowledge base. There are 62 cases of thyroid nodules with pathological findings, including 12 cases positive for PTC and 50 benign nodules. The Mantel-Haenszel linear trend chi-square test was adopted to compare the positive detection rates of PTC across different C-TIRADS classifications of thyroid nodules under various evaluation methods. For manual grading, the proportion of pathologically confirmed PTC increased significantly across ascending C-TIRADS risk tiers (χ^2^ = 14.812, *P* < 0.001). Native DeepSeek-V3 also showed a statistically significant upward trend (*χ2* = 7.805, *P* = 0.005), although severe risk underestimation occurred in C-TIRADS 4A. After knowledge-base integration, DeepSeek-V3 did not show a significant graded risk trend (*χ2* = 1.570, *P* = 0.210). Knowledge-enhanced Doubao-pro-128k showed a statistically significant monotonic increase in malignant risk (*χ2* = 13.134, *P* < 0.001). No statistically significant increasing trend was detected for ChatGPT-4o-Latest with knowledge base (*χ2* = 0.583, *P* = 0.445), while GLM-4-plus with knowledge base showed a marginally non-significant trend (*χ2* = 3.579, *P* = 0.059; **Table 9**).

**Table 9:** Comparison of the malignant pathological proportion by different LLMs.

Group	Is the pathology result positive:	C-TIRADS 3 = 1	C-TIRADS 4A=2	C-TIRADS 4B=3	C-TIRADS 4C=4	C-TIRADS 5 = 5	*Mantel-Haenszel χ^2^*	*P-value*
Manual expert assessment(%)	NO	0(0.00)	45(90.0)	5(45.45)	0(0.00)	0(0.00)	14.812	*P*<0.001
	YES	0(0.00)	5(10.00)	6(54.55)	0(0.00)	1(100.00)		
DeepSeek-V3 + KB(%)	NO	1(33.33)	35(92.11)	12(66.67)	2(100.00)	0(0.00)	1.570	0.210
	YES	2(66.67)	3(7.89)	6(33.33)	0(0.00)	1(100.00)		
DeepSeek-V3(%)	NO	17(80.95)	28(100.00)	5(41.67)	0(0.00)	0(0.00)	7.805	0.005
	YES	4(19.05)	0(0.00)	7(58.33)	0(0.00)	1(100.00)		
Doubao-pro-128k+ KB(%)	NO	18(94.74)	27(87.10)	5(45.45)	0(0.00)	0(0.00)	13.134	*P*<0.001
	YES	1(5.26)	4(12.90)	6(54.55)	0(0.00)	1(100.00)		
Doubao-pro-128k(%)	NO	0(0.00)	48(82.76)	2(100.00)	0(0.00)	0(0.00)	0.807	0.369
	YES	1(100.00)	10(17.24)	0(0.00)	0(0.00)	1(100.00)		
ChatGPT-4o-Latest + KB(%)	NO	13(76.47)	26(92.86)	8(61.54)	0(0.00)	3(75.00)	0.583	0.445
	YES	4(23.53)	2(7.14)	5(38.46)	0(0.00)	1(25.00)		
GLM-4-plus+ KB(%)	NO	22(84.62)	14(93.33)	14(70.00)	0(0.00)	0(0.00)	3.579	0.059
	YES	4(15.38)	1(6.67)	6(30.00)	0(0.00)	1(100.00)		

Data were analyzed by Mantel-Haenszel linear trend χ² test; partial cells with expected count <5.

### 3.7 Exploratory ROC analysis of alignment between model risk tiers and pathological findings (pathological subgroup)

ROC curves were constructed to compare pathological risk stratification performance between manual evaluation and all six LLM grading pipelines (**Figure 4**). In this small exploratory pathological subgroup, the curve corresponding to knowledge-enhanced Doubao-pro-128k was numerically the highest among all curves (AUC = 0.783, *95%CI*: 0.635, 0.932); manual expert assessment (AUC = 0.746, *95%CI*: 0.593, 0.899) and standalone DeepSeek-V3 (AUC = 0.694, *95%CI*: 0.472, 0.916) ranked second and third, respectively. Only knowledge-enhanced Doubao-pro-128k and manual assessment produced AUC values significantly above the reference diagonal (*P* < 0.001 and *P* = 0.002, respectively); the standalone DeepSeek-V3 result was not statistically significant (*P* = 0.087; **Table 10**).

**Table 10:** Area under the receiver operating characteristic curves of the selected grading approaches.

Group	Area	Std. Errora	*P value*	*95% CI*
Manual expert assessment	0.746	0.078	0.002	(0.593,0.899)
DeepSeek-V3 + KB	0.598	0.105	0.354	(0.391,0.804)
DeepSeek-V3	0.694	0.113	0.087	(0.472,0.916)
Doubao-pro-128k + KB	0.783	0.076	<0.001	(0.635,0.932)
Doubao-pro-128k	0.483	0.063	0.79	(0.360,0.606)
ChatGPT-4o-Latest + KB	0.569	0.106	0.515	(0.361,0.777)
GLM-4-plus+ KB	0.635	0.097	0.164	(0.445,0.825)

AUC, area under the receiver operating characteristic curve; SE, standard error; CI, confidence interval; KB, knowledge base. AUCs, standard errors, and asymptotic 95% confidence intervals were estimated using a nonparametric empirical ROC approach. The reported P values correspond to asymptotic tests of the null hypothesis H_0_: AUC = 0.5 and do not represent pairwise comparisons between grading approaches. Pairwise comparisons of correlated ROC AUCs were performed separately using DeLong’s test, with Bonferroni correction for multiple comparisons, as reported in **Table 5**. The AUC of the standalone Doubao-pro-128k model was numerically below 0.5 but was not significantly different from the reference diagonal.

**Figure 4:**
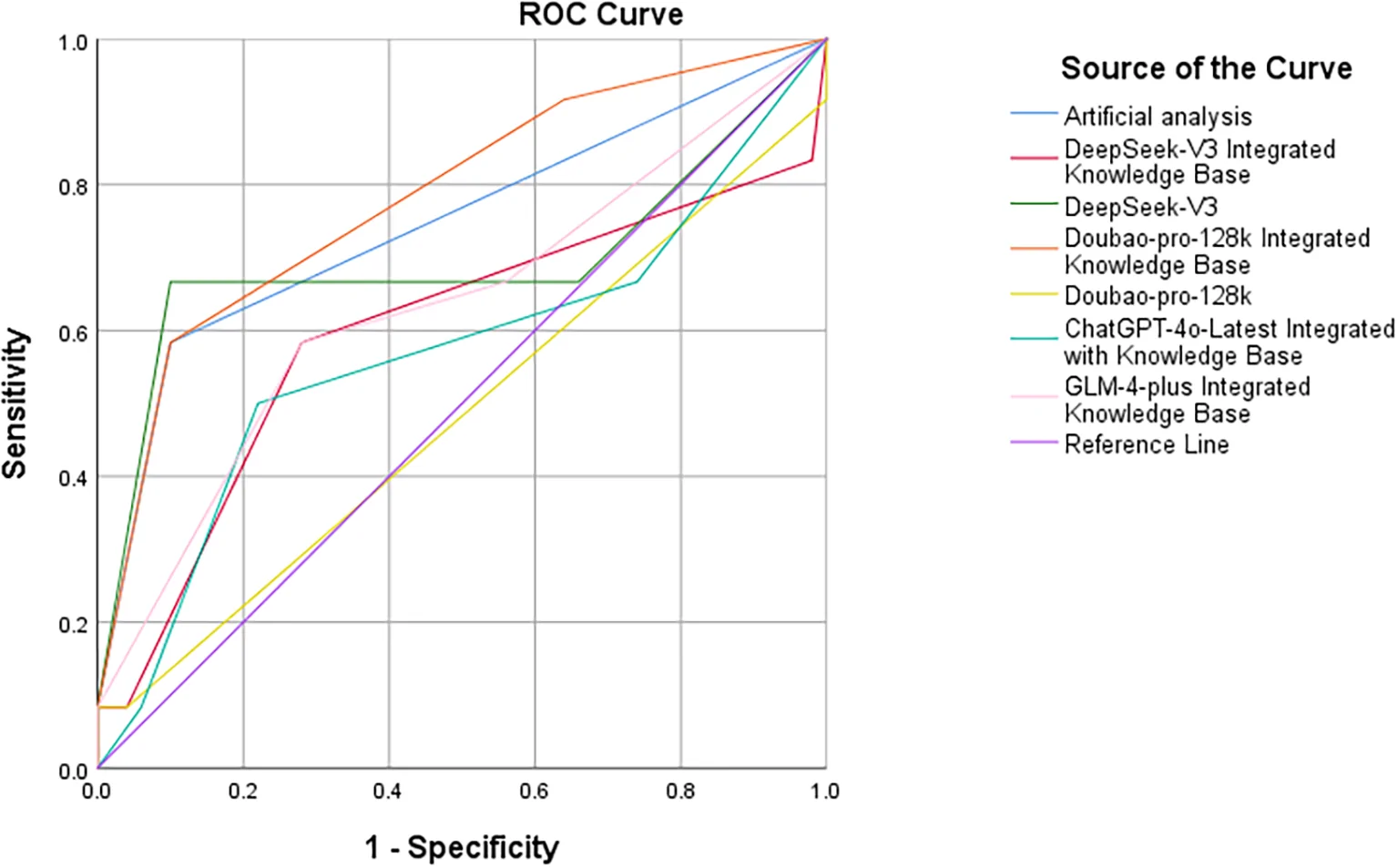
ROC analysis and performance comparison of C-TIRADS grading schemes.

**Table 11** summarizes the alignment between model-generated risk tiers and pathological outcomes for manual assessment and six LLM grading pipelines under the decision threshold C-TIRADS ≥ 4A. All alignment indicators between model risk tiers and pathological findings were calculated on the 62-case pathologically confirmed subgroup and only reflect risk stratification from text-based C-TIRADS grades. Descriptive comparison of the data in **Table 11** showed that manual expert assessment achieved perfect sensitivity (1.000, *95%CI*: 0.735, 1.000) but zero specificity, yielding a Youden index of 0.000 (*95%CI: 0.000, 0.000*), with NPV not estimable. In contrast, the knowledge-base-augmented DeepSeek-V3 demonstrated moderate sensitivity but very low specificity, resulting in a negative Youden index (−0.147, *95%CI*: −0.361, 0.068). Among all RAG-enhanced LLMs, the knowledge-enhanced Doubao-pro-128k exhibited the highest numerical sensitivity (0.917, *95%CI*: 0.615, 0.998) and Youden index (0.277, *95%CI*: 0.071, 0.482).

**Table 11:** Diagnostic performance of manual assessment and selected LLM grading pipelines at the C-TIRADS ≥ 4A threshold.

Group	The primary screening cut-off	Sensitivity(95% CI)	Specificity(95% CI)	PPV(95% CI)	NPV(95% CI)	Youden Index(95% CI)
Manual expert assessment	≥4A (cut=1.5)	1.000 (0.735, 1.000)	0.000 (0.000, 0.071)	0.194 (0.104, 0.314)	—	0.000 (0.000, 0.000)
DeepSeek-V3+ KB	≥4A (cut=1.5)	0.833 (0.516, 0.979)	0.020(0.001, 0.106)	0.169 (0.084, 0.290)	0.333(0.008, 0.906)	-0.147 (−0.361, 0.068)
DeepSeek-V3	≥4A (cut=1.5)	0.667 (0.349, 0.901)	0.340 (0.212, 0.488)	0.195 (0.088, 0.349)	0.810 (0.581, 0.946)	0.007(−0.291, 0.304)
Doubao-pro-128k+ KB	≥4A (cut=1.5)	0.917(0.615, 0.998)	0.360 (0.229, 0.508)	0.256 (0.135, 0.412)	0.947(0.740, 0.999)	0.277(0.071, 0.482)
Doubao-pro-128k	≥4A (cut=1.5)	0.917 (0.615, 0.998)	0.000 (0.000, 0.071)	0.180(0.094, 0.300)	0(0.000, 0.975)	-0.083 (−0.240, 0.073)
ChatGPT-4o-Latest + KB	≥4A (cut=1.5)	0.667 (0.349, 0.901)	0.260(0.146, 0.403)	0.178 (0.080, 0.321)	0.765 (0.501, 0.932)	-0.073(−0.366, 0.220)
GLM-4-plus + KB	≥4A (cut=1.5)	0.667 (0.349, 0.901)	0.440 (0.300, 0.587)	0.222 (0.101, 0.392)	0.846(0.651, 0.956)	0.107(−0.193, 0.407)

Sensitivity, specificity, positive predictive value (PPV), and negative predictive value (NPV) are presented with two-sided 95% Clopper–Pearson exact confidence intervals. The 95% confidence intervals for the Youden index were calculated using the delta method. The symbol “—” indicates that NPV was mathematically undefined because no negative predictions were generated (TN + FN = 0).

The *P* values reported for each AUC correspond to tests of the null hypothesis H_0_: AUC = 0.5 (reference diagonal); these *P* values do not represent pairwise comparisons between models. Doubao-pro-128k + KB (*P* < 0.001) and manual expert assessment (*P* = 0.002) achieved AUC values significantly above 0.5, whereas standalone DeepSeek-V3 showed a marginally non-significant trend (*P=0.087;* **Table 10** lists detailed AUC and 95% CI data for each pipeline). To formally assess between-model differences, pairwise DeLong tests for correlated ROC curves were performed with Doubao-pro-128k + KB as the reference (**Table 5**). After Bonferroni correction for six comparisons (corrected α=0.0083), Doubao-pro-128k+KB achieved significantly higher AUC than DeepSeek-V3+KB (*P* = 0.002), standalone Doubao-pro-128k (*P* < 0.001), and ChatGPT-4o-Latest+KB (*P* = 0.006), but did not differ significantly from manual expert consensus (*P* = 1.000), standalone DeepSeek-V3 (*P* = 1.000), or GLM-4-plus+KB (*P* = 0.066). The standalone Doubao-pro-128k model had an AUC numerically below 0.5 (AUC = 0.483), but it was not significantly different from the reference diagonal (*P* = 0.790). These exploratory findings from a small pathological subgroup require validation in larger multi-center cohorts.

## 4 Discussion

Notably, this study distinguishes grading consistency (primary endpoint) from pathological diagnostic accuracy (secondary exploratory outcome). The text-based sonographer consensus benchmark only reflects the subjective interpretations of senior sonographers reviewing identical ultrasound reports and should not be considered a diagnostic gold standard, whereas FNAC or surgical pathology provides objective ground-truth assessment of nodule benignity or malignancy. Therefore, high grading concordance between LLMs and expert text consensus only indicates alignment with human textual reasoning rather than genuine ability to differentiate nodule pathology. This fundamental distinction accounts for the divergent trends observed between text-grading agreement and pathological performance metrics. Furthermore, the pathological subgroup comprised only 62 cases, including just 12 PTC samples, resulting in wide 95% confidence intervals across all diagnostic parameters. As such, all pathological stratification findings remain exploratory and cannot define definitive clinical thresholds until validated in larger cohorts.

In this study, a FastGPT RAG knowledge base was constructed from 11,489 thyroid ultrasound records to support standardized C-TIRADS text grading. Six mainstream LLMs were evaluated under standalone and retrieval-augmented configurations, with expert text consensus as the unified reference. A head-to-head comparison of domestic and international LLMs clarifies the auxiliary value of RAG in normalizing unstructured ultrasound reports and demonstrates that this LLM-RAG pipeline serves as a text classification aid rather than an independent system for diagnosing malignancy.

Although compliance with ultrasound report documentation in tertiary hospitals nationwide reached 98.13% in 2023, inter-rater grading remains widely inconsistent across hospitals and clinicians of differing seniority (26). Most clinical ultrasound records consist of unstructured free text, which complicates processing by LLMs. Prior LLM-based studies on thyroid ultrasound reports have similarly identified this bottleneck, confirming that transforming raw text into structured feature sets markedly enhances model accuracy in nodule risk stratification (27). The 2020 C-TIRADS guideline standardizes malignancy risk stratification, yet borderline C-TIRADS 4A lesions present overlapping benign and suspicious sonographic features, making their classification heavily dependent on radiologists’ subjective judgment (28, 29). This well-known clinical ambiguity also creates unique challenges for text-based LLM grading tasks, which have not been thoroughly assessed in most existing LLM-thyroid studies (30).

Our findings indicate significant improvements in grading concordance for C-TIRADS Categories 1 and 3, while the numerical increase for Category 2 was not statistically significant; these low- and intermediate-risk nodules represent a primary source of daily inter-observer variability, particularly among less experienced sonographers. By comparison, high-risk lesions (C-TIRADS 4B-5) exhibit clear malignant features that lend themselves to consistent manual grading, leaving little room for further improvement. Nevertheless, the marked class imbalance among high-risk cases in this single-center cohort limits the generalizability of the observed benefits of RAG to C-TIRADS 4B-5 nodules.

Several plausible contributing hypotheses may partly explain the observed numerical decline in grading concordance for C-TIRADS 4A nodules following the integration of the knowledge base. First, C-TIRADS 4A lesions rely on a small set of non-distinct suspicious sonographic markers, which are vulnerable to ambiguous interpretation by models (31). Second, the knowledge base includes numerous actual clinical cases with overlapping features between benign and low-risk malignant 4A nodules, which could potentially increase the difficulty of definitive classification (32). Third, limited explicit scoring guidance for C-TIRADS 4A may trigger divergent reasoning pathways when models process similar but non-identical feature sets. These issues are also commonly encountered in manual grading, indicating that the classification of C-TIRADS 4A nodules remains challenging for both AI and sonographers (33). It should be emphasized that these mechanistic explanations remain speculative and cannot be directly validated with the current study dataset.

Although the numerically reduced concordance with manual grading for C-TIRADS 4A lesions after RAG augmentation might at first seem inconsistent with the tentatively observed pathological stratification signals from knowledge-base-augmented Doubao-pro-128k, the two outcomes are logically compatible. Text-grading concordance measures alignment against local sonographer subjective consensus biased by ambiguous borderline signs, whereas pathological-oriented metrics quantify model fit against objective histopathological gold standards. The RAG knowledge base contains large volumes of real-world borderline 4A clinical cases. This approach may mitigate the impact of single-center sonographer subjective risk-tier bias on model outputs. It may also yield grading outputs that better align with real-world malignant risk distribution among thyroid lesions. This discrepancy between human-text-matching consistency and pathological risk-stratification performance does not indicate model underperformance. Nevertheless, these preliminary observations cannot be interpreted as proven clinical advantages of domain-specific RAG retrieval for clinical auxiliary grading systems. Definitive validation requires larger multi-center pathological cohorts (34).

The severe scarcity of C-TIRADS 4B/4C/5 samples limits robust pathological verification of high-risk nodules. Although all pathologically confirmed lesions categorized as ≥4A were retained for exploratory ROC and trend analysis, the limited number of high-risk samples does not support stable conclusions for advanced risk tiers. In this context, the significant trend observed for Doubao-pro-128k integrated with the knowledge base suggested an ascending gradient of malignant risk; this exploratory finding may indicate that retrieval of clinical cases helps calibrate model grading logic to pathological distribution patterns (35).

General-domain transformer-based models show strong feature extraction capabilities across diverse biomedical tasks (36, 37). Compared with prior literature focusing on LLM-assisted thyroid nodule report interpretation, the present study bears several distinct methodological and observational differences. Most published LLM-thyroid studies either adopted multimodal image-input workflows or evaluated international TIRADS systems (ACR-TIRADS, K-TIRADS) rather than the domestic C-TIRADS guideline on raw Chinese free-text ultrasound reports (15, 38). In our thyroid-specific scenario, six LLMs accurately extracted ultrasonic features from textual reports, providing support for report structuring and standardized C-TIRADS grading, consistent with recent thyroid-oriented LLM investigations (30, 39, 40). One plausible hypothesis for this discrepancy is that DeepSeek-V3 may have limited exposure to relevant clinical data regarding the association between C-TIRADS 4A nodules and malignant risk. Given the wide malignant risk range for C-TIRADS 4A (2%-10%), the high population base of 4A nodules, non-standard noisy clinical samples could conceivably be present within training corpora. When such search noise is extensively incorporated into training sets, which might impair grading stability (41).This proposed mechanism cannot be directly validated in our study, as we lack access to the internal training datasets of commercial LLMs.

Papageorgiou PS et al. (42) proved that RAG supplements LLMs with external specialized corpora to boost output consistency and reliability, few studies have validated such improvements on Chinese thyroid ultrasound C-TIRADS text-grading tasks, particularly for real-world borderline nodule cases (39). This study employed the FastGPT integrated with knowledge base Q&A system to develop an knowledge-base-augmented LLM for thyroid nodule report classification and C-TIRADS risk stratification. By configuring RAG-related technical parameters—including a hybrid retrieval strategy combining vector-based and full-text search, a relevance coefficient decision threshold set above 0.9, the RRF algorithm for ranking integrated retrieval results, and a maximum citation limit of 1,500 entries—we optimized retrieval outputs to ensure high reliability of the retrieved clinical knowledge fragments for C-TIRADS risk tiering. After knowledge-base integration, classification concordance improved significantly for C-TIRADS Categories 1 and 3, increased numerically for Category 2, and decreased numerically without statistical significance for Category 4A. K Singhal et al. (43) showed that embedding extensive clinical knowledge into an LLM improved its consistency on medical exam questions by more than 17%. Our multi-system thyroid ultrasound case library supplies abundant standardized feature references for model reasoning.

From the perspective of domestic and international existing research, most prior LLM thyroid imaging grading studies focus on image visual recognition or single foreign LLMs such as GPT-4 for ACR-TIRADS assessment, lacking head-to-head comparison of mainstream Chinese-adapted LLMs under the unified domestic C-TIRADS guideline framework (39, 44). Consistent with the technical conclusion of Papageorgiou DeBellis M et al., the present work further validates that RAG domain knowledge embedding can stably lift the grading consistency of medical LLMs (45). Different from pixel-level image recognition AI, our text-oriented pipeline meets real clinical demands for report quality control and cross-hospital result interoperability. The superior performance of Doubao-pro-128k-RAG further highlights that native Chinese large language models, when integrated with local clinical databases, can outperform English-origin models on domestic ultrasound text processing tasks.

All six standalone LLMs had raw P values below 0.05 for agreement with manual grading, although five of six remained significant after table-specific Bonferroni correction; RAG integration raised four of the six Kappa values above 0.8. When grading concordance was assessed via linear weighted Kappa with stratified Bonferroni correction separated for standalone LLMs (**Table 1**) and knowledge-base-augmented LLM pipelines (**Table 2**), most standalone LLMs and all knowledge base integrated with models exhibited statistically significant consistency with expert sonographer grading standards. Stratified correction reduced statistical bias associated with multiple comparisons and confirmed the stability of the optimization improvements obtained using the RAG framework. This finding matches a previous meta-analysis (46), which confirmed AI tools unify risk assessment and reduce inter-operator discrepancy for junior radiologists.

LLM pipelines significantly reduce the manual effort involved in grading, while RAG enhances consistency without compromising efficiency—findings that align with those of Tian M (47). DeepSeek-V3 and Doubao-pro-128k achieved high risk tier matching consistency with expert text grading in this study. A comparison of processing times between these models and their knowledge base integrated with versions revealed that Doubao-pro-128k required less time. This difference may be attributed to variations in model architecture, task processing strategies, and server concordance.

Knowledge-base augmentation significantly improved overall grading concordance, with category-specific significance for C-TIRADS Categories 1 and 3; the increase for Category 2 was numerical but not statistically significant. The optimized framework mitigates grading discrepancies arising from unequal experience between senior and junior sonographers, thus standardizing the subjective interpretation of ambiguous low- and intermediate-risk nodule features. The ultra-fast automated grading also enables continuous batch quality control of daily ultrasound reports without adding human labor burden. Moreover, unified standardized text outputs create consistent datasets suited for multi-center thyroid epidemiological research and cross-hospital patient follow-up communication. Nevertheless, all performance advantages observed in this work are restricted to report-level standardization and improvement in inter-rater grading consistency within our single-center dataset. This single-center retrospective text study cannot verify downstream clinical endpoints such as reduced misdiagnosis rates, modified biopsy rates or improved long-term patient prognosis, even if these endpoints represent desirable future goals. Prospective multi-institutional pathological cohorts are required for further validation of such clinical benefits.

This LLM-RAG grading framework shows measurable performance benefits only within the present single-center retrospective cohort; its clinical translation value for tiered medical institutions across the country remains unproven and awaits multi-center external validation. Within our local dataset, this pipeline increased concordance for C-TIRADS 1-3 nodules, with statistically significant category-specific improvements for Categories 1 and 3, and supported structured processing of unstructured ultrasound texts. These single-center findings suggest potential future benefits restricted to text-report standardization: following successful multi-center external validation, this tool may help standardize disorganized free-text reports in grassroots-level hospitals and mitigate grading discrepancies originating from uneven sonographer clinical experience (39). High-throughput automatic grading observed in this study holds potential for real-time daily report quality-control without substantial additional human workload. However, any meaningful clinical value beyond standardizing text-level grading has yet to be demonstrated in real-world, multi-hospital settings. Strict clinical safety protocols must be upheld: all AI-generated C-TIRADS 4A findings are subject to mandatory review by attending sonographers. Model outputs should serve only as supplementary references for text-based grading and must not be used independently to inform biopsy or surgical decisions—particularly given the limited pathological validation in this study (48). Additionally, the hospital-intranet-based deployment strategy—with locally hosted open-source models operating fully offline and closed-source models accessed only via de-identified data through a controlled outbound gateway— complies with Chinese regulatory requirements for personal health data, and provides a feasible privacy-preserving technical option for any future real-world deployments.

Future research should address the limitations of this single-center work along two dimensions. First, multi-center external validation covering multi-equipment and multi-operator datasets from hospitals of different tiers is an essential prerequisite before any nationwide clinical implementation or standardization. Second, further optimization of RAG retrieval algorithms and model-fusion strategies should refine feature-assessment criteria for borderline C-TIRADS 4A nodules to lower inter-observer grading variability.

## 5 Limitations

Limitations of this study should be acknowledged. We acknowledge several limitations of this study. First, the full test set exhibited severe imbalance across risk subgroups: merely 11 C-TIRADS 4B cases, zero C-TIRADS 4C cases, and only one C-TIRADS 5 case. This uneven distribution aligns with standardized clinical management norms in Chinese tertiary hospitals, where all nodules graded ≥C-TIRADS 4A are routinely recommended for pathological biopsy, leading to very few untreated ultra-high-risk nodules in retrospective ultrasound datasets. Two independent analytical strategies were adopted for different research endpoints: for stratified categorical consistency comparison between standalone LLMs and LLMs integrated with knowledge base, all high-risk tiers (C-TIRADS 4B-5) were excluded to avoid statistical instability triggered by sparse high-risk samples; for exploratory pathological subgroup analysis targeting lesions manually graded ≥4A with pathological gold-standard results, all available C-TIRADS 4B and 5 cases were reserved without exclusion. Although overall grading consistency improved, category-specific changes were mixed and the numerical decline for C-TIRADS 4A was not statistically significant; this study was not designed to evaluate downstream clinical impacts, including changes in biopsy rates, surgical decision-making, or patient outcomes. Second, the test set included 228 thyroid nodules, a relatively small sample for external validation, which may limit the statistical reliability of concordance estimates for rare high-risk C-TIRADS 4B, 4C, and 5 nodules associated with elevated malignant potential. Third, the dataset lacked multi-device, multi-operator, and multi-center validation, so potential equipment-related bias and inter-institutional variability could not be fully assessed. Fourth, despite rigorous data preprocessing, residual noise from unstandardized free-text descriptions in historical reports may still affect knowledge base retrieval and LLM reasoning, especially for ambiguous C-TIRADS 4A nodules (27). Fifth, the pathological exploratory subgroup had a small sample size and severe class imbalance, with extremely limited high-risk malignant nodules. All AUC and trend chi-square alignment indicators are exploratory only with insufficient statistical power and cannot verify the generalizability of model-pathology matching for clinical reference. Sixth, although pairwise DeLong tests were performed to compare ROC AUCs between grading pipelines, the small pathological subgroup (n = 62, 12 PTC) provides limited statistical power, particularly for detecting moderate effect sizes. Non-significant pairwise comparisons should not be interpreted as evidence of equivalence; larger cohorts are required to definitively establish between-model differences or equivalence.

Future studies should incorporate larger, more diverse multi-center datasets from hospitals at different levels, use various ultrasound systems, and include more cases of rare malignant PTC subtypes to reduce bias and improve external validity. Further optimization of algorithms, ensemble learning, and multi-model fusion are also needed to improve the grading stability and generalizability of LLMs in thyroid nodule report classification (49–52).Meanwhile, updates to thyroid classification guidelines could refine the scoring criteria for C-TIRADS 4A, offering more accurate and robust standards for C-TIRADS grading of borderline nodule.

Clinical implementation challenges include model explainability, compliance with data privacy regulations such as GDPR and HIPAA, and the ethical implications of AI-assisted grading, all of which require further validation before routine adoption (53).

## 6 Conclusion

In the present cohort, RAG-augmented Doubao-pro-128k accelerated automated text grading, while overall grading concordance improved after knowledge-base integration; category-specific concordance increased significantly for C-TIRADS Categories 1 and 3, increased numerically for Category 2, and declined numerically without statistical significance for ambiguous borderline Category 4A, highlighting the need for targeted algorithm optimization for this category. Improved alignment with expert textual consensus does not equate to enhanced pathological malignancy discrimination, and severe under-sampling of C-TIRADS 4B-5 high-risk nodules limits generalizability of grading-concordance results to high-risk subgroups.

All pathological-risk-stratification findings derive from a small, imbalanced exploratory subgroup of only 62 pathologically confirmed nodules with low statistical power. At the C-TIRADS≥4A cutoff, the knowledge-enhanced Doubao-pro-128k achieved numerically the highest Youden index (0.277, 95%CI: 0.071, 0.482). Collectively, the LLM-RAG framework showed cohort-specific benefits for text-level grading standardization, with significant category-specific concordance gains for C-TIRADS Categories 1 and 3, yet no robust generalizable evidence supports its performance for pathological risk discrimination. Broad clinical deployment is not justified at this stage. Large multi-center cohorts enriched with pathologically confirmed high-risk thyroid nodules are required to externally validate both grading consistency and malignancy-stratification performance prior to real-world clinical adoption.

## Data Availability

The original contributions presented in the study are included in the article/**Supplementary Material**. Further inquiries can be directed to the corresponding authors.
