# Supplementary material for: Application of LLM combined with knowledge base in improving C-TIRADS grading of thyroid nodules
Source: Front Endocrinol (Lausanne). 2026 Oct 15;17:1888772. doi: 10.3389/fendo.2026.1888772 (PMC13716552; doi:10.3389/fendo.2026.1888772)
Supplement: Supplementary file 2 [file SupplementaryFile2.pdf]

Table S2 Confusion Matrix (Total 228 thyroid nodules, ground truth distribution from manual assessment)

Ground Truth Class Distribution

C-TIRADS 1:46, C-TIRADS 2:21, C-TIRADS 3:99, C-TIRADS 4A:50, C-TIRADS 4B:11, C-TIRADS 5:1

Total nodules = 46+21+99+50+11+1 = 228

**Ground Truth \ Prediction**	**C-TIRADS 1**	**C-TIRADS 2**	**C-TIRADS 3**	**C-TIRADS 4A**	**C-TIRADS 4B**	**C-TIRADS 5**	**Row Total**
C-TIRADS 1	46	0	0	0	0	0	46
C-TIRADS 2	0	20	1	0	0	0	21
C-TIRADS 3	0	2	95	2	0	0	99
C-TIRADS 4A	0	0	1	48	1	0	50
C-TIRADS 4B	0	0	0	1	10	0	11
C-TIRADS 5	0	0	0	0	0	1	1
Column Total	46	22	97	51	11	1	228

Final Accuracy Check

Correct = 46 + 20 + 95 + 48 + 10 + 1 = 220

Overall concordance = 220 / 228 = 0.9649 = 96.49%
