# Supplementary material for: Application of LLM combined with knowledge base in improving C-TIRADS grading of thyroid nodules
Source: Front Endocrinol (Lausanne). 2026 Oct 15;17:1888772. doi: 10.3389/fendo.2026.1888772 (PMC13716552; doi:10.3389/fendo.2026.1888772)
Supplement: Supplementary file 3 [file SupplementaryFile3.pdf]

Ethics approval document issued by the Ethics Committee of Yantai Yuhuangding Hospital (approval number: 2024-608; approval date: June 4, 2024).

烟台毓璜顶医院伦理委员会审批件

批件号：烟毓医伦理审【 2024-608 】号

项目名称	大语言模型结合知识库在优化甲状腺结节 C-TIRADS 分级中的应用				
研究涉及科室	超声医学科中区		项目负责人	刘真	
采集样本或信息类型	甲状腺超声自由文本报告		采集样本或信息数量	建模库 11489 份，测试集 228 份	
是否涉及样本、信息与国内外合作使用	无		合作单位或公司	无	
表决情况	表决人数	同意	做必要修改后同意	不同意	中止或暂停已批准的试验
	11	11	0	0	0

项目简介

甲状腺癌全球发病率逐年升高，超声是甲状腺结节首选影像学筛查手段，但临床 C-TIRADS 分级存在显著医师间、院间主观判读差异，标准化报告体系建设存在难点。本研究基于检索增强生成（RAG）技术搭建 FastGPT 甲状腺专科知识库，系统对比 DeepSeek-V3、ChatGPT-4o-Latest、GPT-3.5-Turbo、Doubao-pro-128k、GLM-4-plus、Qwen-plus 六种主流大语言模型，仅依托清洗脱敏后的甲状腺超声纯文本特征描述，完成标准化 C-TIRADS 分级，定量评估各类模型与高年资超声专家分级的一致性、分级推理耗时。研究全程不开展任何临床干预、不接触患者本人，无需采集活体组织、血液等人体生物样本，不涉及人类遗传资源使用，无国内、国际合作单位。

本课题郑重承诺：本回顾性文本数据研究方案符合国内医疗数据保护法规、临床研究伦理标准与国际通用医学研究惯例，数据存储、调用、分析全过程严格遵循《个人信息保护法》，严格保护患者隐私，全程规避数据泄露风险。

项目实施日期：2024 年 06 月 01 日 —— 2025 年 06 月 30 日

是否签署知情同意书：☐是 ☑否

涉及人类遗传资源使用情况：无

国内外审批备案情况：无

国内及国际合作情况：无

科研伦理委员会意见

同意

2024-06-04 15：38

审批意见：

伦理委员会于 2024 年 06 月 04 日对上述实施方案进行认真讨论并表决，认为主要研究者具备超声临床科研资质，研究方案设计科学、合理、可行；本研究仅使用完全脱敏后的历史超声文本报告，无患者直接接触、无侵入性临床操作，患者隐私保护措施完善，研究潜在风险远低于研究预期临床价值；全部 AI 模型院内本地离线部署，符合医疗数据安全管理规范，同意按该方案开展回顾性研究。

本批文有效期自签字之日起一年。

主任委员签名：

会议地点：烟台毓璜顶医院行政楼 518 楼会议室

2024 年 06 月 04 日
