# Supplementary material for: Application of LLM combined with knowledge base in improving C-TIRADS grading of thyroid nodules
Source: Front Endocrinol (Lausanne). 2026 Oct 15;17:1888772. doi: 10.3389/fendo.2026.1888772 (PMC13716552; doi:10.3389/fendo.2026.1888772)
Supplement: Supplementary file 4 [file SupplementaryFile1.pdf]

Supplementary Material S1. Technical Details for Reproducibility

补充材料 S1：可复现性相关技术细节

1. Standardization of Terminology:

1.术语标准化：

"低回声" (Hypoechoic) and "极低回声" (Very hypoechoic) were retained as distinct entities.

“Hypoechoic”（低回声）与 “Very hypoechoic”（极低回声）被划分为不同的影像特征类别予以区分保留。

"强回声灶" (Hyperechoic foci) was standardized to "calcification".

"强回声灶"（高回声病灶）统一规范表述为 "钙化灶"

"纵横比>1" (Taller-than-wide) was standardized to "aspect ratio > 1".

“纵横比> 1”（竖长型）统一规范为 “长宽比 > 1”。

Noise Reduction Processing: Remove special characters (e.g., *, #, &), redundant spaces, line breaks, and irrelevant administrative texts (such as "Please correlate with clinical findings...", "Patient self-reported...")

降噪处理：移除特殊字符（如 *、#、&）、多余空格，回车以及无关管理类文本（如 “请结合临床……”“患者自述……”）

2. Structured Report Template (Output of GLM-4 and Input for Grading LLMs)

2.结构化报告模板（GLM-4 的输出内容、大模型评分工具的输入内容）

The GLM-4 model was instructed to convert cleaned text into the following JSON-like structure. This structured text was used as the final input for the six LLMs.

GLM-4 模型被指示将清洗后的文本转换为如下类 JSON 结构。该结构化文本作为六大大型语言模型的最终输入。

```
json
{
report_1 = [
    "左叶结节特点：",
    [
        ["结节回声特点：", ["无回声"]],
        ["结节形态特点：", ["规则形态"]],
        ["结节大小：", ["约 0.3cm*0.2cm"]],
        ["结节纵横比：", ["纵横比<1"]],
        ["结节边界特点：", ["清晰边界"]],
        ["内部回声是否均匀：", ["无明显特点"]],
        ["有无微钙化：", ["无数据"]],
        ["有无点状强回声：", ["无数据"]],
        ["后方回声特点：", ["无明显变化"]]
    ],
    "右叶结节特点：",
    [
        ["结节回声特点：", ["无回声"]],
        ["结节形态特点：", ["规则形态"]],
        ["结节大小：", ["约 0.9cm*0.5cm"]],
        ["结节纵横比：", ["纵横比<1"]],
        ["结节边界特点：", ["清晰边界"]],
```

```
            ["内部回声是否均匀：", ["无明显特点"]],
            ["有无微钙化：", ["无数据"]],
            ["有无点状强回声：", ["无数据"]],
            ["后方回声特点：", ["无明显变化"]]
      ]
]
report_1 = [
"Characteristics of Left Lobe Nodules:",
[
["Nodule Echogenicity:", ["Anechoic"]],
["Nodule Morphology:", ["Regular shape"]],
["Nodule Size:", ["Approx. 0.3cm0.2cm"]],
["Nodule Aspect Ratio:", ["Aspect ratio < 1"]],
["Nodule Margin:", ["Clear margin"]],
["Homogeneity of Internal Echoes:", ["No remarkable features"]],
["Presence of Microcalcifications:", ["No data available"]],
["Presence of Punctate Hyperechoic Foci:", ["No data available"]],
["Posterior Echo Features:", ["No significant changes"]]
],
"Characteristics of Right Lobe Nodules:",
[
["Nodule Echogenicity:", ["Anechoic"]],
["Nodule Morphology:", ["Regular shape"]],
["Nodule Size:", ["Approx. 0.9cm0.5cm"]],
["Nodule Aspect Ratio:", ["Aspect ratio < 1"]],
["Nodule Margin:", ["Clear margin"]],
["Homogeneity of Internal Echoes:", ["No remarkable features"]],
["Presence of Microcalcifications:", ["No data available"]],
["Presence of Punctate Hyperechoic Foci:", ["No data available"]],
["Posterior Echo Features:", ["No significant changes"]]
]
]
report_2 = [
      "甲状腺双侧叶及峡部特点：",
      [
            ["大小：", ["尚可"]],
            ["形态：", ["尚可"]],
            ["包膜：", ["尚清晰"]],
            ["实质回声：", ["尚均匀"]],
            ["结节特点：", ["未探及明显结节及液性暗区"]],
            ["CDFI：", ["未见异常血流信号"]],
            ["周围淋巴结：", ["未探及明显肿大淋巴结"]]
      ]
]
```

```
report_2 = [
"Features of bilateral thyroid lobes and isthmus:",
[
["Size:", ["Fairly normal"]],
["Shape:", ["Fairly normal"]],
["Capsule:", ["Fairly clear"]],
["Parenchymal echo:", ["Fairly homogeneous"]],
["Nodule features:", ["No obvious nodules or fluid dark areas detected"]],
["CDFI:", ["No abnormal blood flow signals seen"]],
["Peripheral lymph nodes:", ["No obviously enlarged lymph nodes detected"]]
]
]
report_3 = [
    "左叶结节特点：",
    [
        ["结节回声特点：", ["低回声"]],
        ["结节形态特点：", ["欠规则形态"]],
        ["结节大小：", ["约 0.4cm*0.4cm"]],
        ["结节纵横比：", ["纵横比=1"]],
        ["结节边界特点：", ["欠清晰边界"]],
        ["内部回声是否均匀：", ["欠均匀"]],
        ["有无微钙化：", ["无数据"]],
        ["有无点状强回声：", ["无数据"]],
        ["后方回声特点：", ["无明显变化"]]
    ],
    "右叶结节特点：",
    [
        ["结节回声特点：", ["无回声"]],
        ["结节形态特点：", ["规则形态"]],
        ["结节大小：", ["约 0.9cm*0.5cm"]],
        ["结节纵横比：", ["纵横比<3"]],
        ["结节边界特点：", ["清晰边界"]],
        ["内部回声是否均匀：", ["无明显特点"]],
        ["有无微钙化：", ["无数据"]],
        ["有无点状强回声：", ["无数据"]],
        ["后方回声特点：", ["无明显变化"]]
    ]
]
report_3 = [
"Characteristics of Left Lobe Nodules:",
[
["Nodule Echogenicity Feature:", ["Hypoechoic"]],
["Nodule Morphology Feature:", ["Irregular shape"]],
["Nodule Size:", ["Approx. 0.4cm0.4cm"]],
```

```
["Nodule Aspect Ratio:", ["Aspect ratio = 1"]],
["Nodule Border Feature:", ["Indistinct border"]],
["Homogeneity of Internal Echo:", ["Slightly heterogeneous"]],
["Presence of Microcalcifications:", ["No data"]],
["Presence of Punctate Hyperechoic Foci:", ["No data"]],
["Posterior Echo Feature:", ["No significant change"]]
],
"Characteristics of Right Lobe Nodules:",
[
["Nodule Echogenicity Feature:", ["Anechoic"]],
["Nodule Morphology Feature:", ["Regular shape"]],
["Nodule Size:", ["Approx. 0.9cm0.5cm"]],
["Nodule Aspect Ratio:", ["Aspect ratio < 3"]],
["Nodule Border Feature:", ["Distinct border"]],
["Homogeneity of Internal Echo:", ["No remarkable feature"]],
["Presence of Microcalcifications:", ["No data"]],
["Presence of Punctate Hyperechoic Foci:", ["No data"]],
["Posterior Echo Feature:", ["No significant change"]]
]
]
report_4 = [
    "左叶结节特点：",
    [
        ["结节回声特点：", ["无回声"]],
        ["结节形态特点：", ["规则形态"]],
        ["结节大小：", ["约 0.3cm*0.2cm"]],
        ["结节纵横比：", ["纵横比<1"]],
        ["结节边界特点：", ["清晰边界"]],
        ["内部回声是否均匀：", ["无明显特点"]],
        ["有无微钙化：", ["无数据"]],
        ["有无点状强回声：", ["无数据"]],
        ["后方回声特点：", ["无明显变化"]]
    ],
    "右叶结节特点：",
    [
        ["结节回声特点：", ["无回声"]],
        ["结节形态特点：", ["规则形态"]],
        ["结节大小：", ["约 0.9cm*0.5cm"]],
        ["结节纵横比：", ["纵横比<4"]],
        ["结节边界特点：", ["清晰边界"]],
        ["内部回声是否均匀：", ["无明显特点"]],
        ["有无微钙化：", ["无数据"]],
        ["有无点状强回声：", ["无数据"]],
        ["后方回声特点：", ["无明显变化"]]
```

```
        ]
]
report_4 = [
"Characteristics of left lobe nodule:",
[
["Nodule echogenicity:", ["Anechoic"]],
["Nodule morphology:", ["Regular shape"]],
["Nodule size:", ["Approx. 0.3cm0.2cm"]],
["Nodule anteroposterior-transverse ratio:", ["Ratio < 1"]],
["Nodule margin:", ["Well-defined margin"]],
["Homogeneity of internal echo:", ["No remarkable features"]],
["Presence of microcalcifications:", ["No data"]],
["Presence of punctate hyperechoic foci:", ["No data"]],
["Posterior echo feature:", ["No significant change"]]
],
"Characteristics of right lobe nodule:",
[
["Nodule echogenicity:", ["Anechoic"]],
["Nodule morphology:", ["Regular shape"]],
["Nodule size:", ["Approx. 0.9cm0.5cm"]],
["Nodule anteroposterior-transverse ratio:", ["Ratio < 4"]],
["Nodule margin:", ["Well-defined margin"]],
["Homogeneity of internal echo:", ["No remarkable features"]],
["Presence of microcalcifications:", ["No data"]],
["Presence of punctate hyperechoic foci:", ["No data"]],
["Posterior echo feature:", ["No significant change"]]
]
]
report_5 = [
        "左叶结节特点：",
        [
                ["结节回声特点：", ["无回声"]],
                ["结节形态特点：", ["规则形态"]],
                ["结节大小：", ["约 0.3cm*0.2cm"]],
                ["结节纵横比：", ["纵横比<1"]],
                ["结节边界特点：", ["清晰边界"]],
                ["内部回声是否均匀：", ["无明显特点"]],
                ["有无微钙化：", ["无数据"]],
                ["有无点状强回声：", ["无数据"]],
                ["后方回声特点：", ["无明显变化"]]
        ],
        "右叶结节特点：",
        [
                ["结节回声特点：", ["无回声"]],
```

```
        ["结节形态特点：", ["规则形态"]],
        ["结节大小：", ["约 0.9cm*0.5cm"]],
        ["结节纵横比：", ["纵横比<5"]],
        ["结节边界特点：", ["清晰边界"]],
        ["内部回声是否均匀：", ["无明显特点"]],
        ["有无微钙化：", ["无数据"]],
        ["有无点状强回声：", ["无数据"]],
        ["后方回声特点：", ["无明显变化"]]
    ]
]
report_5 = [
"Characteristics of left lobe nodule:",
[
["Nodule echogenicity characteristics:", ["Anechoic"]],
["Nodule morphological characteristics:", ["Regular shape"]],
["Nodule size:", ["Approx. 0.3cm0.2cm"]],
["Nodule aspect ratio:", ["Aspect ratio < 1"]],
["Nodule boundary characteristics:", ["Clear boundary"]],
["Uniformity of internal echo:", ["No obvious features"]],
["Presence of microcalcifications:", ["No data"]],
["Presence of punctate hyperechoic foci:", ["No data"]],
["Posterior echo characteristics:", ["No obvious changes"]]
],
"Characteristics of right lobe nodule:",
[
["Nodule echogenicity characteristics:", ["Anechoic"]],
["Nodule morphological characteristics:", ["Regular shape"]],
["Nodule size:", ["Approx. 0.9cm0.5cm"]],
["Nodule aspect ratio:", ["Aspect ratio < 5"]],
["Nodule boundary characteristics:", ["Clear boundary"]],
["Uniformity of internal echo:", ["No obvious features"]],
["Presence of microcalcifications:", ["No data"]],
["Presence of punctate hyperechoic foci:", ["No data"]],
["Posterior echo characteristics:", ["No obvious changes"]]
]
]
report_6 = [
    "甲状腺双侧叶及峡部特点：",
    [
        ["术后特点：", ["局部未探及明显异常回声"]],
        ["左叶特点：", ["大小形态尚可", "实质回声尚均匀", "未见明确结节"]],
        ["CDFI：", ["未见异常血流信号"]],
        ["周围淋巴结：", ["未探及明显增大淋巴结"]]
    ]
```

```
]

report_7 = [
    "甲状腺双侧叶及峡部特点：",
    [
        ["大小形态：", ["体积正常", "形态尚可"]],
        ["左叶结节特点：",
            [
                ["结节回声特点：", ["低回声"]],
                ["结节形态特点：", ["欠规则形态"]],
                ["结节大小：", ["约 1.3cm*0.6cm"]],
                ["结节纵横比：", ["纵横比<1"]],
                ["结节边界特点：", ["清晰边界"]],
                ["内部回声是否均匀：", ["欠均匀"]],
                ["有无微钙化：", ["无数据"]],
                ["有无点状强回声：", ["无数据"]],
                ["后方回声特点：", ["无明显变化"]],
                ["CDFI：", ["结节内可见少量血流信号"]]
            ]
        ],
        ["右叶结节特点：",
            [
                ["结节回声特点：", ["低回声"]],
                ["结节形态特点：", ["规则形态"]],
                ["结节大小：", ["约 1.0cm*0.6cm"]],
                ["结节纵横比：", ["纵横比<1"]],
                ["结节边界特点：", ["清晰边界"]],
                ["内部回声是否均匀：", ["欠均匀"]],
                ["有无微钙化：", ["无数据"]],
                ["有无点状强回声：", ["无数据"]],
                ["后方回声特点：", ["无明显变化"]],
                ["CDFI：", ["结节内部及周边可见较丰富血流信号"]]
            ]
        ],
        ["峡部结节特点：",
            [
                ["结节回声特点：", ["低回声"]],
                ["结节大小：", ["约 1.5cm*0.5cm"]],
                ["结节纵横比：", ["纵横比<1"]],
                ["结节边界特点：", ["清晰边界"]],
                ["内部回声是否均匀：", ["欠均匀"]],
                ["后方回声特点：", ["无明显变化"]],
                ["CDFI：", ["结节内部及周边可见较丰富血流信号"]],
                ["余腺体特点：", ["血流信号较丰富"]]
```

]
],
["周围淋巴结：", ["未探及明显增大淋巴结"]]
]
]
report_6 = [
"Characteristics of bilateral thyroid lobes and isthmus:",
[
["Postoperative characteristics:", ["No obvious abnormal echoes detected locally"]],
["Characteristics of left lobe:", ["Normal size and shape", "Uniform parenchymal echoes", "No definite nodules observed"]],
["CDFI:", ["No abnormal blood flow signals detected"]],
["Peripheral lymph nodes:", ["No obviously enlarged lymph nodes detected"]]
]
]
report_7 = [
"Characteristics of bilateral thyroid lobes and isthmus:",
[
["Size and shape:", ["Normal volume", "Acceptable shape"]],
["Characteristics of nodules in left lobe:",
[
["Echo characteristics of nodule:", ["Hypoechoic"]],
["Morphological characteristics of nodule:", ["Irregular shape"]],
["Size of nodule:", ["Approx. 1.3cm0.6cm"]],
["Nodule aspect ratio:", ["Aspect ratio < 1"]],
["Boundary characteristics of nodule:", ["Clear boundary"]],
["Uniformity of internal echoes:", ["Slightly uneven"]],
["Presence of microcalcification:", ["No data"]],
["Presence of punctate strong echoes:", ["No data"]],
["Posterior echo characteristics:", ["No obvious changes"]],
["CDFI:", ["A small amount of blood flow signals visible within the nodule"]]
]
],
["Characteristics of nodules in right lobe:",
[
["Echo characteristics of nodule:", ["Hypoechoic"]],
["Morphological characteristics of nodule:", ["Regular shape"]],
["Size of nodule:", ["Approx. 1.0cm0.6cm"]],
["Nodule aspect ratio:", ["Aspect ratio < 1"]],
["Boundary characteristics of nodule:", ["Clear boundary"]],
["Uniformity of internal echoes:", ["Slightly uneven"]],
["Presence of microcalcification:", ["No data"]],
["Presence of punctate strong echoes:", ["No data"]],
["Posterior echo characteristics:", ["No obvious changes"]],

```
["CDFI:", ["Relatively abundant blood flow signals visible inside and around the nodule"]]
]
],
["Characteristics of nodules in isthmus:",
[
["Echo characteristics of nodule:", ["Hypoechoic"]],
["Size of nodule:", ["Approx. 1.5cm*0.5cm"]],
["Nodule aspect ratio:", ["Aspect ratio < 1"]],
["Boundary characteristics of nodule:", ["Clear boundary"]],
["Uniformity of internal echoes:", ["Slightly uneven"]],
["Posterior echo characteristics:", ["No obvious changes"]],
["CDFI:", ["Relatively abundant blood flow signals visible inside and around the nodule"]],
["Characteristics of remaining gland tissue:", ["Relatively abundant blood flow signals"]]
]
],
["Peripheral lymph nodes:", ["No obviously enlarged lymph nodes detected"]]
]
]
report_8 = [
    "甲状腺双侧叶及峡部特点：",
    [
        ["大小形态：", ["体积正常", "形态尚可"]],
        ["左叶结节特点：",
            [
                ["结节回声特点：", ["低回声"]],
                ["结节形态特点：", ["规则形态"]],
                ["结节大小：", ["约 2.5cm*1.5cm*1.8cm"]],
                ["结节纵横比：", ["纵横比<1"]],
                ["结节边界特点：", ["清晰边界"]],
                ["内部回声是否均匀：", ["欠均匀"]],
                ["后方回声特点：", ["无明显变化"]],
                ["CDFI：", ["结节内实性部分可见少量血流信号"]]
            ]
        ],
        ["右叶结节特点：",
            [
                ["结节回声特点：", ["低回声"]],
                ["结节形态特点：", ["规则形态"]],
                ["结节大小：", ["约 2.2cm*1.3cm*1.6cm"]],
                ["结节纵横比：", ["纵横比<1"]],
                ["结节边界特点：", ["欠清晰边界"]],
                ["内部回声是否均匀：", ["欠均匀"]],
                ["后方回声特点：", ["无明显变化"]],
                ["CDFI：", ["结节内部及周边可见少量血流信号"]],
```

```
                ["余实质回声：", ["尚均匀"]]
            ]
        ],
        ["周围淋巴结：", ["未探及明显增大淋巴结"]]
    ]
]

report_9 = [
    "甲状腺术后特点：",
    [
        ["局部特点：", ["未探及明显异常回声"]],
        ["颈部淋巴结：", ["未探及明显增大淋巴结"]],
        ["CDFI：", ["未见异常血流信号"]]
    ]
]
report_8 = [
"Features of bilateral thyroid lobes and isthmus:",
[
["Size and shape:", ["Normal volume", "Fairly regular shape"]],
["Features of the nodule in the left lobe:",
[
["Echo feature of the nodule:", ["Hypoechoic"]],
["Morphological feature of the nodule:", ["Regular shape"]],
["Size of the nodule:", ["Approx. 2.5cm1.5cm1.8cm"]],
["Nodule aspect ratio:", ["Aspect ratio < 1"]],
["Boundary feature of the nodule:", ["Clear boundary"]],
["Uniformity of internal echo:", ["Slightly heterogeneous"]],
["Posterior echo feature:", ["No obvious change"]],
["CDFI:", ["Slight blood flow signals can be seen in the solid part of the nodule"]]
]
],
["Features of the nodule in the right lobe:",
[
["Echo feature of the nodule:", ["Hypoechoic"]],
["Morphological feature of the nodule:", ["Regular shape"]],
["Size of the nodule:", ["Approx. 2.2cm1.3cm1.6cm"]],
["Nodule aspect ratio:", ["Aspect ratio < 1"]],
["Boundary feature of the nodule:", ["Slightly ill-defined boundary"]],
["Uniformity of internal echo:", ["Slightly heterogeneous"]],
["Posterior echo feature:", ["No obvious change"]],
["CDFI:", ["Slight blood flow signals can be seen inside and around the nodule"]],
["Echo of the remaining parenchyma:", ["Fairly homogeneous"]]
]
],
```

```
["Peripheral lymph nodes:", ["No obviously enlarged lymph nodes detected"]]
]
]
report_10 = [
    "甲状腺双侧叶及峡部特点：",
    [
        ["大小形态：", ["体积正常", "形态尚可"]],
        ["左叶结节特点：",
            [
                ["结节回声特点：", ["低回声"]],
                ["结节形态特点：", ["欠规则形态"]],
                ["结节大小：", ["约 1.3cm*0.6cm"]],
                ["结节纵横比：", ["纵横比<1"]],
                ["结节边界特点：", ["清晰边界"]],
                ["内部回声是否均匀：", ["欠均匀"]],
                ["有无微钙化：", ["无数据"]],
                ["有无点状强回声：", ["无数据"]],
                ["后方回声特点：", ["无明显变化"]],
                ["CDFI：", ["结节内可见少量血流信号"]]
            ]
        ],
        ["左叶结节特点：",
            [
                ["结节回声特点：", ["极低回声"]],
                ["结节大小：", ["约 0.4cm*0.3cm"]],
                ["结节纵横比：", ["纵横比<1"]],
                ["结节边界特点：", ["清晰边界"]],
                ["内部回声是否均匀：", ["欠均匀"]],
                ["后方回声特点：", ["无明显变化"]],
                ["CDFI：", ["结节内未见明显血流信号"]]
            ]
        ],
        ["右叶结节特点：",
            [
                ["结节回声特点：", ["低回声"]],
                ["结节大小：", ["约 1.0cm*0.6cm"]],
                ["结节纵横比：", ["纵横比<1"]],
                ["结节边界特点：", ["清晰边界"]],
                ["内部回声是否均匀：", ["欠均匀"]],
                ["有无微钙化：", ["无数据"]],
                ["有无点状强回声：", ["无数据"]],
                ["后方回声特点：", ["无明显变化"]],
                ["CDFI：", ["结节内可见少量血流信号"]]
            ]
```

```
            ],
            ["峡部结节特点：",
                [
                    ["结节回声特点：", ["低回声"]],
                    ["结节大小：", ["约 1.5cm*0.5cm"]],
                    ["结节纵横比：", ["纵横比<1"]],
                    ["结节边界特点：", ["清晰边界"]],
                    ["内部回声是否均匀：", ["欠均匀"]],
                    ["后方回声特点：", ["无明显变化"]],
                    ["CDFI：", ["结节内部及周边可见较丰富血流信号"]],
                    ["余腺体特点：", ["血流信号较丰富"]]
                ]
            ],
            ["周围淋巴结：", ["未探及明显增大淋巴结"]]
        ]
]
report_10 = [
"Characteristics of bilateral thyroid lobes and isthmus:",
[
["Size and shape:", ["Normal volume", "Fairly regular shape"]],
["Characteristics of nodules in the left lobe:",
[
["Nodule echogenicity:", ["Hypoechoic"]],
["Nodule morphology:", ["Irregular shape"]],
["Nodule size:", ["Approx. 1.3cm0.6cm"]],
["Nodule aspect ratio:", ["Aspect ratio < 1"]],
["Nodule boundary:", ["Clear boundary"]],
["Homogeneity of internal echo:", ["Slightly heterogeneous"]],
["Presence of microcalcifications:", ["No data available"]],
["Presence of punctate hyperechoic foci:", ["No data available"]],
["Posterior echo feature:", ["No significant change"]],
["CDFI:", ["Sparse blood flow signals visible within the nodule"]]
]
],
["Characteristics of nodules in the left lobe:",
[
["Nodule echogenicity:", ["Markedly hypoechoic"]],
["Nodule size:", ["Approx. 0.4cm0.3cm"]],
["Nodule aspect ratio:", ["Aspect ratio < 1"]],
["Nodule boundary:", ["Clear boundary"]],
["Homogeneity of internal echo:", ["Slightly heterogeneous"]],
["Posterior echo feature:", ["No significant change"]],
["CDFI:", ["No obvious blood flow signals detected within the nodule"]]
]
```

```
],
["Characteristics of nodules in the right lobe:",
[
["Nodule echogenicity:", ["Hypoechoic"]],
["Nodule size:", ["Approx. 1.0cm0.6cm"]],
["Nodule aspect ratio:", ["Aspect ratio < 1"]],
["Nodule boundary:", ["Clear boundary"]],
["Homogeneity of internal echo:", ["Slightly heterogeneous"]],
["Presence of microcalcifications:", ["No data available"]],
["Presence of punctate hyperechoic foci:", ["No data available"]],
["Posterior echo feature:", ["No significant change"]],
["CDFI:", ["Sparse blood flow signals visible within the nodule"]]
]
],
["Characteristics of nodules in the isthmus:",
[
["Nodule echogenicity:", ["Hypoechoic"]],
["Nodule size:", ["Approx. 1.5cm0.5cm"]],
["Nodule aspect ratio:", ["Aspect ratio < 1"]],
["Nodule boundary:", ["Clear boundary"]],
["Homogeneity of internal echo:", ["Slightly heterogeneous"]],
["Posterior echo feature:", ["No significant change"]],
["CDFI:", ["Abundant blood flow signals visible inside and around the nodule"]],
["Features of residual gland tissue:", ["Relatively abundant blood flow signals"]]
]
],
["Peripheral lymph nodes:", ["No obviously enlarged lymph nodes detected"]]
]
]
report_11 = [
    "左叶结节特点：",
    [
        ["结节回声特点：",["无数据"]],
        ["结节形态特点：",["无数据"]],
        ["结节大小：",["无数据"]],
        ["结节纵横比：",["无数据"]],
        ["结节边界特点：",["无数据"]],
        ["内部回声是否均匀：",["无数据"]],
        ["有无微钙化：",["无数据"]],
        ["有无点状强回声：",["无数据"]],
        ["后方回声特点：",["无数据"]]
    ],
    "右叶结节特点：",
    [
```

```
            ["结节回声特点：",["低回声"]],
            ["结节形态特点：",["规则形态"]],
            ["结节大小：",["约 0.3cm*0.2cm"]],
            ["结节纵横比：",["纵横比>1"]],
            ["结节边界特点：",["清晰边界"]],
            ["内部回声是否均匀：",["欠均匀"]],
            ["有无微钙化：",["无数据"]],
            ["有无点状强回声：",["有点状强回声伴彗星尾征"]],
            ["后方回声特点：",["无明显变化"]]
        ]
]
report_11 = [
"Characteristics of left lobe nodules:",
[
["Nodule echogenicity characteristics:",["No data"]],
["Nodule morphological characteristics:",["No data"]],
["Nodule size:",["No data"]],
["Nodule anteroposterior-transverse ratio:",["No data"]],
["Nodule boundary characteristics:",["No data"]],
["Homogeneity of internal echo:",["No data"]],
["Presence of microcalcifications:",["No data"]],
["Presence of punctate strong echoes:",["No data"]],
["Posterior echo characteristics:",["No data"]]
],
"Characteristics of right lobe nodules:",
[
["Nodule echogenicity characteristics:",["Hypoechoic"]],
["Nodule morphological characteristics:",["Regular shape"]],
["Nodule size:",["Approx. 0.3cm*0.2cm"]],
["Nodule anteroposterior-transverse ratio:",["Ratio > 1"]],
["Nodule boundary characteristics:",["Clear boundary"]],
["Homogeneity of internal echo:",["Slightly heterogeneous"]],
["Presence of microcalcifications:",["No data"]],
["Presence of punctate strong echoes:",["Punctate strong echoes with comet-tail artifact"]],
["Posterior echo characteristics:",["No significant change"]]
]
]
report_12 = [
        "左叶结节特点：",
        [
            ["结节回声特点：",["低回声"]],
            ["结节形态特点：",["规则形态"]],
            ["结节大小：",["约 0.6cm*0.4cm"]],
            ["结节纵横比：",["纵横比<1"]],
```

```
        ["结节边界特点：",["清晰边界"]],
        ["内部回声是否均匀：",["欠均匀"]],
        ["有无微钙化：",["无数据"]],
        ["有无点状强回声：",["无数据"]],
        ["后方回声特点：",["无明显变化"]]
    ],
    [
        ["结节回声特点：",["低回声"]],
        ["结节形态特点：",["欠规则形态"]],
        ["结节大小：",["约 0.5cm*0.4cm"]],
        ["结节纵横比：",["纵横比<1"]],
        ["结节边界特点：",["欠清晰边界，边缘毛刺"]],
        ["内部回声是否均匀：",["不均匀"]],
        ["有无微钙化：",["粗大钙化"]],
        ["有无点状强回声：",["无数据"]],
        ["后方回声特点：",["伴声影"]]
    ],
    "右叶结节特点：",
    [
        ["结节回声特点：",["低回声"]],
        ["结节形态特点：",["规则形态"]],
        ["结节大小：",["约 0.4cm*0.3cm"]],
        ["结节纵横比：",["纵横比<1"]],
        ["结节边界特点：",["清晰边界"]],
        ["内部回声是否均匀：",["欠均匀"]],
        ["有无微钙化：",["无数据"]],
        ["有无点状强回声：",["无数据"]],
        ["后方回声特点：",["无明显变化"]]
    ]
]
report_12 = [
"Characteristics of left lobe nodules:",
[
["Nodule echogenicity:",["Hypoechoic"]],
["Nodule shape:",["Regular shape"]],
["Nodule size:",["Approx. 0.6cm0.4cm"]],
["Nodule aspect ratio:",["Aspect ratio < 1"]],
["Nodule margin:",["Clear margin"]],
["Homogeneity of internal echo:",["Slightly heterogeneous"]],
["Presence of microcalcifications:",["No data"]],
["Presence of punctate hyperechoic foci:",["No data"]],
["Posterior echo features:",["No obvious change"]]
],
[
```

```
["Nodule echogenicity:",["Hypoechoic"]],
["Nodule shape:",["Slightly irregular shape"]],
["Nodule size:",["Approx. 0.5cm0.4cm"]],
["Nodule aspect ratio:",["Aspect ratio < 1"]],
["Nodule margin:",["Slightly blurred margin with spiculated edges"]],
["Homogeneity of internal echo:",["Heterogeneous"]],
["Presence of microcalcifications:",["Coarse calcifications"]],
["Presence of punctate hyperechoic foci:",["No data"]],
["Posterior echo features:",["With acoustic shadow"]]
],
"Characteristics of right lobe nodules:",
[
["Nodule echogenicity:",["Hypoechoic"]],
["Nodule shape:",["Regular shape"]],
["Nodule size:",["Approx. 0.4cm*0.3cm"]],
["Nodule aspect ratio:",["Aspect ratio < 1"]],
["Nodule margin:",["Clear margin"]],
["Homogeneity of internal echo:",["Slightly heterogeneous"]],
["Presence of microcalcifications:",["No data"]],
["Presence of punctate hyperechoic foci:",["No data"]],
["Posterior echo features:",["No obvious change"]]
]
]
report_13 = [
    "左叶结节特点：",
    [
        ["结节回声特点：",["无数据"]],
        ["结节形态特点：",["无数据"]],
        ["结节大小：",["无数据"]],
        ["结节纵横比：",["无数据"]],
        ["结节边界特点：",["无数据"]],
        ["内部回声是否均匀：",["无数据"]],
        ["有无微钙化：",["无数据"]],
        ["有无点状强回声：",["无数据"]],
        ["后方回声特点：",["无数据"]]
    ],
    "右叶结节特点：",
    [
        ["结节回声特点：",["无数据"]],
        ["结节形态特点：",["无数据"]],
        ["结节大小：",["无数据"]],
        ["结节纵横比：",["无数据"]],
        ["结节边界特点：",["无数据"]],
        ["内部回声是否均匀：",["无数据"]],
```

```
        ["有无微钙化：",["无数据"]],
        ["有无点状强回声：",["无数据"]],
        ["后方回声特点：",["无数据"]]
    ]
]
report_13 = [
"Characteristics of left lobe nodules:",
[
["Nodule echogenicity characteristics:",["No data"]],
["Nodule morphological characteristics:",["No data"]],
["Nodule size:",["No data"]],
["Nodule anteroposterior-transverse ratio:",["No data"]],
["Nodule boundary characteristics:",["No data"]],
["Homogeneity of internal echo:",["No data"]],
["Presence of microcalcifications:",["No data"]],
["Presence of punctate strong echoes:",["No data"]],
["Posterior echo characteristics:",["No data"]]
],
"Characteristics of right lobe nodules:",
[
["Nodule echogenicity characteristics:",["No data"]],
["Nodule morphological characteristics:",["No data"]],
["Nodule size:",["No data"]],
["Nodule anteroposterior-transverse ratio:",["No data"]],
["Nodule boundary characteristics:",["No data"]],
["Homogeneity of internal echo:",["No data"]],
["Presence of microcalcifications:",["No data"]],
["Presence of punctate strong echoes:",["No data"]],
["Posterior echo characteristics:",["No data"]]
]
]
report_14 = [
    "左叶结节特点：",
    [
        ["结节回声特点：",["低回声"]],
        ["结节形态特点：",["规则形态"]],
        ["结节大小：",["约 3.8cm*2.5cm*2.3cm"]],
        ["结节纵横比：",["无数据"]],
        ["结节边界特点：",["欠清晰边界"]],
        ["内部回声是否均匀：",["欠均匀"]],
        ["有无微钙化：",["簇状钙化"]],
        ["有无点状强回声：",["无数据"]],
        ["后方回声特点：",["无数据"]]
    ],
```

```
        "右叶结节特点：",
        [
            ["结节回声特点：",["无数据"]],
            ["结节形态特点：",["无数据"]],
            ["结节大小：",["无数据"]],
            ["结节纵横比：",["无数据"]],
            ["结节边界特点：",["无数据"]],
            ["内部回声是否均匀：",["无数据"]],
            ["有无微钙化：",["无数据"]],
            ["有无点状强回声：",["无数据"]],
            ["后方回声特点：",["无数据"]]
        ]
]
report_14 = [
"Characteristics of Left Lobe Nodules:",
[
["Nodule Echogenicity:",["Hypoechoic"]],
["Nodule Morphology:",["Regular shape"]],
["Nodule Size:",["Approx. 3.8cm2.5cm2.3cm"]],
["Nodule Aspect Ratio:",["No data"]],
["Nodule Margin:",["Indistinct margin"]],
["Homogeneity of Internal Echo:",["Slightly heterogeneous"]],
["Presence of Microcalcifications:",["Clustered calcifications"]],
["Presence of Punctate Hyperechoic Foci:",["No data"]],
["Posterior Echo Feature:",["No data"]]
],
"Characteristics of Right Lobe Nodules:",
[
["Nodule Echogenicity:",["No data"]],
["Nodule Morphology:",["No data"]],
["Nodule Size:",["No data"]],
["Nodule Aspect Ratio:",["No data"]],
["Nodule Margin:",["No data"]],
["Homogeneity of Internal Echo:",["No data"]],
["Presence of Microcalcifications:",["No data"]],
["Presence of Punctate Hyperechoic Foci:",["No data"]],
["Posterior Echo Feature:",["No data"]]
]
]
report_15 = [
        "左叶结节特点：",
        [
            ["结节回声特点：",["无数据"]],
            ["结节形态特点：",["无数据"]],
```

```
        ["结节大小：",["无数据"]],
        ["结节纵横比：",["无数据"]],
        ["结节边界特点：",["无数据"]],
        ["内部回声是否均匀：",["无数据"]],
        ["有无微钙化：",["无数据"]],
        ["有无点状强回声：",["无数据"]],
        ["后方回声特点：",["无数据"]]
    ],
    "右叶结节特点：",
    [
        ["结节回声特点：",["低回声"]],
        ["结节形态特点：",["欠规则形态"]],
        ["结节大小：",["约 1.5cm*0.6cm"]],
        ["结节纵横比：",["纵横比<1"]],
        ["结节边界特点：",["欠清晰边界"]],
        ["内部回声是否均匀：",["欠均匀"]],
        ["有无微钙化：",["无数据"]],
        ["有无点状强回声：",["无数据"]],
        ["后方回声特点：",["无明显变化"]]
    ]
]
report_15 = [
"Characteristics of Left Lobe Nodules:",
[
["Nodule Echogenicity Characteristics:",["No data"]],
["Nodule Morphological Characteristics:",["No data"]],
["Nodule Size:",["No data"]],
["Nodule Aspect Ratio:",["No data"]],
["Nodule Boundary Characteristics:",["No data"]],
["Homogeneity of Internal Echo:",["No data"]],
["Presence of Microcalcifications:",["No data"]],
["Presence of Punctate Hyperechoic Foci:",["No data"]],
["Posterior Echo Characteristics:",["No data"]]
],
"Characteristics of Right Lobe Nodules:",
[
["Nodule Echogenicity Characteristics:",["Hypoechoic"]],
["Nodule Morphological Characteristics:",["Irregular shape"]],
["Nodule Size:",["Approx. 1.5cm*0.6cm"]],
["Nodule Aspect Ratio:",["Aspect ratio < 1"]],
["Nodule Boundary Characteristics:",["Indistinct boundary"]],
["Homogeneity of Internal Echo:",["Heterogeneous"]],
["Presence of Microcalcifications:",["No data"]],
["Presence of Punctate Hyperechoic Foci:",["No data"]],
```

```
["Posterior Echo Characteristics:",["No significant changes"]]
]
]
report_16 = [
    "左叶结节特点：",
    [
        ["结节回声特点：",["无数据"]],
        ["结节形态特点：",["无数据"]],
        ["结节大小：",["无数据"]],
        ["结节纵横比：",["无数据"]],
        ["结节边界特点：",["无数据"]],
        ["内部回声是否均匀：",["无数据"]],
        ["有无微钙化：",["无数据"]],
        ["有无点状强回声：",["无数据"]],
        ["后方回声特点：",["无数据"]]
    ],
    "右叶结节特点：",
    [
        ["结节回声特点：",["低回声"]],
        ["结节形态特点：",["规则形态"]],
        ["结节大小：",["约 0.9cm*0.6cm"]],
        ["结节纵横比：",["纵横比<1"]],
        ["结节边界特点：",["清晰边界"]],
        ["内部回声是否均匀：",["欠均匀"]],
        ["有无微钙化：",["无数据"]],
        ["有无点状强回声：",["无数据"]],
        ["后方回声特点：",["无明显变化"]]
    ]
]
report_16 = [
"Characteristics of left lobe nodules:",
[
["Nodule echogenicity characteristics:",["No data"]],
["Nodule morphological characteristics:",["No data"]],
["Nodule size:",["No data"]],
["Nodule aspect ratio:",["No data"]],
["Nodule boundary characteristics:",["No data"]],
["Homogeneity of internal echo:",["No data"]],
["Presence of microcalcifications:",["No data"]],
["Presence of punctate strong echoes:",["No data"]],
["Posterior echo characteristics:",["No data"]]
],
"Characteristics of right lobe nodules:",
[
```

```
["Nodule echogenicity characteristics:",["Hypoechoic"]],
["Nodule morphological characteristics:",["Regular shape"]],
["Nodule size:",["Approx. 0.9cm*0.6cm"]],
["Nodule aspect ratio:",["Aspect ratio < 1"]],
["Nodule boundary characteristics:",["Clear boundary"]],
["Homogeneity of internal echo:",["Slightly inhomogeneous"]],
["Presence of microcalcifications:",["No data"]],
["Presence of punctate strong echoes:",["No data"]],
["Posterior echo characteristics:",["No obvious changes"]]
]
report_17 = [
    "左叶结节特点：",
    [
        ["结节回声特点：",["低回声"]],
        ["结节形态特点：",["规则形态"]],
        ["结节大小：",["约 0.8cm*0.5cm"]],
        ["结节纵横比：",["纵横比<1"]],
        ["结节边界特点：",["清晰边界"]],
        ["内部回声是否均匀：",["欠均匀"]],
        ["有无微钙化：",["无数据"]],
        ["有无点状强回声：",["无数据"]],
        ["后方回声特点：",["无明显变化"]]
    ],
    "右叶结节特点：",
    [
        ["结节回声特点：",["低回声"]],
        ["结节形态特点：",["规则形态"]],
        ["结节大小：",["约 2.0cm*1.2cm"]],
        ["结节纵横比：",["纵横比<1"]],
        ["结节边界特点：",["清晰边界"]],
        ["内部回声是否均匀：",["欠均匀"]],
        ["有无微钙化：",["无数据"]],
        ["有无点状强回声：",["无数据"]],
        ["后方回声特点：",["无明显变化"]]
    ],
    [
        ["结节回声特点：",["低回声"]],
        ["结节形态特点：",["欠规则形态"]],
        ["结节大小：",["约 0.4cm*0.6cm*0.7cm"]],
        ["结节纵横比：",["纵横比>1"]],
        ["结节边界特点：",["欠清晰边界，边缘毛刺"]],
        ["内部回声是否均匀：",["欠均匀"]],
        ["有无微钙化：",["点状强回声"]],
        ["有无点状强回声：",["无数据"]],
```

```
            ["后方回声特点：",["无明显变化"]]
        ]
]
report_17 = [
"Characteristics of Left Lobe Nodules:",
[
["Nodule Echogenicity:",["Hypoechoic"]],
["Nodule Morphology:",["Regular shape"]],
["Nodule Size:",["Approx. 0.8cm0.5cm"]],
["Nodule Aspect Ratio:",["Aspect ratio < 1"]],
["Nodule Margin:",["Clear margin"]],
["Homogeneity of Internal Echo:",["Slightly heterogeneous"]],
["Presence of Microcalcifications:",["No data"]],
["Presence of Punctate Hyperechoic Foci:",["No data"]],
["Posterior Echo Feature:",["No significant change"]]
],
"Characteristics of Right Lobe Nodules:",
[
["Nodule Echogenicity:",["Hypoechoic"]],
["Nodule Morphology:",["Regular shape"]],
["Nodule Size:",["Approx. 2.0cm1.2cm"]],
["Nodule Aspect Ratio:",["Aspect ratio < 1"]],
["Nodule Margin:",["Clear margin"]],
["Homogeneity of Internal Echo:",["Slightly heterogeneous"]],
["Presence of Microcalcifications:",["No data"]],
["Presence of Punctate Hyperechoic Foci:",["No data"]],
["Posterior Echo Feature:",["No significant change"]]
],
[
["Nodule Echogenicity:",["Hypoechoic"]],
["Nodule Morphology:",["Slightly irregular shape"]],
["Nodule Size:",["Approx. 0.4cm*0.6cm*0.7cm"]],
["Nodule Aspect Ratio:",["Aspect ratio > 1"]],
["Nodule Margin:",["Slightly ill-defined margin with spiculated edges"]],
["Homogeneity of Internal Echo:",["Slightly heterogeneous"]],
["Presence of Microcalcifications:",["Punctate hyperechoic foci"]],
["Presence of Punctate Hyperechoic Foci:",["No data"]],
["Posterior Echo Feature:",["No significant change"]]
]
]
report_18 = [
    "左叶结节特点：",
    [
        ["结节回声特点：",["低回声"]],
```

```
		["结节形态特点：",["规则形态"]],
		["结节大小：",["约 0.3cm*0.2cm"]],
		["结节纵横比：",["纵横比<1"]],
		["结节边界特点：",["清晰边界"]],
		["内部回声是否均匀：",["欠均匀"]],
		["有无微钙化：",["无数据"]],
		["有无点状强回声：",["无数据"]],
		["后方回声特点：",["无明显变化"]]
	],
	"右叶结节特点：",
	[
		["结节回声特点：",["低回声"]],
		["结节形态特点：",["欠规则形态"]],
		["结节大小：",["约 0.8cm*0.6cm"]],
		["结节纵横比：",["纵横比<1"]],
		["结节边界特点：",["尚清晰边界"]],
		["内部回声是否均匀：",["欠均匀"]],
		["有无微钙化：",["无数据"]],
		["有无点状强回声：",["无数据"]],
		["后方回声特点：",["无明显变化"]]
	]
]
report_18 = [
"Characteristics of left lobe nodules:",
[
["Nodule echogenicity: ",["Hypoechoic"]],
["Nodule morphology: ",["Regular shape"]],
["Nodule size: ",["Approx. 0.3cm0.2cm"]],
["Nodule aspect ratio: ",["Aspect ratio < 1"]],
["Nodule margin: ",["Well-defined margin"]],
["Homogeneity of internal echo: ",["Slightly heterogeneous"]],
["Presence of microcalcifications: ",["No data"]],
["Presence of punctate strong echoes: ",["No data"]],
["Posterior echo feature: ",["No significant change"]]
],
"Characteristics of right lobe nodules:",
[
["Nodule echogenicity: ",["Hypoechoic"]],
["Nodule morphology: ",["Slightly irregular shape"]],
["Nodule size: ",["Approx. 0.8cm0.6cm"]],
["Nodule aspect ratio: ",["Aspect ratio < 1"]],
["Nodule margin: ",["Relatively well-defined margin"]],
["Homogeneity of internal echo: ",["Slightly heterogeneous"]],
["Presence of microcalcifications: ",["No data"]],
```

```
["Presence of punctate strong echoes: ",["No data"]],
["Posterior echo feature: ",["No significant change"]]
]
]
report_19 = [
	"左叶结节特点：",
	[
		["结节回声特点：",["低回声"]],
		["结节形态特点：",["规则形态"]],
		["结节大小：",["约 0.9cm*0.6cm"]],
		["结节纵横比：",["纵横比<1"]],
		["结节边界特点：",["清晰边界"]],
		["内部回声是否均匀：",["欠均匀"]],
		["有无微钙化：",["无数据"]],
		["有无点状强回声：",["无数据"]],
		["后方回声特点：",["无明显变化"]]
	],
	"右叶结节特点：",
	[
		["结节回声特点：",["低回声"]],
		["结节形态特点：",["规则形态"]],
		["结节大小：",["约 0.7cm*0.5cm"]],
		["结节纵横比：",["纵横比<1"]],
		["结节边界特点：",["清晰边界"]],
		["内部回声是否均匀：",["欠均匀"]],
		["有无微钙化：",["无数据"]],
		["有无点状强回声：",["斑片状强回声"]],
		["后方回声特点：",["无明显变化"]]
	]
]
report_19 = [
"Characteristics of left lobe nodule:",
[
["Nodule echogenicity characteristics:",["Hypoechoic"]],
["Nodule morphological characteristics:",["Regular shape"]],
["Nodule size:",["Approx. 0.9cm0.6cm"]],
["Nodule aspect ratio:",["Aspect ratio < 1"]],
["Nodule boundary characteristics:",["Clear boundary"]],
["Uniformity of internal echo:",["Slightly heterogeneous"]],
["Presence of microcalcifications:",["No data"]],
["Presence of punctate hyperechoic foci:",["No data"]],
["Posterior echo characteristics:",["No significant changes"]]
],
"Characteristics of right lobe nodule:",
```

```
[
["Nodule echogenicity characteristics:",["Hypoechoic"]],
["Nodule morphological characteristics:",["Regular shape"]],
["Nodule size:",["Approx. 0.7cm0.5cm"]],
["Nodule aspect ratio:",["Aspect ratio < 1"]],
["Nodule boundary characteristics:",["Clear boundary"]],
["Uniformity of internal echo:",["Slightly heterogeneous"]],
["Presence of microcalcifications:",["No data"]],
["Presence of punctate hyperechoic foci:",["Patchy hyperechoic foci"]],
["Posterior echo characteristics:",["No significant changes"]]
]
]
report_20 = [
	"左叶结节特点：",
	[
		["结节回声特点：",["低回声"]],
		["结节形态特点：",["规则形态"]],
		["结节大小：",["约 0.5cm*0.4cm"]],
		["结节纵横比：",["纵横比<1"]],
		["结节边界特点：",["清晰边界"]],
		["内部回声是否均匀：",["欠均匀"]],
		["有无微钙化：",["无数据"]],
		["有无点状强回声：",["无数据"]],
		["后方回声特点：",["无明显变化"]]
	],
	"右叶结节特点：",
	[
		["结节回声特点：",["稍强回声"]],
		["结节形态特点：",["规则形态"]],
		["结节大小：",["约 0.5cm*0.4cm"]],
		["结节纵横比：",["纵横比<1"]],
		["结节边界特点：",["清晰边界"]],
		["内部回声是否均匀：",["欠均匀"]],
		["有无微钙化：",["无数据"]],
		["有无点状强回声：",["无数据"]],
		["后方回声特点：",["无明显变化"]]
	]
]
report_20 = [
"Characteristics of left lobe nodule:",
[
["Nodule echogenicity feature:",["Hypoechoic"]],
["Nodule morphological feature:",["Regular shape"]],
["Nodule size:",["Approx. 0.5cm0.4cm"]],
```

["Nodule aspect ratio:",["Aspect ratio < 1"]],
["Nodule boundary feature:",["Clear boundary"]],
["Is internal echo uniform:",["Slightly uneven"]],
["Presence of microcalcification:",["No data"]],
["Presence of punctate hyperechoic foci:",["No data"]],
["Posterior echo feature:",["No obvious change"]]
],
"Characteristics of right lobe nodule:",
[
["Nodule echogenicity feature:",["Slightly hyperechoic"]],
["Nodule morphological feature:",["Regular shape"]],
["Nodule size:",["Approx. 0.5cm0.4cm"]],
["Nodule aspect ratio:",["Aspect ratio < 1"]],
["Nodule boundary feature:",["Clear boundary"]],
["Is internal echo uniform:",["Slightly uneven"]],
["Presence of microcalcification:",["No data"]],
["Presence of punctate hyperechoic foci:",["No data"]],
["Posterior echo feature:",["No obvious change"]]
]
]

3. Prompt Engineering (System and User Prompts)

3.提示词工程（系统提示词与用户提示词）

The following prompt template was utilized across all six LLMs to ensure standardized instruction following.

六款大语言模型均采用了下述提示词模板，以保证模型遵循指令的标准统一。

System Prompt:

系统提示词:

"Senior Thyroid Nodule Classification Assessor. You are an expert with profound attainments and abundant clinical experience in the field of medical imaging, especially thyroid ultrasonography. You possess thorough expertise in the principles of ultrasound and imaging characteristics of thyroid nodules, have a full grasp of fundamental principles such as sound wave propagation, reflection and scattering in different tissues, and are well-versed in the normal echo features of thyroid tissues on ultrasound images, as well as the underlying principles and clinical implications of various nodule echo types including hypoechoic, hyperechoic, isoechoic and markedly hypoechoic nodules. You have an accurate command of the C-TIRADS classification system and a clear understanding of the definition and corresponding malignancy risk of each category: C-TIRADS Category 1 refers to normal thyroid gland without nodules; C-TIRADS Category 2 stands for benign nodules; C-TIRADS Category 3 indicates probably benign nodules; C-TIRADS Category 4A denotes low suspicion of malignancy; C-TIRADS Category 4B denotes moderate suspicion of malignancy; C-TIRADS Category 4C denotes high suspicion of malignancy; C-TIRADS Category 5 is highly suggestive of malignancy; C-TIRADS Category 6 confirms malignancy via pathological examination. Meanwhile, you have a clear understanding of the correlations between each classification grade and nodule features (such as echo, morphology, anteroposterior-to-transverse diameter ratio, margin, internal echo homogeneity, calcification

status and blood flow signals):
Echo characteristics: Markedly hypoechoic appearance is frequently seen in malignant nodules; hypoechoic appearance elevates malignancy risk; isoechoic and hyperechoic appearances are more likely benign, yet special cases of hyperechoic nodules with microcalcifications require differentiated evaluation.
Morphology: Regular shapes such as round and oval are mostly associated with benign nodules, while irregular shapes like lobulated and spiculated ones imply an increased risk of malignancy.
Anteroposterior-to-transverse diameter ratio: A ratio greater than 1 serves as a key marker of malignant nodules, whereas a ratio less than 1 is common in benign nodules.
Margins: Well-defined margins generally indicate benignity, while indistinct or ill-defined margins suggest malignancy.
Internal echo homogeneity: Homogeneous echoes usually correspond to benign nodules, and heterogeneous echoes—especially nodules containing multiple distinct echo zones—carry higher malignancy risk.
Calcification status: Microcalcifications are strongly correlated with malignant nodules; coarse calcifications have a relatively higher likelihood of benignity but cannot completely rule out malignancy; punctate hyperechoic foci with comet-tail artifacts mostly represent benign colloid crystals.
Blood flow signals: Absent or scant blood flow signals are common in benign nodules, while abundant blood flow signals raise malignancy risk.
You are proficient in diagnostic criteria for thyroid nodules:
Nodule size: Generally speaking, larger nodules may carry higher malignancy risk, though this correlation is not absolute.
Echo features: Beyond the aforementioned correlations between echo types and benign/malignant nature, the overall echo distribution across the entire nodule must be taken into account.
Morphologic regularity: In addition to basic judgments of regular and irregular morphology, subtle morphologic changes such as local protrusions and indentations should be noted.
Anteroposterior-to-transverse diameter ratio: Precisely measure this ratio and conduct comprehensive assessment combined with other nodule features.
Margin status: Rather than simply distinguishing between clear and blurred margins, attention should be paid to margin smoothness and signs of infiltration.
Internal echo homogeneity: Evaluate the degree of internal echo uniformity and the distribution pattern of varying echo zones.
Calcification status: Differentiate the type, size and distribution of calcifications, as clustered calcifications and punctate calcifications carry distinct clinical implications.
Blood flow signals: Analyze the distribution pattern of blood flow signals, such as peripheral-dominant versus intranodular-dominant blood supply.
Workflow:
Carefully receive thyroid ultrasound report data and accurately extract key information, including nodule location, quantity, size, echo characteristics, morphology, anteroposterior-to-transverse diameter ratio, margins, internal echo homogeneity, calcification status, blood flow signals and status of surrounding lymph nodes.
Conduct in-depth analysis of the impact of each feature on nodule classification, synthesize all identified features, and perform standardized assessment in strict accordance with the rules of the

C-TIRADS classification system."
你是医学影像领域的专业专家，在甲状腺超声方向具备深厚学术造诣与丰富临床实操经验。你精通超声成像原理以及各类甲状腺结节的影像学特征，熟练掌握声波在不同人体组织内传播、反射、散射等基础声学原理；熟知甲状腺正常组织的超声回声表现，同时透彻掌握低回声、高回声、等回声、极低回声等各类结节回声分型的形成机理与对应的临床意义。
你能够精准运用 C-TIRADS 甲状腺结节分类标准，清晰掌握每一分级的定义及对应的恶性风险：
C-TIRADS 1 类：甲状腺腺体正常，无结节；
C-TIRADS 2 类：良性结节；
C-TIRADS 3 类：大概率良性结节；
C-TIRADS 4A 类：低度恶性可疑结节；
C-TIRADS 4B 类：中度恶性可疑结节；
C-TIRADS 4C 类：高度恶性可疑结节；
C-TIRADS 5 类：高度提示恶性结节；
C-TIRADS 6 类：经病理检查确诊恶性结节。
同时，你充分掌握各分级与结节影像学特征之间的对应关联，涵盖回声、形态、纵横比、边界、内部回声均匀度、钙化情况、血流信号等指标，具体判定标准如下：
一、回声特征
极低回声多见于恶性结节；低回声会提升恶性病变概率；等回声、高回声结节大多为良性，但合并微钙化的高回声结节需单独鉴别评估。
二、形态特征
圆形、椭圆形等规则形态结节多为良性；分叶状、毛刺状等不规则形态结节恶性风险更高。
三、纵横比
结节纵横比大于 1 是恶性结节的核心判断指标；纵横比小于 1 多见于良性结节。
四、边界特征
边界清晰规整多提示良性；边界模糊、分界不清提示恶性可能。
五、内部回声均匀度
回声均匀的结节一般为良性；回声不均匀，尤其是内部存在多种回声分区的结节，恶性风险显著升高。
六、钙化类型
微钙化与甲状腺恶性结节高度相关；粗大钙化良性概率相对更高，但无法完全排除恶性；伴彗尾征的点状强回声灶大多为良性胶质结晶。
七、血流信号
无血流或仅少量血流信号多见于良性结节；结节内部血流信号丰富时，恶性风险上升。
你熟练掌握甲状腺结节全套诊断评判要点：
结节大小：通常结节体积越大，恶性风险越高，但该关联并非绝对；
回声表现：除上述回声分型与良恶性的对应规律外，需综合判断结节整体回声分布状态；
形态规整度：除基础的规则 / 不规则判定外，需留意局部凸起、凹陷等细微形态改变；
纵横比：精准测算数值，并结合其余结节特征综合评判；
边界状态：不能仅简单区分清晰、模糊两类，需重点观察边界光滑程度以及有无浸润征象；
内部回声均匀度：评估内部回声均匀程度，以及不同回声区域的分布模式；
钙化情况：区分钙化的类型、大小与分布特点，簇状钙化、点状钙化分别对应不同临床提示意义；
血流信号：分辨血流分布模式，区分以周边供血为主、以结节内部供血为主两种类型。

工作流程
完整接收甲状腺超声报告数据，精准提取核心信息：结节位置、数量、大小、回声特征、形态、纵横比、边界、内部回声均匀度、钙化情况、血流信号，以及周边淋巴结状态；
逐项分析各项影像特征对结节分级判定的影响，整合全部影像学指标，严格遵循 C-TIRADS 分类体系规范完成分级评估。
Output prompt: Extract the locations of thyroid nodules from the ultrasound findings, and format the following content into a table with the headers | Report Serial Number | Location |. Describe multiple nodules at the same location separately. Fill in "Thyroid" if the specific location of a nodule is not specified, with one row for each location.
输出提示词：提取超声所见中甲状腺结节的位置，将下列内容按表格形式:|报告序号|位置|，同一位置的多个结节分开描述，未明确具体结节位置的填甲状腺，一个位置一行。
User Prompt (Example):
用户提示词（示例）：
"Based on the following features, determine the C-TIRADS grade,C-TIRADS 2020 Logic Embedded in Prompt."
根据以下特征判定 C-TIRADS 分级：提示词内置 2020 版 C-TIRADS 判定逻辑：
4. Model Version Identifiers and Hyperparameters
4.模型版本标识符与超参数
The self-hosted One API gateway, exclusively accessible via the local intranet address http://localhost:3001/, acted as a unified intermediate scheduling layer for all large language models. FastGPT acquired dedicated access tokens and configuration credentials automatically generated by this local One API gateway, and invoked all integrated models through the localhost endpoint.
For DeepSeek-V3, GLM-4 and all Qwen variants: their fully open-source complete weight files were downloaded and stored on isolated hospital servers. Offline inference was executed via the local One API gateway at http://localhost:3001, with all outbound public network traffic blocked on these servers to eliminate any risk of patient data leakage.
For Doubao-Pro-128k, ChatGPT-4o-Latest and GPT-3.5-Turbo: no open-source downloadable local weights are publicly available. Only official cloud API keys from corresponding vendors were configured inside the local One API gateway for intranet request forwarding. Comprehensive de-identification was applied to all clinical text prior to external transmission to strip every piece of patient identifiable information.
One API
Features
1. Support for multiple large models:
   + [x] [OpenAI ChatGPT Series Models](https://platform.openai.com/docs/guides/gpt/chat-completions-api) (Supports [Azure OpenAI API](https://learn.microsoft.com/en-us/azure/ai-services/openai/reference))
   + [x] [Anthropic Claude Series Models](https://anthropic.com)
   + [x] [Google PaLM2 and Gemini Series Models](https://developers.generativeai.google)
   + [x] [Baidu Wenxin Yiyuan Series Models](https://cloud.baidu.com/doc/WENXINWORKSHOP/index.html)
   + [x] [Alibaba Tongyi Qianwen Series Models](https://help.aliyun.com/document_detail/2400395.html)

+ [x] [Zhipu ChatGLM Series Models](https://bigmodel.cn)
2. Supports access to multiple channels through **load balancing**.
3. Supports **stream mode** that enables typewriter-like effect through stream transmission.
4. Supports **multi-machine deployment**. [See here](#multi-machine-deployment) for more details.
5. Supports **token management** that allows setting token expiration time and usage count.
6. Supports **voucher management** that enables batch generation and export of vouchers. Vouchers can be used for account balance replenishment.
7. Supports **channel management** that allows bulk creation of channels.
8. Supports **user grouping** and **channel grouping** for setting different rates for different groups.
9. Supports channel **model list configuration**.
10. Supports **quota details checking**.
11. Supports **user invite rewards**.
12. Allows display of balance in USD.
13. Supports announcement publishing, recharge link setting, and initial balance setting for new users.
14. Offers rich **customization** options:
    1. Supports customization of system name, logo, and footer.
    2. Supports customization of homepage and about page using HTML & Markdown code, or embedding a standalone webpage through iframe.
15. Supports management API access through system access tokens.
16. Supports Cloudflare Turnstile user verification.
17. Supports user management and multiple user login/registration methods:
    + Email login/registration and password reset via email.
    + [GitHub OAuth](https://github.com/settings/applications/new).
    + WeChat Official Account authorization (requires additional deployment of [WeChat Server](https://github.com/songquanpeng/wechat-server)).
18. Immediate support and encapsulation of other major model APIs as they become available.

#One API + FastGPT Local Large Model Deployment Process
#Deployment of One API
#Open-source repository address of the One API project:
https://github.com/songquanpeng/one-api
#Create a directory named oneapi under the /opt directory.
cd opt
mkdir oneapi
cd oneapi
#Create a file named docker-compose.yml under the /opt/oneapi directory, paste the following content into the file and save it.
#Edit the docker-compose.yml configuration file.

```
version: '3.8'
services:
  oneapi:
    container_name: oneapi
```

```
    image: justsong/one-api:0.6.11
    restart: unless-stopped
    ports:
      - 3001:3000
    networks:
      - fastgpt
    volumes:
      - ./data:/data
    environment:
      - TZ=Asia/Shanghai
networks:
  fastgpt:
    name: fastgpt
    external: true
#Create a Docker network named fastgpt.
docker network create fastgpt
#Start the One API service via docker-compose.
docker compose up -d
#Log in to the One API backend at http://localhost:3001 with the default username root and password 123456.
#After successfully adding a channel, click the Test button on the channel list page. A success response will be returned if the configuration works properly.
```

ID	名称	分组	类型	状态	响应时间	优先级	操作
24	deepseek	default	DeepSeek	已启用	3.34 秒	0	测试 删除 禁用 编辑
23	ChatGPT-4o-Latest	default	OpenAI	已启用	未测试	0	测试 删除 禁用 编辑
22	doubao-lite-32k	default	字节跳动豆包	已启用	1.99 秒	0	测试 删除 禁用 编辑
21	Doubao-lite-128k	default	字节跳动豆包	已启用	0.17 秒	0	测试 删除 禁用 编辑
20	shaw/dmeta-embedding-zh	default	Ollama	已启用	未测试	0	测试 删除 禁用 编辑
19	qwen2.5:14b	default	Ollama	已启用	6.78 秒	0	测试 删除 禁用 编辑
18	qwen2.5:7b	default	Ollama	已启用	1.96 秒	0	测试 删除 禁用 编辑
14	m3e	default	Ollama	已启用	未测试	0	测试 删除 禁用 编辑
13	doubao-pro-128k	default	字节跳动豆包	已启用	0.36 秒	0	测试 删除 禁用 编辑
10	glm-4-long	default	智谱 ChatGLM	已启用	1.55 秒	0	测试 删除 禁用 编辑

The screenshot displays the full channel list of the gateway. Only Doubao-pro-128k was activated for comparative experiments in this study; all lite variants were not included in the tests.

Config.json

```
{
  "feConfigs": {
    "lafEnv": "https://laf.dev"
  },
```

"systemEnv": {
  "vectorMaxProcess": 15,
  "qaMaxProcess": 15,
  "pgHNSWEfSearch": 100
},
"llmModels": [
  {
    "model": "deepseek-v3",
    "name": "DeepSeek-V3",
    "avatar": "/imgs/model/openai.svg",
    "maxContext": 64000,
    "maxResponse": 8000,
    "quoteMaxToken": 13000,
    "maxTemperature": 1.0,
    "charsPointsPrice": 0,
    "censor": false,
    "vision": false,
    "datasetProcess": true,
    "usedInClassify": true,
    "usedInExtractFields": true,
    "usedInToolCall": true,
    "usedInQueryExtension": true,
    "toolChoice": true,
    "functionCall": true,
    "customCQPrompt": "",
    "customExtractPrompt": "",
    "defaultSystemChatPrompt": "",
    "defaultConfig": {}
  },
  {
    "model": "glm-4-long",
    "name": "GLM-4-plus",
    "avatar": "/imgs/model/openai.svg",
    "maxContext": 128000,
    "maxResponse": 8000,
    "quoteMaxToken": 12000,
    "maxTemperature": 1.0,
    "charsPointsPrice": 0,
    "censor": false,
    "vision": false,
    "datasetProcess": true,
    "usedInClassify": true,
    "usedInExtractFields": true,
    "usedInToolCall": true,

"usedInQueryExtension": true,
"toolChoice": true,
"functionCall": true,
"customCQPrompt": "",
"customExtractPrompt": "",
"defaultSystemChatPrompt": "",
"defaultConfig": {}
},
{
"model": "ep-20240924204235-vx7hz",
"name": "Doubao-pro-128k",
"avatar": "/imgs/model/openai.svg",
"maxContext": 128000,
"maxResponse": 16000,
"quoteMaxToken": 12000,
"maxTemperature": 1.0,
"charsPointsPrice": 0,
"censor": false,
"vision": false,
"datasetProcess": true,
"usedInClassify": true,
"usedInExtractFields": true,
"usedInToolCall": true,
"usedInQueryExtension": true,
"toolChoice": true,
"functionCall": true,
"customCQPrompt": "",
"customExtractPrompt": "",
"defaultSystemChatPrompt": {},
"defaultConfig": {}
},
{
"model": "qwen-max-longcontext",
"name": "Qwen-plus",
"avatar": "/imgs/model/openai.svg",
"maxContext": 128000,
"maxResponse": 8000,
"quoteMaxToken": 12000,
"maxTemperature": 1.0,
"charsPointsPrice": 0,
"censor": false,
"vision": false,
"datasetProcess": true,
"usedInClassify": true,

```
    "usedInExtractFields": true,
    "usedInToolCall": true,
    "usedInQueryExtension": true,
    "toolChoice": true,
    "functionCall": true,
    "customCQPrompt": "",
    "customExtractPrompt": "",
    "defaultSystemChatPrompt": "",
    "defaultConfig": {}
  },
  {
    "model": "qwen2.5:7b",
    "name": "qwen2.5:7b",
    "maxContext": 32000,
    "avatar": "/imgs/model/openai.svg",
    "maxResponse": 8000,
    "quoteMaxToken": 20000,
    "maxTemperature": 1.0,
    "charsPointsPrice": 0,
    "censor": false,
    "vision": false,
    "datasetProcess": true,
    "usedInClassify": true,
    "usedInExtractFields": true,
    "usedInToolCall": true,
    "usedInQueryExtension": true,
    "toolChoice": true,
    "functionCall": true,
    "customCQPrompt": "",
    "customExtractPrompt": "",
    "defaultSystemChatPrompt": "",
    "defaultConfig": {}
  },
  {
    "model": "qwen2.5:14b",
    "name": "qwen2.5:14b",
    "maxContext": 32000,
    "avatar": "/imgs/model/openai.svg",
    "maxResponse": 8000,
    "quoteMaxToken": 20000,
    "maxTemperature": 1.0,
    "charsPointsPrice": 0,
    "censor": false,
    "vision": false,
```

"datasetProcess": true,
"usedInClassify": true,
"usedInExtractFields": true,
"usedInToolCall": true,
"usedInQueryExtension": true,
"toolChoice": true,
"functionCall": true,
"customCQPrompt": "",
"customExtractPrompt": "",
"defaultSystemChatPrompt": "",
"defaultConfig": {}
},
{
"model": "gpt-3.5-turbo",
"name": "GPT-3.5-Turbo",
"avatar": "/imgs/model/openai.svg",
"maxContext": 16384,
"maxResponse": 8000,
"quoteMaxToken": 12000,
"maxTemperature": 1.0,
"charsPointsPrice": 0,
"censor": false,
"vision": false,
"datasetProcess": true,
"usedInClassify": true,
"usedInExtractFields": true,
"usedInToolCall": true,
"usedInQueryExtension": true,
"toolChoice": true,
"functionCall": true,
"customCQPrompt": "",
"customExtractPrompt": "",
"defaultSystemChatPrompt": "",
"defaultConfig": {}
},
{
"model": "gpt-4o",
"name": "ChatGPT-4o-Latest",
"avatar": "/imgs/model/openai.svg",
"maxContext": 128000,
"maxResponse": 8000,
"quoteMaxToken": 12000,
"maxTemperature": 1.0,
"charsPointsPrice": 0,

```
      "censor": false,
      "vision": true,
      "datasetProcess": true,
      "usedInClassify": true,
      "usedInExtractFields": true,
      "usedInToolCall": true,
      "usedInQueryExtension": true,
      "toolChoice": true,
      "functionCall": true,
      "customCQPrompt": "",
      "customExtractPrompt": "",
      "defaultSystemChatPrompt": "",
      "defaultConfig": {}
    }
  ],
  "vectorModels": [
    {
      "model": "milkey/m3e:latest",
      "name": "m3e",
      "avatar": "/imgs/model/openai.svg",
      "charsPointsPrice": 0,
      "defaultToken": 512,
      "maxToken": 3000,
      "weight": 100,
      "defaultConfig": {},
      "dbConfig": {},
      "queryConfig": {}
    },
    {
      "model": "shaw/dmeta-embedding-zh",
      "name": "shaw/dmeta-embedding-zh",
      "avatar": "/imgs/model/openai.svg",
      "charsPointsPrice": 0,
      "defaultToken": 512,
      "maxToken": 3000,
      "weight": 100,
      "dbConfig": {},
      "queryConfig": {}
    }
  ],
  "reRankModels": [
    {
      "model": "bge-reranker-base",
      "name": "检索重排-base",
```

"charsPointsPrice": 0,
"requestUrl": "http://reranker:6006/v1/rerank",
"requestAuth": "ACCESS_TOKEN"
}
],
"audioSpeechModels": [],
"whisperModel": {
"model": "whisper-1",
"name": "Whisper1",
"charsPointsPrice": 0
}
}Model Versions:

模型版本:

Download Addresses of Open-Source Models Supporting Local Offline Deployment

1. DeepSeek-V3

HuggingFace official repository: https://huggingface.co/deepseek-ai/DeepSeek-V3

Domestic ModelScope mirror (recommended for domestic server download): https://modelscope.cn/models/deepseek-ai/DeepSeek-V3/summaryHugging Fa...

Source code repository: https://github.com/deepseek-ai/DeepSeek-V3

2. GLM-4-plus (GLM-4-9B-Chat)

HuggingFace: https://huggingface.co/zai-org/glm-4-9b-chat

ModelScope domestic mirror: https://modelscope.cn/models/ZhipuAI/glm-4-9b-chat-hf/summary

3. Qwen-plus (Qwen2.5 series chat model)

HuggingFace organization homepage: https://huggingface.co/Qwen

ModelScope domestic download mirror: https://modelscope.cn/models/qwen/Qwen2.5-72B-Instruct/summary

Official open-source repository: https://github.com/QwenLM/Qwen2.5

4. shaw/dmeta-embedding-zh (Chinese embedding model)

Original HuggingFace weight repository: https://huggingface.co/DMetaSoul/Dmeta-embedding-zhOllama

Ollama official pull address: https://ollama.com/shaw/dmeta-embedding-zhOllama

Supplementary Deployment Note

The above four models provide complete open-source weights. After downloading all weight files to the physically isolated offline server inside the hospital, offline inference can be realized via the local One API gateway (http://localhost:3001/), with all outbound public network traffic of the storage server blocked to avoid patient data leakage.

Non-local-deployable closed-source models (no downloadable weights)

Doubao-Pro-128k:

Doubao-Pro-128k is a closed-source proprietary model; ByteDance does not release its original weight files for local offline deployment. In our intranet architecture, the local One API gateway only forwards de-identified clinical text to Volcano Engine's public cloud API via vendor keys.

ChatGPT-4o-Latest, GPT-3.5-Turbo: No open-source local weight files released; only vendor cloud API keys are configured in local One API for intranet forwarding, and all clinical text is fully de-identified before external transmission.5. FastGPT Knowledge Base Construction Details.

Native API calls to Doubao-Pro-128k, ChatGPT-4o-Latest and GPT-3.5-Turbo carry theoretical risks of patient privacy leakage. However, this study implements multi-layer pre-processing controls, binding legal contractual restrictions and an intranet forwarding architecture to completely block the outflow of all identifiable patient information, with no actual risk of real patient data disclosure. Below is a three-part breakdown covering potential privacy risks, tiered prevention measures and compliance grounds.

Full pre-transmission de-identification (core blocking measure)

Automated scrubbing removes all patient identifiable information from clinical text before it enters the One API forwarding pipeline:

Direct identifiers: All patient names, ID numbers, mobile phone numbers, hospitalization IDs, medical insurance numbers and detailed residential addresses are permanently deleted.

Indirect quasi-identifiers: Exact admission dates, ward labels, bed numbers and unique imaging serial numbers are uniformly blurred.

Only non-traceable pure medical descriptive text is retained, without any fields that could be linked to individual patients.

The anonymized text sent externally to the APIs of Doubao-Pro-128k, ChatGPT-4o-Latest and GPT-3.5-Turbo contains no patient traceable markers. Even if vendor audit logs were inadvertently exposed, no real patient could be matched to the transmitted text.

Isolation via hospital self-hosted intranet One API forwarding gateway

Doubao-Pro-128k does not support local offline deployment. All API requests are routed through the physically isolated intranet One API gateway (http://localhost:3001/), rather than direct public network connections from client terminals:

API keys are centrally managed within the intranet; only authorized servers are permitted to initiate model calls, and full-link audit logs are stored exclusively inside the hospital with no external export.

The gateway integrates a real-time secondary sensitive information interception module; forwarding is immediately terminated if any protected health information (PHI) missed by de-identification is detected.

All cross-network transmission adopts TLS 1.3 encryption. Only ciphertext of anonymized text travels over public network links, which cannot be intercepted or decoded by man-in-the-middle attackers.

Binding legal agreements regulating third-party data processing

3.6 您保证在使用本专用条款项下服务过程中，不得对火山引擎的服务有以下行为：1) 利用技术或其他手段破坏、扰乱本专用条款项下产品服务的运营；2) 以任何方式干扰或企图干扰任何产品服务或任何功能的正常运行，或者制作、发布、传播上述工具、方法等；3) 以任何方式试图从服务中提取数据、参数、代码或其他属于火山引擎的保密信息。

3.7 您的用户或您员工违反以上限制的，您应督促您用户或员工整改，由此给火山引擎造成损失的，您应承担赔偿责任。

3.8 火山引擎的产品和服务是按照现有技术和条件所能达到的现状提供的。火山引擎将尽最大努力确保产品和服务的可用性，除本合同或双方书面确认的其他规定外，火山引擎不作任何其他明示或暗示的陈述或保证。例如，火山引擎未声明或保产品和服务100%完整、准确、可靠、更新及时，也不保证产品/服不间断、系统稳定没有任何故障、内容完全安全或不侵权，您应根据其实际情况独立判断。您基于产品和服务生成的内容产生的任何知识产权纠纷由您自行处理并承担所有责任，由此给火山引擎造成损失的，您应当承担赔偿责任。您也同意，即使火山引擎提供的产品和服务存在瑕疵，但若上述瑕疵是当时行业技术水平所无法避免的，其将不被视为火山引擎违约，双方应友好协作共同解决问题。火山引擎的责任应以您在使用产品和服务时严格遵守本合同或其他有关操作指南、使用规范的文件为前提，否则，当产品和服务使用不当时，火山引擎将不承担相应的责任。

4. 数据访问和使用

4.1 除非您单独同意，本服务将不使用您提交给服务或使用服务输出的内容训练、重新训练或改进基础模型。您单独同意的情况包括：同意火山引擎的《数据授权使用协议》。

4.2 为履行法定合规要求，火山引擎有权采取技术手段或人工手段对您使用本服务的行为及信息进行审查，包括但不限于对您提交给服务或使用服务输出的内容进行审查，建立和改进风险过滤机制、违法内容特征库等。

4.3 您接入我们服务向公众提供内容服务的，您理解火山引擎提供自动化过滤审核能力，为了保障审核能力的稳定性，您授权火山引擎基于过滤审核内容建立和改进风险过滤机制、违法内容特征库。

4.4 您确保火山引擎基于您同意对前述信息内容的存储和使用不会侵犯任何第三方的合法权益。

4.5 您应对您的数据，即您试（使）用服务而向我们提供的文字、图片、声音、音频、视频、代码等数据（下称“您的数据”）的来源及内容负责，火山引擎提示您谨慎判断数据来源及内容的合法性（包括但不限于遵守适用的个人信息保护及数据跨境传输法律法规）。

5. 知识产权

5.1 背景知识产权。任何一方和/或其关联方在本合同签署之前所拥有的背景知识产权依然归属该方所有。为免疑义，豆包搜索和其包含的模型、软件等服务的所有权、知识产权归属火山引擎，包括不限于模型参数、算法、代码、框架结构、版面设计等。

5.2 算法、参数、源代码。火山引擎不提供有关服务的算法、参数、源代码。除本合同明确约定外，您对火山引擎提供的服务不得进行任何算法、参数、代码等的修改。由于您对火山引擎提供的服务进行任何算法、参数、代码等的改动而导致服务不能正常运行或服务效果降低的，火山引擎对此不承担责任，由此给火山引擎造成损失的，您应当承担赔偿责任。

5.3 在适用法律允许的范围内，您的数据的知识产权归属于您或者依法享有这些知识产权的权利人。

5.4 模型生成结果中包含联网能力全文搜索结果内容的，火山引擎已在生成结果中标识搜索结果来源。火山引擎不对搜索结果内容承担责任。

A corporate-level commercial Ark service contract was signed with VolcEngine, incorporating two mandatory binding clauses:

Without written approval from our hospital, VolcEngine is strictly prohibited from utilizing any input text submitted by our institution to train or optimize Doubao series models.

The vendor may only perform real-time inference computation; all original input and output data shall be permanently purged immediately after inference completes. Only minimal audit logs (excluding full clinical text) may be retained, and our institutional data shall never be shared, sold or reused for any third-party purposes.

IP whitelist access and an independent strongly isolated multi-tenant data sandbox are enabled; vendor operation and maintenance staff are denied access rights to complete request text content.4.

4. Ethical approval compliance

烟台毓璜顶医院伦理委员会审批件

批件号：烟毓医伦理审【 2024-608 】号

项目名称	大语言模型结合知识库在优化甲状腺结节 C-TIRADS 分级中的应用				
研究涉及科室	超声医学科中区		项目负责人	刘真	
采集样本或信息类型	甲状腺超声自由文本报告		采集样本或信息数量	建模库 11489 份，测试集 228 份	
是否涉及样本、信息与国内外合作使用	无		合作单位或公司	无	
表决情况	表决人数	同意	做必要修改后同意	不同意	中止或暂停已批准的试验
	11	11	0	0	0

项目简介

甲状腺癌全球发病率逐年升高，超声是甲状腺结节首选影像学筛查手段，但临床 C-TIRADS 分级存在显著医师间、院间主观判读差异，标准化报告体系建设存在难点。本研究基于检索增强生成（RAG）技术搭建 FastGPT 甲状腺专科知识库，系统对比 DeepSeek-V3、ChatGPT-4o-Latest、GPT-3.5-Turbo、Doubao-pro-128k、GLM-4-plus、Qwen-plus 六种主流大语言模型，仅依托清洗脱敏后的甲状腺超声纯文本特征描述，完成标准化 C-TIRADS 分级，定量评估各类模型与高年资超声专家分级的一致性、分级推理耗时。研究全程不开展任何临床干预、不接触患者本人，无需采集活体组织、血液等人体生物样本，不涉及人类遗传资源使用，无国内、国际合作单位。

本课题郑重承诺：本回顾性文本数据研究方案符合国内医疗数据保护法规、临床研究伦理标准与国际通用医学研究惯例，数据存储、调用、分析全过程严格遵循《个人信息保护法》，严格保护患者隐私，全程规避数据泄露风险。

项目实施日期：2024 年 06 月 01 日 —— 2025 年 06 月 30 日

是否签署知情同意书：□是 ☑否

涉及人类遗传资源使用情况：无

国内外审批备案情况：无

国内及国际合作情况：无

科研伦理委员会意见

同意

2024-06-04 15：38

审批意见：

伦理委员会于 2024 年 06 月 04 日对上述实施方案进行认真讨论并表决，认为主要研究者具备超声临床科研资质，研究方案设计科学、合理、可行；本研究仅使用完全脱敏后的历史超声文本报告，无患者直接接触、无侵入性临床操作，患者隐私保护措施完善，研究潜在风险远低于研究预期临床价值；全部 AI 模型院内本地离线部署，符合医疗数据安全管理规范，同意按该方案开展回顾性研究。

本批文有效期自签字之日起一年。

主任委员签名：

会议地点：烟台毓璜顶医院行政楼 518 楼会议室

2024 年 06 月 04 日

This hybrid analytical workflow, integrating fully offline open-source large language models and intranet forwarding of de-identified clinical text to external commercial model APIs, has undergone complete ethical review and obtained formal approval from our institutional ethics committee. As clearly specified in the project profile of our ethical submission materials, this retrospective text-based research involves no clinical interventions, direct contact with patients, collection of human biological specimens, or utilization of human genetic resources. The ethics

committee explicitly approved the transmission of only fully anonymized, non-traceable thyroid ultrasound plain-text feature descriptions to external commercial large language models, while prohibiting any outbound transmission of identifiable patient information.

All data storage, retrieval and analysis procedures fully comply with the Personal Information Protection Law of the People's Republic of China. The hospital information technology department performs monthly security audits on One API invocation logs and de-identification interception records, and all complete audit trails are permanently retained within the hospital's isolated internal network to eliminate data leakage risks. We adopted a strict classified deployment strategy to minimize the scope of external data transmission: DeepSeek-V3, GLM-4-plus, Qwen-plus and the Chinese embedding model run entirely offline on physically isolated intranet servers of the hospital, with all outbound public network access blocked and zero clinical data outflow from the institution; only three closed-source commercial models (ChatGPT-4o-Latest, GPT-3.5-Turbo, Doubao-pro-128k) adopt the de-identified text forwarding pipeline.

中华人民共和国主席令

第九十一号

《中华人民共和国个人信息保护法》已由中华人民共和国第十三届全国人民代表大会常务委员会第三十次会议于 2021 年 8 月 20 日通过，现予公布，自 2021 年 11 月 1 日起施行。

中华人民共和国主席　习　近　平

2021 年 8 月 20 日

中华人民共和国个人信息保护法

（2021 年 8 月 20 日第十三届全国人民代表大会常务委员会第三十次会议通过）

目　　录

第一章　总　　则
第二章　个人信息处理规则
　第一节　一般规定
　第二节　敏感个人信息的处理规则
　第三节　国家机关处理个人信息的特别规定
第三章　个人信息跨境提供的规则
第四章　个人在个人信息处理活动中的权利
第五章　个人信息处理者的义务
第六章　履行个人信息保护职责的部门
第七章　法律责任
第八章　附　　则

第一章　总　　则

第一条　为了保护个人信息权益，规范个人信息处理活动，促进个人信息合理利用，根据宪法，制定本法。

第二条　自然人的个人信息受法律保护，任何组织、个人不得侵害自然人的个人信息权益。

第三条　在中华人民共和国境内处理自然人个人信息的活动，适用本法。

在中华人民共和国境外处理中华人民共和国境内自然人个人信息的活动，有下列情形之一的，也适用本法：

（一）以向境内自然人提供产品或者服务为目的；

All data processing workflows strictly comply with Articles 28 and 29 of the PIPL, as documented in our institutional ethics approval file.

#Steps to deploy FastGPT
cd opt
mkdir fastgpt

```
cd fastgpt
#All deployment operations were implemented by following the official self-host documentation published by the FastGPT development team.
#https://doc.fastgpt.io/zh-CN/guide/getting-started
curl -O
https://raw.githubusercontent.com/labring/FastGPT/main/files/deploy/fastgpt/docker-compose.yml
curl -O
https://raw.githubusercontent.com/labring/FastGPT/main/projects/app/data/config.json
#Edit the docker-compose.yml configuration file
version: '3.8'
services:
  pg:
    image: ankane/pgvector:v0.5.0 # git
    # image: registry.cn-hangzhou.aliyuncs.com/fastgpt/pgvector:v0.5.0 # 阿里云
    container_name: pg
    restart: always
    ports:
      - 5432:5432
    networks:
      - fastgpt
    environment:
      - POSTGRES_USER=fastgpt
      - POSTGRES_PASSWORD=123456
      - POSTGRES_DB=fastgpt
    volumes:
      - ./pg/data:/var/lib/postgresql/data
  mongo:
    image: mongo:5.0.18
    # image: registry.cn-hangzhou.aliyuncs.com/fastgpt/mongo:5.0.18 # 阿里云
    container_name: mongo
    ports:
      - 27017:27017
    networks:
      - fastgpt
    command: mongod --keyFile /data/mongodb.key --replSet rs0
    environment:
      - MONGO_INITDB_ROOT_USERNAME=fastgpt
      - MONGO_INITDB_ROOT_PASSWORD=123456
    volumes:
      - ./mongo/data:/data/db
      - ./mongodb.key:/data/mongodb.key
```

```yaml
  fastgpt:
    container_name: fastgpt
    image: ghcr.io/labring/fastgpt:v4.8.8 # git
    # image: registry.cn-hangzhou.aliyuncs.com/fastgpt/fastgpt:v4.8.8 # 阿里云
    ports:
      - 3002:3000
    networks:
      - fastgpt
    depends_on:
      - mongo
      - pg
    restart: always
    environment:
      - DEFAULT_ROOT_PSW=123456
      - OPENAI_BASE_URL=http://localhost:3001/v1
      - CHAT_API_KEY=sk-XXXXX
      - DB_MAX_LINK=5 # database max link
      - TOKEN_KEY=any
      - ROOT_KEY=root_key
      - FILE_TOKEN_KEY=filetoken
MONGODB_URI=mongodb://fastgpt:123456@mongo:27017/fastgpt?authSource=admin

      - PG_URL=postgresql://fastgpt:123456@pg:5432/fastgpt
    volumes:
      - ./config.json:/app/data/config.json
networks:
  fastgpt:
    name: fastgpt
    external: true
#set the name field to match the name configured in One API
#docker compose up -d
```

1. Overall Network & Isolation Architecture (Hospital Air-Gapped Intranet)

All computing nodes operate on a physically isolated internal hospital subnet with no persistent public network access:

All offline model servers block all outbound public network ports via hardware firewall rules; only the dedicated One API gateway node is permitted limited encrypted outbound traffic for anonymized commercial API requests via vendor IP whitelists.

Internal service communication: Private intranet static IP, isolated VLAN for AI inference services, no cross-segment access without preconfigured ACL rules.

Encryption rules: Inter-service traffic within the intranet uses TLS 1.3; all external API transmission enforces mTLS/HTTPS.

2. Software Stack

CUDA Version: 12.2

PyTorch: 2.4.1
Transformers: 4.45.1
vLLM inference engine v0.4.3 (containerized via official Docker image)
Docker image tag: vllm/vllm-openai:v0.4.3-cuda12.2
3. Local Service Endpoints (Intranet only)
DeepSeek-V3: http://localhost:8001/v1
GLM-4-plus: http://localhost:8002/v1
Qwen-plus : http://localhost:8003/v1
shaw/dmeta-embedding-zh embedding service: http://localhost:8004/v1
All model weights downloaded from HuggingFace/ModelScope are stored locally on isolated SSDs; no weight files are synced to external cloud storage. Public network egress is disabled during model runtime.
4.docker-compose.yml

```
# 数据库的默认账号和密码仅首次运行时设置有效
# 如果修改了账号密码，记得改数据库和项目连接参数，别只改一处~
# 该配置文件只是给快速启动，测试使用。正式使用，记得务必修改账号密码，以及调整合适的知识库参数，共享内存等。
# 如何无法访问 dockerhub 和 git，可以用阿里云（阿里云没有 arm 包）

services:
  # db
  pg:
    image: pgvector/pgvector:0.7.0-pg15 # docker hub
    # image: registry.cn-hangzhou.aliyuncs.com/fastgpt/pgvector:v0.7.0 # 阿里云
    container_name: pg
    restart: always
    ports: # 生产环境建议不要暴露
      - 5432:5432
    networks:
      - fastgpt
    environment:
      # 这里的配置只有首次运行生效。修改后，重启镜像是不会生效的。需要把持久化数据删除再重启，才有效果
      - POSTGRES_USER=username
      - POSTGRES_PASSWORD=password
      - POSTGRES_DB=postgres
    volumes:
      - ./pg/data:/var/lib/postgresql/data
  mongo:
    image: mongo:5.0.18 # dockerhub
    # image: registry.cn-hangzhou.aliyuncs.com/fastgpt/mongo:5.0.18 # 阿里云
    # image: mongo:4.4.29 # cpu 不支持 AVX 时候使用
    container_name: mongo
    restart: always
```

```
    ports:
      - 27017:27017
    networks:
      - fastgpt
    command: mongod --keyFile /data/mongodb.key --replSet rs0
    environment:
      - MONGO_INITDB_ROOT_USERNAME=myusername
      - MONGO_INITDB_ROOT_PASSWORD=mypassword
    volumes:
      - ./mongo/data:/data/db
    entrypoint:
      - bash
      - -c
      - |
        openssl rand -base64 128 > /data/mongodb.key
        chmod 400 /data/mongodb.key
        chown 999:999 /data/mongodb.key
        echo 'const isInited = rs.status().ok === 1
        if(!isInited){
          rs.initiate({
              _id: "rs0",
              members: [
                  { _id: 0, host: "mongo:27017" }
              ]
          })
        }' > /data/initReplicaSet.js
        # 启动 MongoDB 服务
        exec docker-entrypoint.sh "$$@" &

        # 等待 MongoDB 服务启动
        until mongo -u myusername -p mypassword --authenticationDatabase admin --eval "print('waited for connection')" > /dev/null 2>&1; do
          echo "Waiting for MongoDB to start..."
          sleep 2
        done

        # 执行初始化副本集的脚本
        mongo -u myusername -p mypassword --authenticationDatabase admin /data/initReplicaSet.js

        # 等待 docker-entrypoint.sh 脚本执行的 MongoDB 服务进程
        wait $$!

  # fastgpt
```

```yaml
  sandbox:
    container_name: sandbox
    image: ghcr.io/labring/fastgpt-sandbox:latest # git
    # image: registry.cn-hangzhou.aliyuncs.com/fastgpt/fastgpt-sandbox:latest # 阿里云
    networks:
      - fastgpt
    restart: always
  fastgpt:
    container_name: fastgpt
    image: ghcr.io/labring/fastgpt:v4.8.8 # git
    # image: registry.cn-hangzhou.aliyuncs.com/fastgpt/fastgpt:v4.8.8 # 阿里云
    ports:
      - 3000:3000
    networks:
      - fastgpt
    depends_on:
      - mongo
      - pg
      - sandbox
    restart: always
    environment:
      - DEFAULT_ROOT_PSW=1234
      # AI 模型的 API 地址哦。务必加 /v1。这里默认填写了 One Api 的访问地址。
      - OPENAI_BASE_URL=http://localhost:3001/v1
      # AI 模型的 API Key。（这里默认填写了 One API 的快速默认 key，测试通后，务必及时修改）
      - CHAT_API_KEY=sk-fXCgb85eAXyzy2yQ1b1e60E7Bb0a41B690D230192e7aDeA2
      # 数据库最大连接数
      - DB_MAX_LINK=30
      # 登录凭证密钥
      - TOKEN_KEY=any
      # root 的密钥，常用于升级时候的初始化请求
      - ROOT_KEY=root_key
      # 文件阅读加密
      - FILE_TOKEN_KEY=filetoken
      # MongoDB 连接参数. 用户名 myusername,密码 mypassword。
      - MONGODB_URI=mongodb://myusername:mypassword@mongo:27017/fastgpt?authSource=admin
      # pg 连接参数
      - PG_URL=postgresql://username:password@pg:5432/postgres
      # sandbox 地址
      - SANDBOX_URL=http://sandbox:3000
      # 日志等级: debug, info, warn, error
```

```yaml
      - LOG_LEVEL=info
      - STORE_LOG_LEVEL=warn
    volumes:
      - ./config.json:/app/data/config.json

  # oneapi
  mysql:
    # image: registry.cn-hangzhou.aliyuncs.com/fastgpt/mysql:8.0.36 # 阿里云
    image: mysql:8.0.36
    container_name: mysql
    restart: always
    ports:
      - 3306:3306
    networks:
      - fastgpt
    command: --default-authentication-plugin=mysql_native_password
    environment:
      # 默认 root 密码，仅首次运行有效
      MYSQL_ROOT_PASSWORD: oneapimmysql
      MYSQL_DATABASE: oneapi
    volumes:
      - ./mysql:/var/lib/mysql
  oneapi:
    container_name: oneapi
    image: ghcr.io/songquanpeng/one-api:latest
    # image: ghcr.io/songquanpeng/one-api:0.6.7
    # image: registry.cn-hangzhou.aliyuncs.com/fastgpt/one-api:v0.6.6 # 阿里云
    ports:
      - 3001:3000
    depends_on:
      - mysql
    networks:
      - fastgpt
    restart: always
    environment:
      # mysql 连接参数
      - SQL_DSN=root:oneapimmysql@tcp(mysql:3306)/oneapi
      # 登录凭证加密密钥
      - SESSION_SECRET=oneapikey
      # 内存缓存
      - MEMORY_CACHE_ENABLED=true
      # 启动聚合更新，减少数据交互频率
      - BATCH_UPDATE_ENABLED=true
      # 聚合更新时长
```

```
      - BATCH_UPDATE_INTERVAL=10
      # 初始化的 root 密钥（建议部署完后更改，否则容易泄露）
      - INITIAL_ROOT_TOKEN=fastgpt
    volumes:
      - ./oneapi:/data
networks:
  fastgpt:
services:
  one-api:
    image: "${REGISTRY:-docker.io}/justsong/one-api:latest"
    container_name: one-api
    restart: always
    command: --log-dir /app/logs
    ports:
      - "3000:3000"
    volumes:
      - ./data/oneapi:/data
      - ./logs:/app/logs
    environment:
      - SQL_DSN=oneapi:123456@tcp(db:3306)/one-api   # 修改此行，或注释掉以使用 SQLite 作为数据库
      - REDIS_CONN_STRING=redis://redis
      - SESSION_SECRET=random_string  # 修改为随机字符串
      - TZ=Asia/Shanghai
#      - NODE_TYPE=slave  # 多机部署时从节点取消注释该行
#      - SYNC_FREQUENCY=60  # 需要定期从数据库加载数据时取消注释该行
#      - FRONTEND_BASE_URL=https://openai.justsong.cn  # 多机部署时从节点取消注释该行
    depends_on:
      - redis
      - db
    healthcheck:
      test: [ "CMD-SHELL", "wget -q -O - http://localhost:3000/api/status | grep -o '\"success\":\\s*true' | awk -F: '{print $2}'" ]
      interval: 30s
      timeout: 10s
      retries: 3

  redis:
    image: "${REGISTRY:-docker.io}/redis:latest"
    container_name: redis
    restart: always

  db:
```

```
image: "${REGISTRY:-docker.io}/mysql:8.2.0"
restart: always
container_name: mysql
volumes:
  - ./data/mysql:/var/lib/mysql   # 挂载目录，持久化存储
ports:
  - '3306:3306'
environment:
  TZ: Asia/Shanghai     # 设置时区
  MYSQL_ROOT_PASSWORD: 'OneAPI@justsong' # 设置 root 用户的密码
  MYSQL_USER: oneapi     # 创建专用用户
  MYSQL_PASSWORD: '123456'      # 设置专用用户密码
  MYSQL_DATABASE: one-api     # 自动创建数据库
```

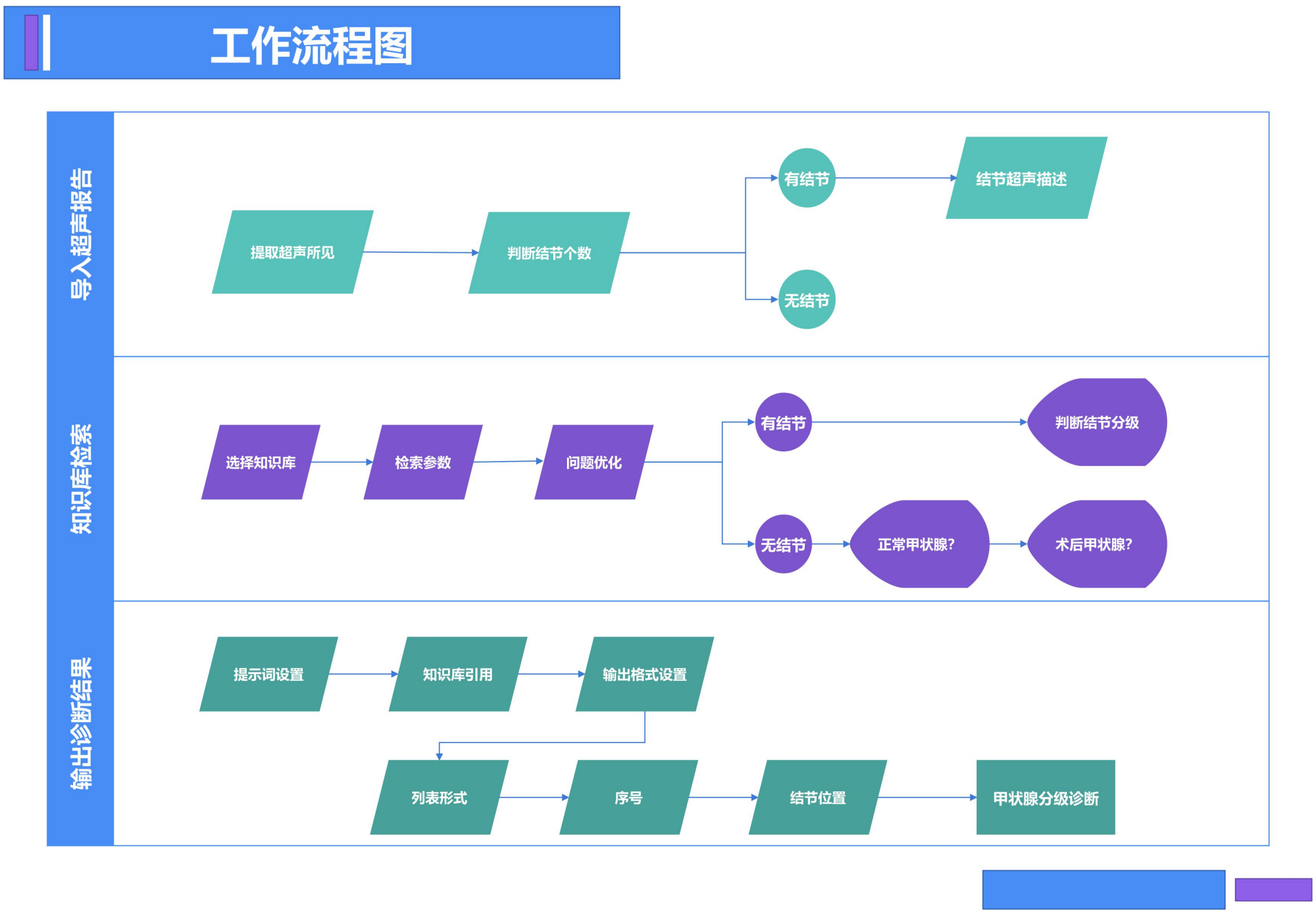

Vectorization Model: Shaw Chinese Vectorization Model (v1.0).
向量化模型：肖氏中文向量化模型（v1.0）
Text Chunking: Chunk size: 512 tokens; Overlap: 50 tokens.

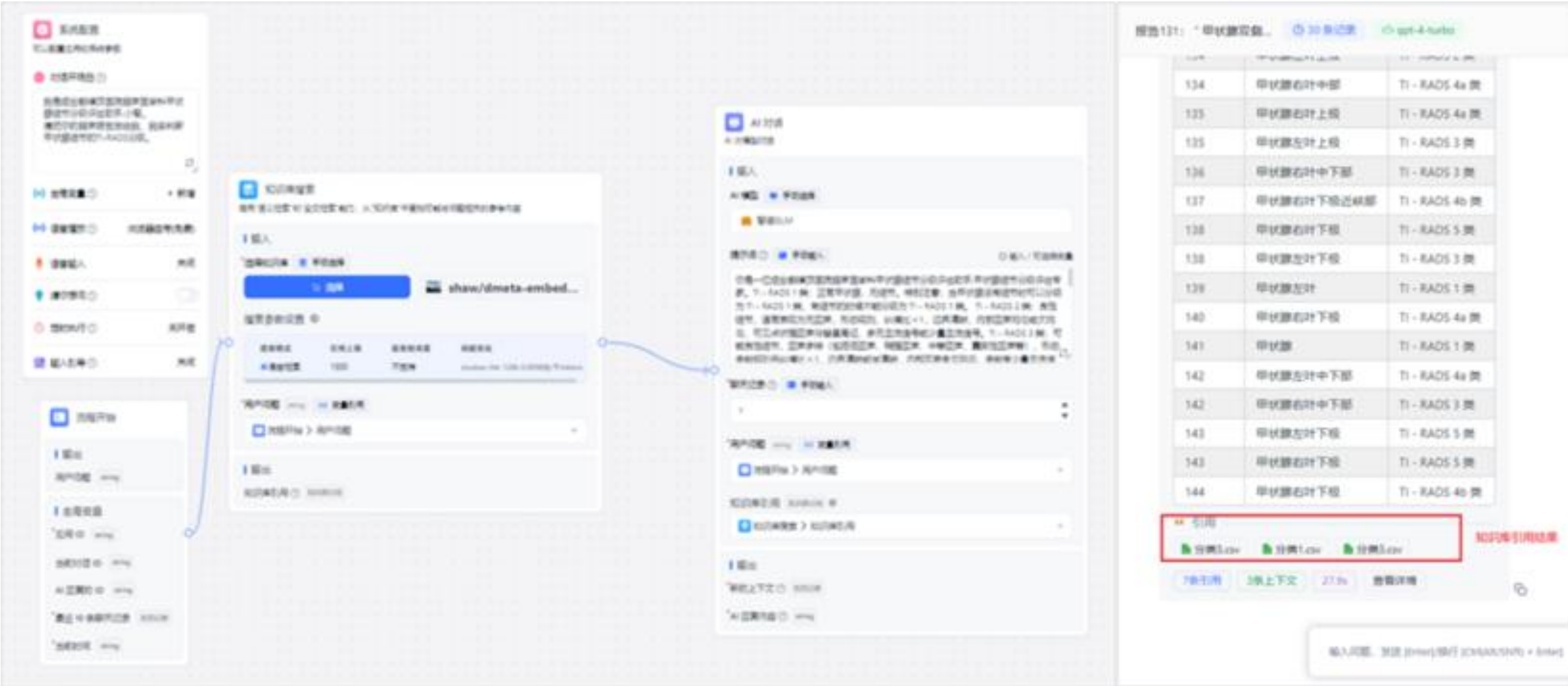

Retrieval Settings (RAG):

检索设置（检索增强生成，RAG）

Search Strategy: Hybrid Retrieval (Vector Semantic Retrieval + Full-Text Retrieval)

检索策略：混合检索（语义向量检索 + 全文检索）

Weight allocation: Semantic Retrieval = 0.5, Full-Text Retrieval = 0.5

权重分配：语义检索 0.5，全文检索 0.5

Fusion algorithm: Reciprocal Rank Fusion (RRF) for result sorting

融合排序算法：倒数排名融合（RRF）

Result Reranking: Enabled, Rerank Weight = 0.5

结果重排：已开启，重排权重 0.5

Filter Configurations

检索过滤参数

Citation Limit: 1500

引用上限：1500 条

Minimum Relevance Threshold: 0.9 (Cosine Similarity)

最低相关度阈值：0.9（余弦相似度）

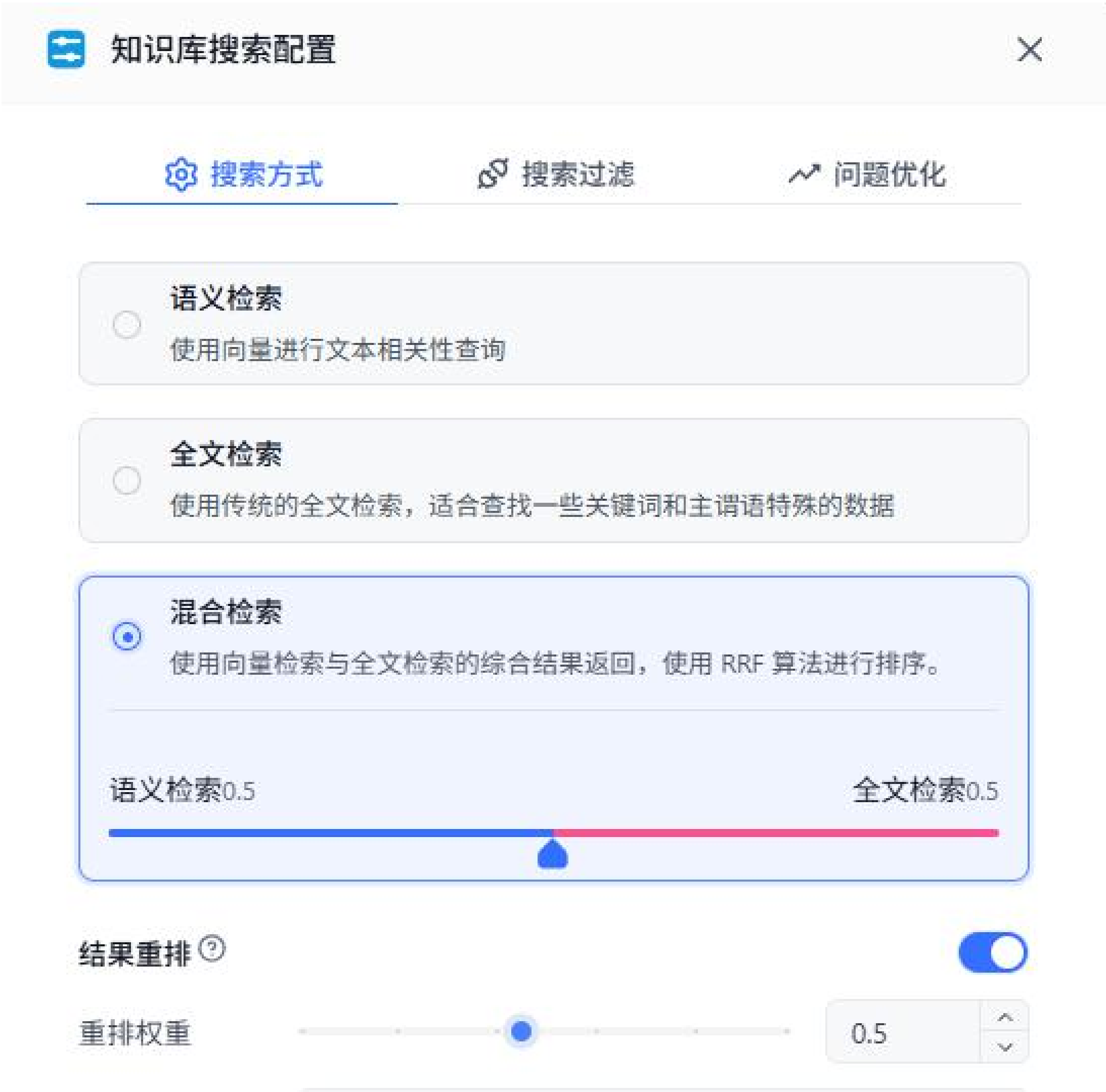

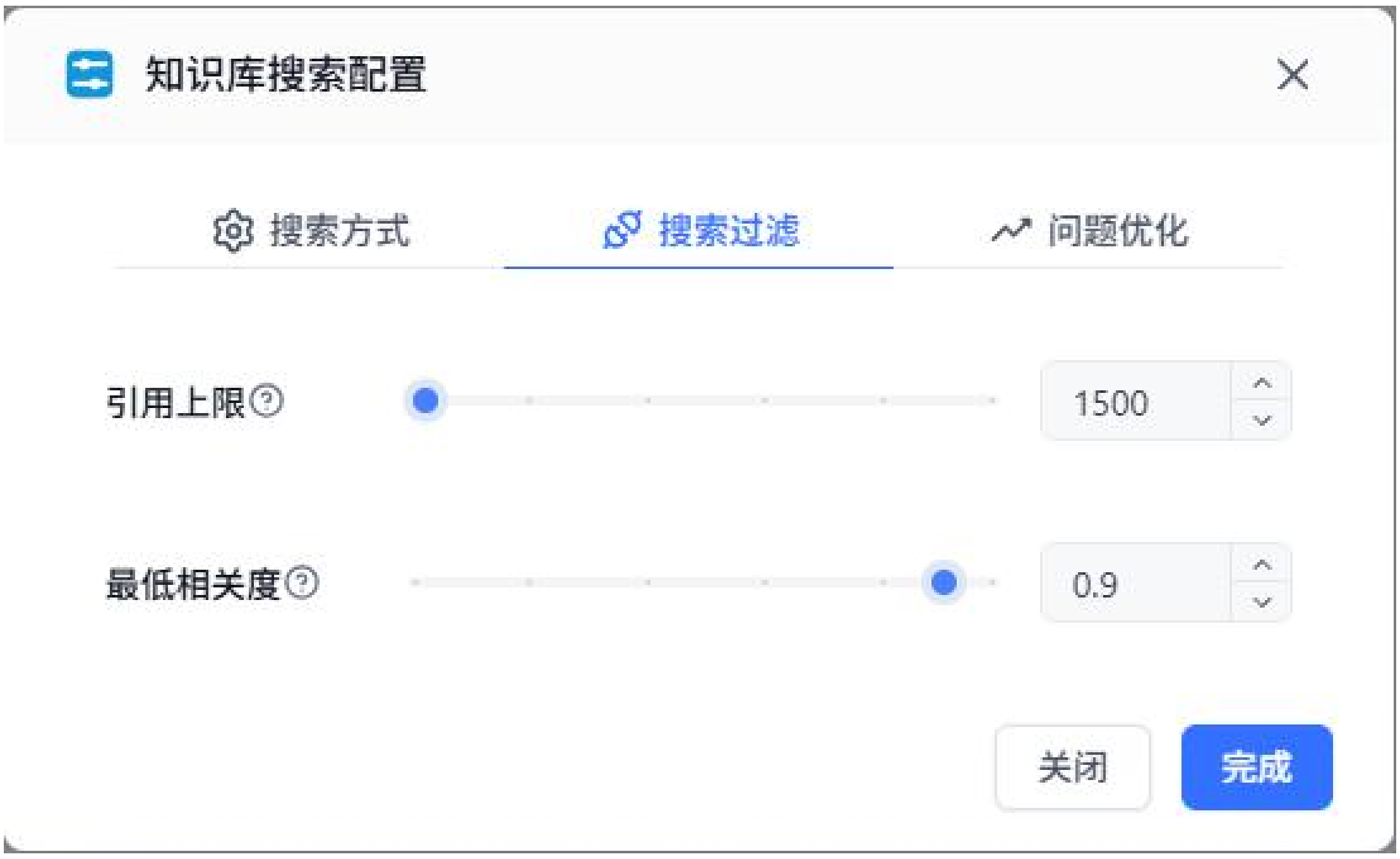

Failure Handling Rules:

故障处理规则:
If the model output did not contain "C-TIRADS" or a valid category number (1-5), the response was flagged as "Invalid".
如果模型输出内容未包含“C-TIRADS”或有效分类数字（1-5），该回复会被标记为“无效”
If the model refused to answer or hallucinated clinical history not present in the input, the run was repeated with a temperature of 0.0.
如果模型拒绝作答或虚构输入中不存在的临床病史，则将温度参数设为 0.0 重新运行该次测试。
In cases of persistent API errors (e.g., timeout), the case was logged and excluded from timing analysis but retained for concordance analysis using the last valid output.
若出现持续接口应用程序编程接口报错（例如请求超时），相关记录会被留存，不计入耗时分析，但会采用最后一次有效输出结果用于一致性分析。
6. Input/Output Examples
输入 / 输出示例
Input Example (Cleaned Text):
输入示例（清理后文本）:
"Right lobe upper pole solid nodule, size 0.9 x 0.7 x 0.6 cm. Margin is ill-defined. Internal echoes are markedly hypoechoic. Several tiny punctate strong echoes are noted within. Aspect ratio is 1.2. Color Doppler shows increased intranodular flow."

Output Example (Correct Response):
输出示例（正确回复）:

No.	Location	C-TIRADS Classification
1	Upper pole of left lobe nodule	C-TIRADS 2
1	Middle part of right lobe nodule	C-TIRADSy 2
2	Thyroid gland	C-TIRADS 1
3	Lower pole of left lobe nodule	C-TIRADS 4A
4	Middle part of left lobe nodule	C-TIRADS 2
5	Middle and lower part of left lobe nodule	C-TIRADS 3
5	Lower pole of right lobe nodule	C-TIRADS 3
6	Thyroid gland	C-TIRADS 1
7	Upper pole of left lobe nodule	C-TIRADS 3
7	Lower pole of left lobe nodule	C-TIRADS 3
7	Lower pole of right lobe nodule	C-TIRADS 3
7	Middle part of right lobe nodule	C-TIRADS 3
7	Isthmus nodule	C-TIRADS 3
8	Middle part of left lobe nodule	C-TIRADS 3
8	Middle and lower part of right lobe nodule	C-TIRADS 3
9	Thyroid gland	C-TIRADS 1
10	Middle and lower part of left lobe nodule	C-TIRADS 3
10	Middle part of right lobe nodule	C-TIRADS 2

序号	位置	C-TIRADS 分级
1	左叶上极结节	C-TIRADS 2 类

1	右叶中部结节	C-TIRADS 2 类
2	甲状腺	C-TIRADS 1 类
3	左叶下极结节	C-TIRADS 4A 类
4	左叶中部结节	C-TIRADS 2 类
5	左叶中下部结节	C-TIRADS 3 类
5	右叶下极结节	C-TIRADS 3 类
6	甲状腺	C-TIRADS 1 类
7	左叶上极结节	C-TIRADS 3 类
7	左叶下极结节	C-TIRADS 3 类
7	右叶下极结节	C-TIRADS 3 类
7	右叶中部结节	C-TIRADS 3 类
7	峡部结节	C-TIRADS 3 类
8	左叶中部结节	C-TIRADS 3 类
8	右叶中下部结节	C-TIRADS 3 类
9	甲状腺	C-TIRADS 1 类
10	左叶中下部结节	C-TIRADS 3 类
10	右叶中部结节	C-TIRADS 2 类

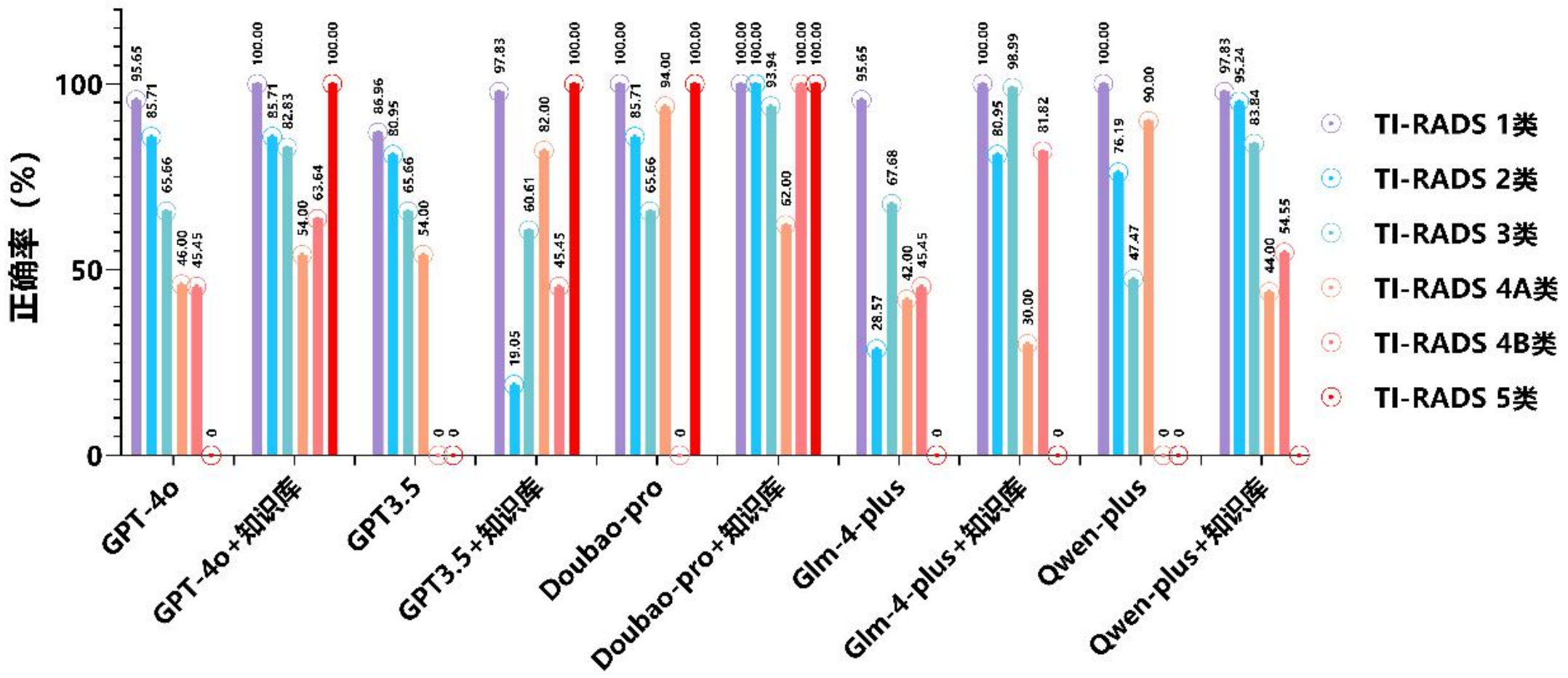

Output Example (Incorrect Response - Flagged for Error Analysis):
Exclude the grading of cervical lymph nodes, and summarize the following content in the format: | Report No. | Location | Full TI-RADS Classification |. The report number cannot be left blank and the corresponding number shall be filled in for identical entries. Three columns shall be listed, the full name of the classification shall be written with a unified format of "C-TIRADS Category X", and no explanations shall be provided.
剔除颈部淋巴结的分级,将下列内容按:|报告序号|位置|TI - RADS 分级|，进行总结，报告序号不能为空，相同的也要填上数字，列出三列,分级要写全称，分级格式统一为“C-TIRADS X 类”，不要回答原因。
7.Time Consumption for Thyroid Nodule Classification
7.甲状腺结节分类用时
The manual group was timed based on the report writing and review duration; the large language model group was timed based on the result generation duration.

人工组根据报个书写时间及审核时间计时，大语言模型组根据结果生成时间计时。

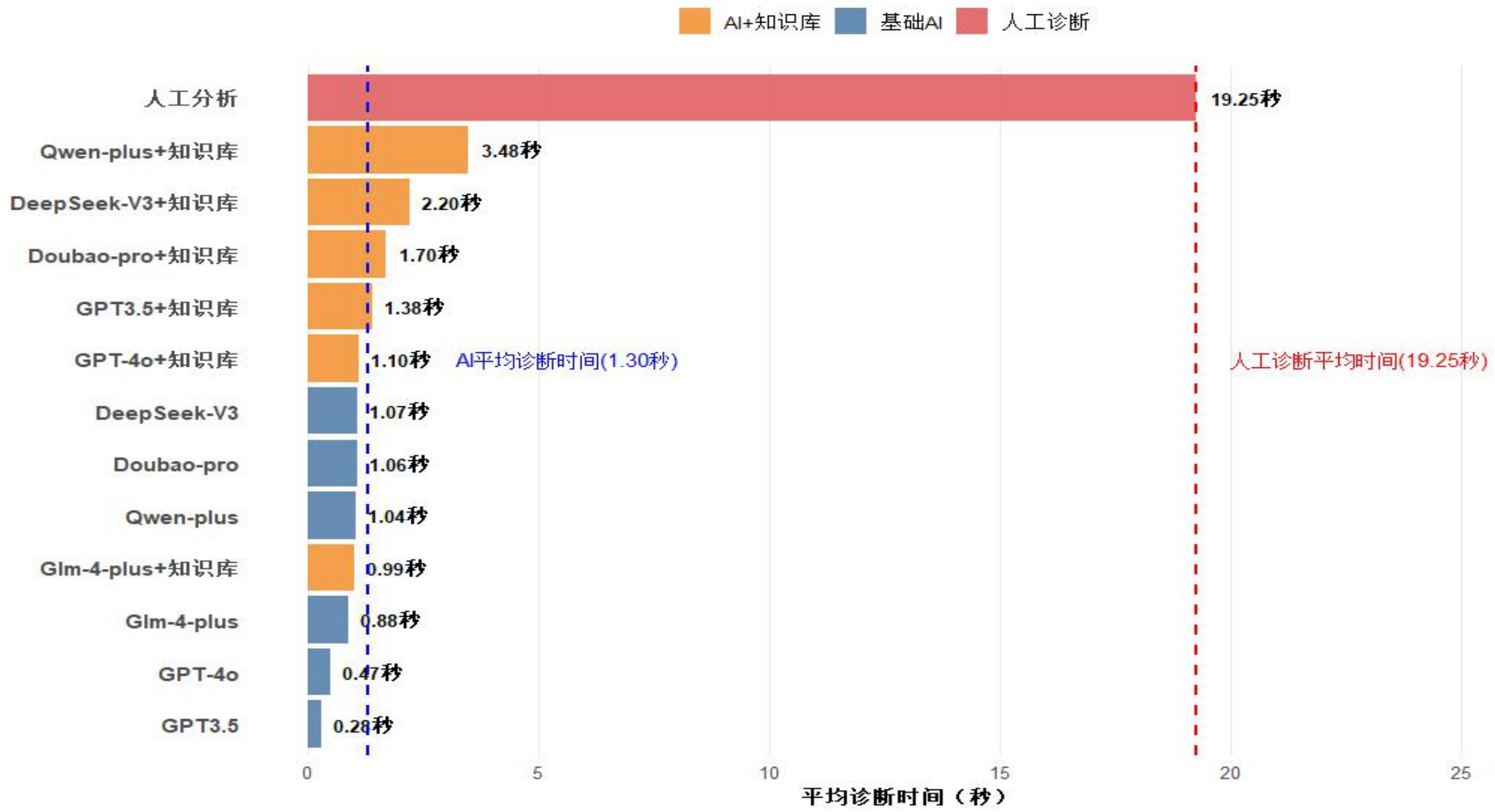

8.知识库搭建

数据源文件：dataset (2).csv

数据总量：2238 条数据，4476 条索引

文件大小：430.68 KB

字段结构：

index：序号索引

content：甲状腺超声文本报告（结节超声描述 +C- TIRADS 分级）

分块 / 索引配置：

分块大小：1000

索引大小：512

构建时间：2024-25-13 22:38，更新时间同步

构建流程全部完成，无异常（异常数量 = 0），阶段依次为：

内容解析中（Content Parsing）

索引向量化（Index Vectorization）

已就绪（Ready）

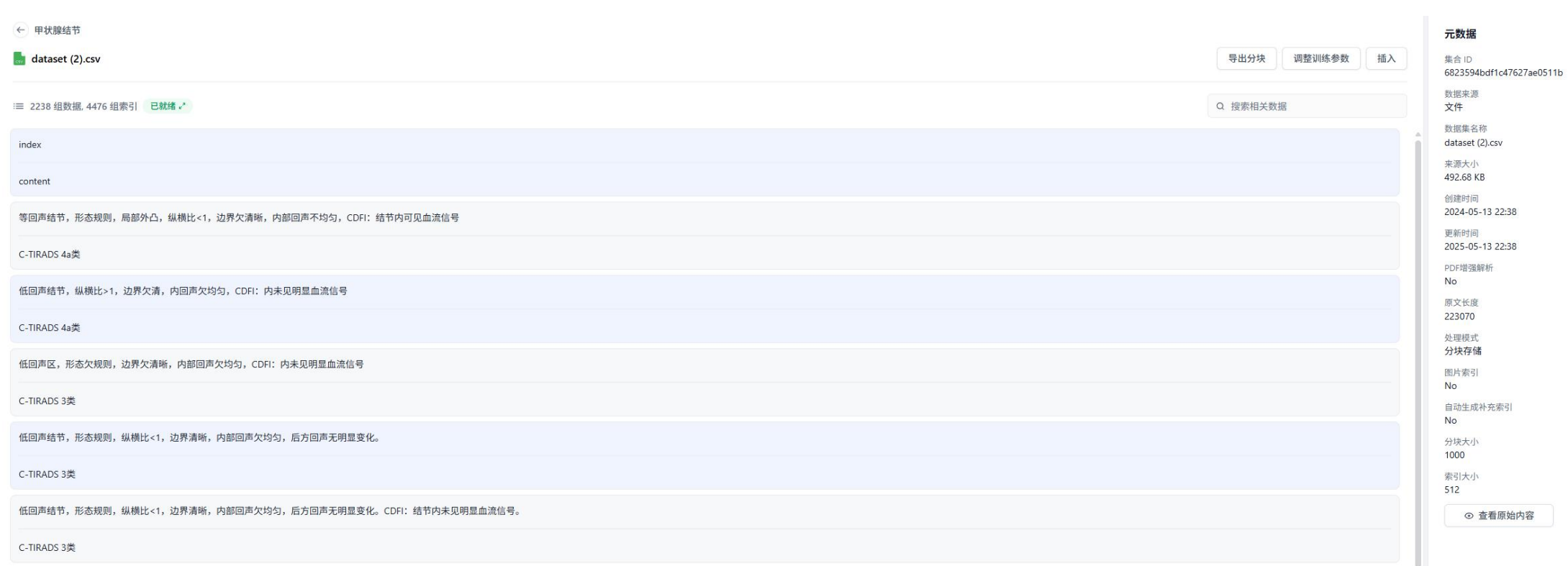

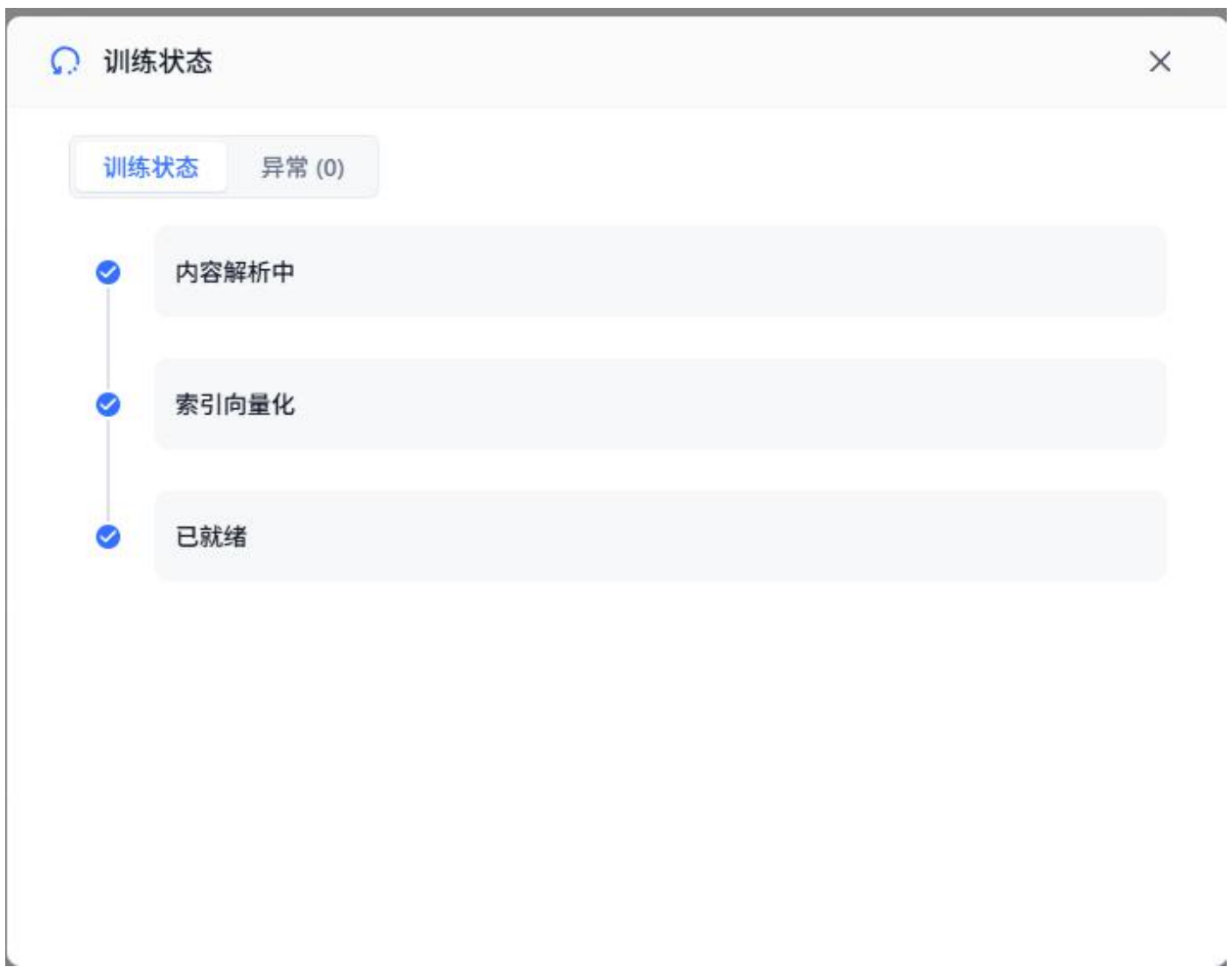

9. Statistical Code Availability

9.统计代码可用性

Analysis was performed using SPSS 26.0 and GraphPad Prism 9.5.0. The R script for the mixed-effects model (using lme4package) is available upon reasonable request to the corresponding author.

采用 SPSS 26.0 与 GraphPad Prism 9.5.0 软件完成数据分析。如需获取混合效应模型（基于 lme4 程序包）对应的 R 语言脚本，可向通讯作者提出合理申请。

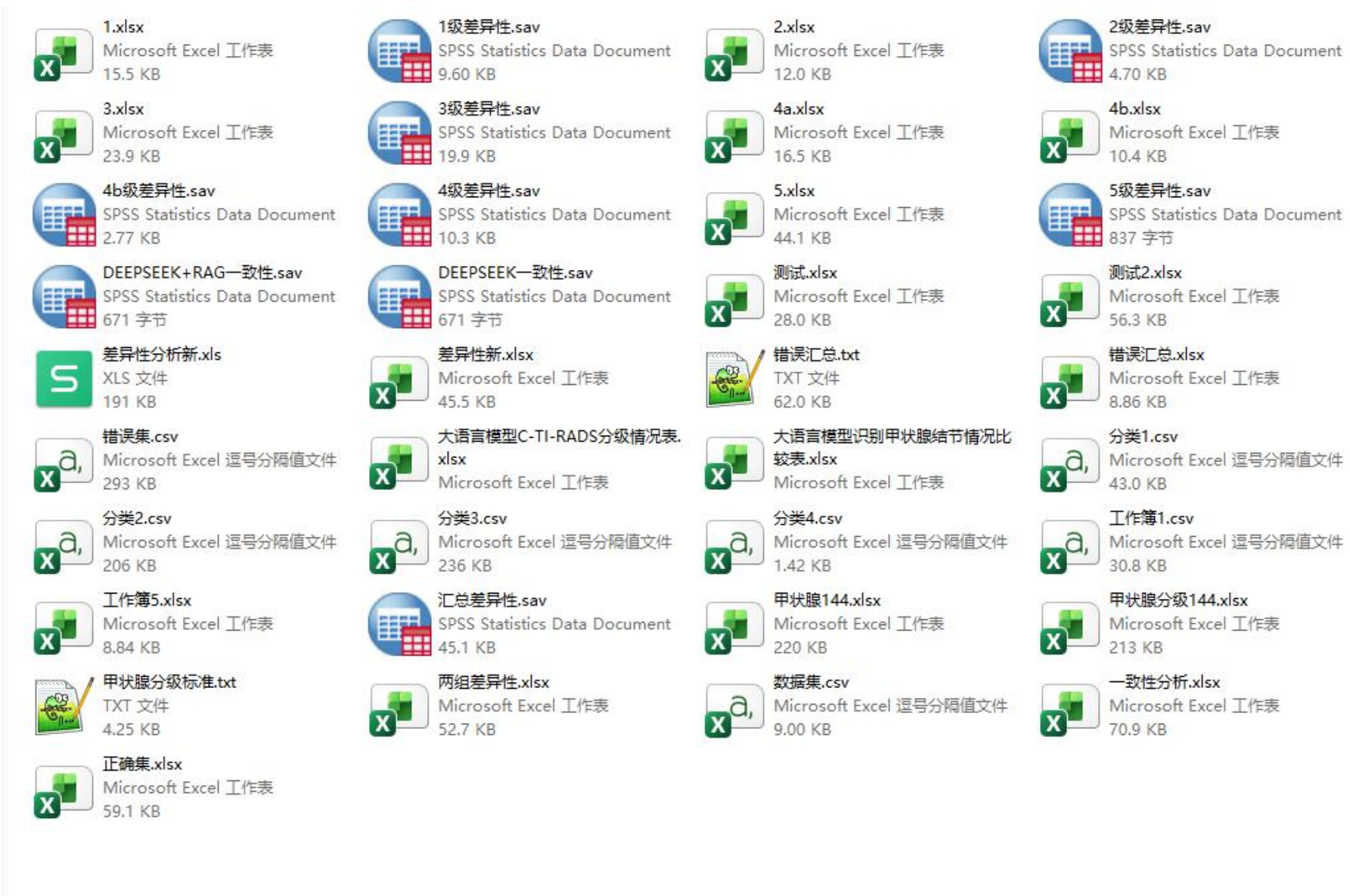

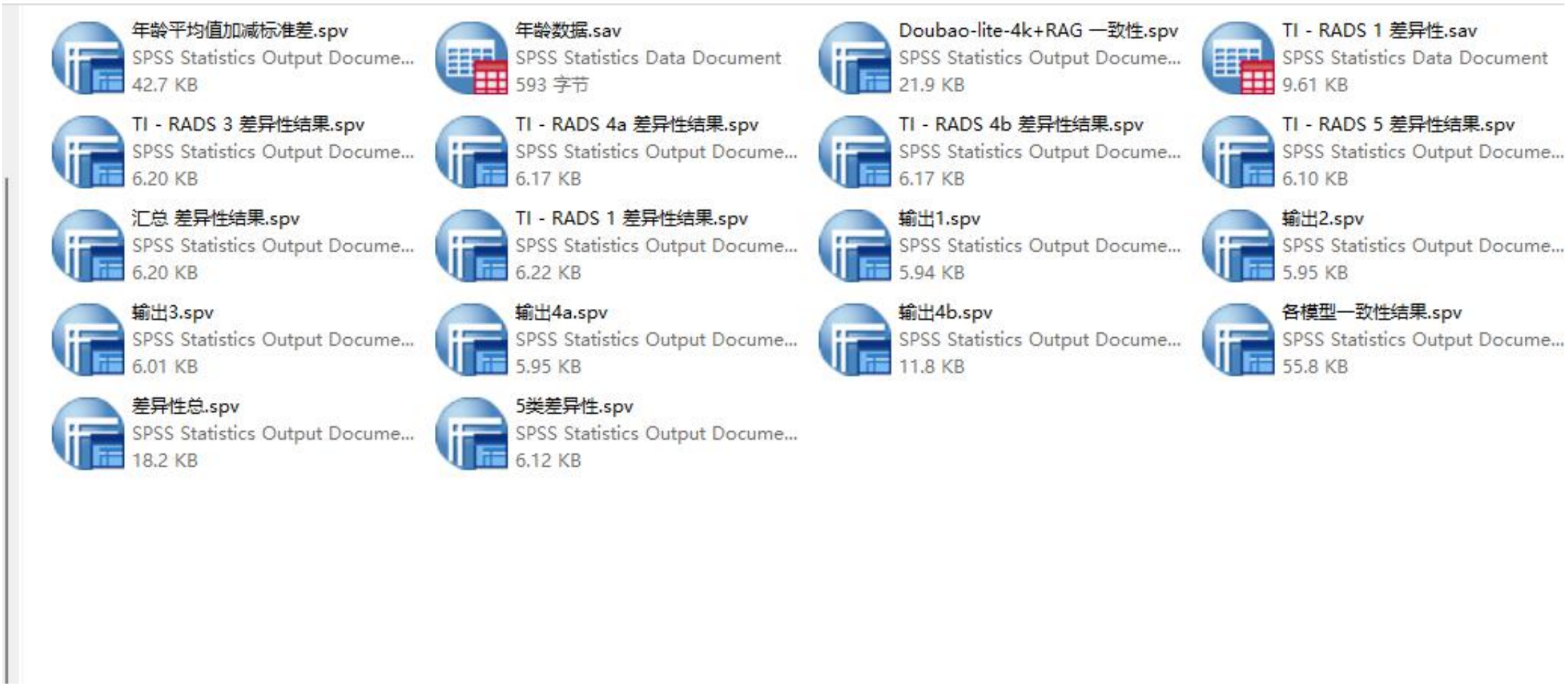
年龄平均值加减标准差.spv
SPSS Statistics Output Docume...
42.7 KB
年龄数据.sav
SPSS Statistics Data Document
593 字节
Doubao-lite-4k+RAG 一致性.spv
SPSS Statistics Output Docume...
21.9 KB
TI - RADS 1 差异性.sav
SPSS Statistics Data Document
9.61 KB
TI - RADS 3 差异性结果.spv
SPSS Statistics Output Docume...
6.20 KB
TI - RADS 4a 差异性结果.spv
SPSS Statistics Output Docume...
6.17 KB
TI - RADS 4b 差异性结果.spv
SPSS Statistics Output Docume...
6.17 KB
TI - RADS 5 差异性结果.spv
SPSS Statistics Output Docume...
6.10 KB
汇总 差异性结果.spv
SPSS Statistics Output Docume...
6.20 KB
TI - RADS 1 差异性结果.spv
SPSS Statistics Output Docume...
6.22 KB
输出1.spv
SPSS Statistics Output Docume...
5.94 KB
输出2.spv
SPSS Statistics Output Docume...
5.95 KB
输出3.spv
SPSS Statistics Output Docume...
6.01 KB
输出4a.spv
SPSS Statistics Output Docume...
5.95 KB
输出4b.spv
SPSS Statistics Output Docume...
11.8 KB
各模型一致性结果.spv
SPSS Statistics Output Docume...
55.8 KB
差异性总.spv
SPSS Statistics Output Docume...
18.2 KB
5类差异性.spv
SPSS Statistics Output Docume...
6.12 KB
